# Phase III study of long-term prognosis of estrogen receptor-positive early breast cancer treated with neoadjuvant endocrine therapy with/without adjuvant chemotherapy

**DOI:** 10.1007/s10549-023-06874-7

**Published:** 2023-03-22

**Authors:** Hiroji Iwata, Yutaka Yamamoto, Takehiko Sakai, Yoshie Hasegawa, Rikiya Nakamura, Hiromitsu Akabane, Shoichiro Ohtani, Masahiro Kashiwaba, Naruto Taira, Tatsuya Toyama, Tomomi Fujisawa, Norikazu Masuda, Yukiko Shibahara, Hironobu Sasano, Takuhiro Yamaguchi

**Affiliations:** 1 Breast Oncology, Aichi Cancer Center Hospital, 1-1 Kanokoden, Chikusa-ku, Nagoya, 464-8681 Japan; 2 Kumamoto University Hospital, 1-1-1 Honjo, Chuo-ku, Kumamoto, 860-8556 Japan; 3 Cancer Institute Hospital of Japanese Foundation for Cancer Research, 38-31 Ariake, Koto, Tokyo, 135-8550 Japan; 4 Hachinohe City Hospital, 3-1-1 Tamukai, Hachinohe, 031-8555 Japan; 5 Chiba Cancer Center, 666-2 Nitona-cho, Chuo-ku, Chiba, 260-8717 Japan; 6 Hokkaido P.W.F.A.C. Asahikawa-Kosei General Hospital, 1-24-111, Asahikawa, 078-8211 Japan; 7 Hiroshima City Hiroshima Citizens Hospital, 7-33 Motomachi, Naka-ku, Hiroshima, 730-8518 Japan; 8 Adachi Breast Clinic, 98 Kamigamo Matsumoto-cho, Kita-ku, Kyoto, 603-8052 Japan; 9 Kawasaki Medical School, 577 Matsushima, Kurashiki, Okayama 701-0192 Japan; 10 Nagoya City University Graduate School of Medical Sciences, 1 Kawasumi, Mizuho-cho, Mizuho-ku, Nagoya, 467-8601 Japan; 11 Gunma Prefectural Cancer Center, 617-1 Takahayashinishi-cho, Ota, Gunma 373-8550 Japan; 12 Nagoya University Graduate School of Medicine, 65 Tsurumai-cho, Showa-ku, Nagoya, 466-8550 Japan; 13 Tohoku University School of Medicine, 2-1 Seiryo-machi, Aoba-ku, Sendai, Miyagi 980-8575 Japan; 14 Present Address: Kitasato University, 1-15-1 Kitazato, Minami-ku, Sagamihara, Kanagawa 252-0373 Japan

**Keywords:** Neoadjuvant endocrine therapy, Early breast cancer, Chemotherapy, Response-guided therapy, Clinical trial

## Abstract

**Purpose:**

Neoadjuvant endocrine therapy (NET) is a treatment option for estrogen receptor-positive (ER+) postmenopausal early breast cancer (EBC). This phase III trial evaluated the prognosis of EBC patients treated with/without chemotherapy (CT) following NET.

**Methods:**

ER+/HER2−, T1c-2, and clinically node-negative EBC patients were enrolled in 2008–2013 and treated with endocrine therapy (ET) in weeks 24–28. All patients, excluding those with progressive disease (PD) during NET or ≥ 4 positive lymph nodes after surgery, were randomized to ET for 4.5–5 years with/without CT. The primary endpoint was disease-free survival (DFS). Secondary endpoints included distant DFS (DDFS), overall survival (OS), and DFS/DDFS/OS according to clinical response to NET.

**Results:**

Of 904 patients, 669 were randomized to CT+ET (*n* = 333) or ET alone (*n* = 336). The median follow-up was 7.8 years. DFS (CT+ET, 47 events; ET alone, 70 events) and DDFS did not reach the planned numbers of events. Eight-year DFS/DDFS rates were 86%/93% and 83%/92%, respectively. DFS was significantly better in CT+ET than ET alone in subgroups aged < 60 years (*P* = 0.016), T2 (*P* = 0.013), or Ki67 > 20% (*P* = 0.026). Progesterone receptor and histological grade were predictive markers for clinical responses to NET.

**Conclusion:**

NET may be used as standard treatment for patients with ER+EBC. Although it is difficult to decide whether to administer adjuvant CT based solely on the effect of NET, the response to NET may help to inform this decision.

**Trial registration:**

This study was registered at the UMIN Clinical Trials Registry under UMIN000001090 (registered 20 March 2008).

**Supplementary Information:**

The online version contains supplementary material available at 10.1007/s10549-023-06874-7.

## Introduction

Breast cancer is one of the most commonly diagnosed cancers worldwide [1]. Hormone receptor-positive, human epidermal growth factor receptor 2 (HER2)-negative disease accounts for more than 70% of incident breast cancer patients [2]. In the past three decades, survival outcomes of patients with early breast cancer (EBC) have notably improved, mainly due to early detection of the disease and advances in adjuvant treatments, such as endocrine therapy (ET), chemotherapy (CT), and anti-HER2 therapy. ET is the standard of care after surgery in patients with estrogen receptor-positive (ER+) breast cancer (luminal breast cancer) and current clinical data recommend longer treatment with ET in these patients [3, 4].

The St. Gallen conference in 2007 recommended ET alone and CT followed by ET for patients with highly or incompletely endocrine-responsive and HER2−, intermediate-risk breast cancer [5]. Many investigators have discussed the need for adjuvant CT in patients with ER+/HER2−, node-negative early-stage breast cancer, according to the multiple prognostic gene signature and other methods [6].

Recurrence score (RS), determined by a 21-gene signature, shows promise for identifying high-risk patients among patients with luminal breast cancer. The TAILORx study showed that adjuvant ET and chemoendocrine therapy had similar efficacies in postmenopausal women with ER+, HER2−, axillary node-negative breast cancer who had a mid-range RS, excluding patients aged ≤ 50 years [7]. The same study also reported a good prognosis in patients with a RS < 11, even without CT [8]. However, the 21-gene signature is costly to determine and is not covered by health insurance in many countries, and novel strategies are therefore required to determine the need for postoperative CT in patients with luminal node-negative breast cancer.

We therefore conducted a randomized phase III study (NEOS) to assess the long-term prognosis of patients with ER+EBC treated with neoadjuvant ET (NET) with/without adjuvant CT [6]. We previously reported the impact of NET on the health-related quality of life during NET and confirmed the feasibility of NET in patients with EBC [9]. In addition, we demonstrated that the 21-gene RS in core needle samples could be a predictive responsive marker of NET in the TransNEOS study [10]. Here we present the primary results of the NEOS trial with a longer median follow-up duration of 7.8 years.

## Methods

Detailed methods can be found in the study protocol (Supplementary file 1).

### Study design

This was an open-label, randomized, parallel-group controlled study involving patients responding to NET. Between 16 May 2008 and 7 June 2013, 904 patients from 100 institutions in Japan were enrolled and treated with letrozole (LET). The study consisted of preoperative and postoperative treatment periods [6].

### Patients

#### Preoperative enrollment

Postmenopausal women aged < 76 years with histologically diagnosed primary invasive ER+/HER2−, T1c-T2, N0 and M0 breast cancer were enrolled. The definition of ER+was ≥ 10% of cells stained by immunohistochemical assay at each local site in a pretreatment needle biopsy specimen. Patients with proven metastasis to a sentinel lymph node, synchronous or asynchronous bilateral breast cancer, multiple tumors located in multiple breast segments, double primary invasive cancer untreated or diagnosed within 5 years after completing the treatment for the previous cancer, a history of breast cancer, or ongoing treatment with any continuous systemic corticosteroid, any estrogen-containing agent, or any selective ER modulator were ineligible.

#### Postoperative enrollment

Patients with a clinical response to LET including complete response (CR), partial response (PR), or stable disease (SD), and who completed breast cancer surgical treatment, with lymph node-negative or -positive disease (1–3 nodes) were enrolled. If the primary tumor did not show progressive disease (PD), patients with 1–3 lymph node metastases were included in the randomized cohort without judging them as PD, assuming that they had metastasis from the beginning. Patients with HER2+ in a surgical specimen were excluded.

### Randomization and masking

Enrolled patients were randomized to receive either CT followed by LET or LET alone at an approximate ratio of 1:1 by dynamic allocation with stratification by the following factors: response to neoadjuvant LET therapy (CR or PR vs. SD), progesterone receptor (PgR) status at primary enrollment (positive vs. negative), pathological node status (positive vs. negative), age at primary enrollment (< 60 vs. ≥ 60 years), and study center.

### Procedures

During the preoperative period, eligible patients started a 24–28-week course of NET comprising once daily oral intake of 2.5 mg/day LET, within 4 weeks after enrollment. The clinical response to LET was evaluated at 1, 2, and 4 months after the start of the treatment by clinical examination and ultrasonography (US), and at the conclusion of treatment at 6 months by clinical examination, US, and either computed tomography or magnetic resonance imaging (MRI). The clinical response evaluation was only done for the primary tumor, and not for the lymph node area. If PD was identified, LET was discontinued and patients were treated and followed up in accordance with the pre-determined procedures.

Surgery for breast cancer was performed 1–4 weeks after the completion of NET. Patients who were eligible in the postoperative period were randomized to receive either CT followed by LET (CT+ET group) or LET alone (ET group) at a 1:1 ratio within 4 weeks after postoperative enrollment for 4.5–5 years. The treatment regimen for LET in both groups was the same as the regimen during the preoperative period.

### Outcomes

The primary endpoint was disease-free survival (DFS), defined as the time from the date of first registration until the date of the first event (recurrence in the ipsilateral preserved breast, ipsilateral chest wall or regional lymph node, or distant organ metastasis; secondary cancer without cutaneous basal cell carcinoma/spindle cell carcinoma and uterine carcinoma in situ; or all-cause death) in each group (CT+ET and ET). Secondary endpoints were the percentage of patients clinically responding to NET (CR or PR) and histological tumor response to NET, and DFS, distant DFS (DDFS), and overall survival (OS) in each group. Other secondary endpoints were the percentages of patients undergoing breast-conserving surgery, safety, health-related quality of life, and cost-effectiveness. DDFS was defined as the time to recurrence in distant organs such as the bone, liver, lung, brain excluding soft tissue or locoregional region, and breast cancer-unrelated death since randomization. OS was defined as the time from the date of primary enrollment until the date of death from any cause. Clinical staging, histological classification, and clinical response to NET were assessed in accordance with the General Rules for Clinical and Pathological Recording of Breast Cancer 15th Edition [11]. The Japanese version of the Eastern Cooperative Oncology Group scale was also used to grade performance status [12]. Clinical response was evaluated by inspection/palpation and US at each specified time point, and computed tomography or MRI was performed at the completion of NET. ER, PgR, and HER2 statuses and nuclear grade as eligibility criteria were assessed by local pathologists.

### Statistical analysis

The aim of this study was to determine if patients who responded to NET should choose ET alone or CT+ET as adjuvant therapy, in terms of DFS. To prepare for this analysis, all centers scheduled to participate in this study (*n* = 100) were sent a questionnaire and 78 of them responded. The results of the questionnaire survey were as follows: the mean predicted 5-year DFS with ET alone was 85% and the mean highest 5-year DFS with CT+ET that would strongly discourage oncologists to add adjuvant CT was 87% (condition A), whereas the mean lowest 5-year DFS with CT+ET that would strongly encourage oncologists to add adjuvant CT was 92% (condition B). Assuming an exponential distribution of DFS, the expected hazard ratios (HRs) for CT+ET relative to ET alone under conditions A and B were calculated to be 0.90 and 0.52, respectively.

Based on these survey results, the HR thresholds for choosing between the two treatments under conditions A and B were set at 0.9 and 0.6, respectively. A total of about 200 events were needed to provide a statistical power of 90% with these thresholds. Assuming that the 5-year DFS in the entire population was 88% and that 90% of the subjects would show CR, PR, or SD to NET, about 1,460 patients were needed to observe an occurrence of about 200 events during the planned follow-up of up to 8 years (a 3-year enrollment period plus a 5-year follow-up period). Accounting for an expected withdrawal of about 10% of the subjects, 1,700 patients were planned to be enrolled.

Patient enrollment did not proceed on schedule and the Study Steering Committee proposed postponing the enrollment period from 3 to 5 years and the total study period from 8 to 10 years in March 2011, which was approved by the Independent Data Monitoring Committee. We set the selection probability as 90%, but it was judged that it was possible to almost achieve the purpose of this study at about 80%–85%, in which case, about 170 events were required for both groups. In addition, assuming that the 5-year DFS for the overall population was 88%, the durations of the accrual and follow-up periods were 5 and 10 years (15 years for the longest follow-up period), respectively. A total sample size of 630 patients was required. Assuming that about a quarter of the preoperative patients were not registered in the second (postoperative) stage, the total sample size required was about 850 patients. Kaplan–Meier curves for DFS, DDFS, and OS were estimated for each response group. Median 8-year DFS/OS/DDFS with confidence intervals (CIs) were calculated based on the Greenwood’s formulae. A Cox’s proportional hazard model was used to investigate the relationships between clinical response to ET (CR, PR, or SD versus PD), DFS, DDFS, and OS. HRs with 95% CIs were obtained for DFS, DDFS, and OS. Logistic regression was used to investigate the relationships between predictive variables (age, body mass index, histological grade, Ki67, PgR, T, and HER2) and clinical responses. Given that this analysis was not performed at the pre-planned time and because further extension of the observation period would not achieve the planned event, we consulted with a biostatistician and decided to perform the analysis, even though the primary endpoint could not be proven with statistical certainty.

## Results

**Fig. 1:**
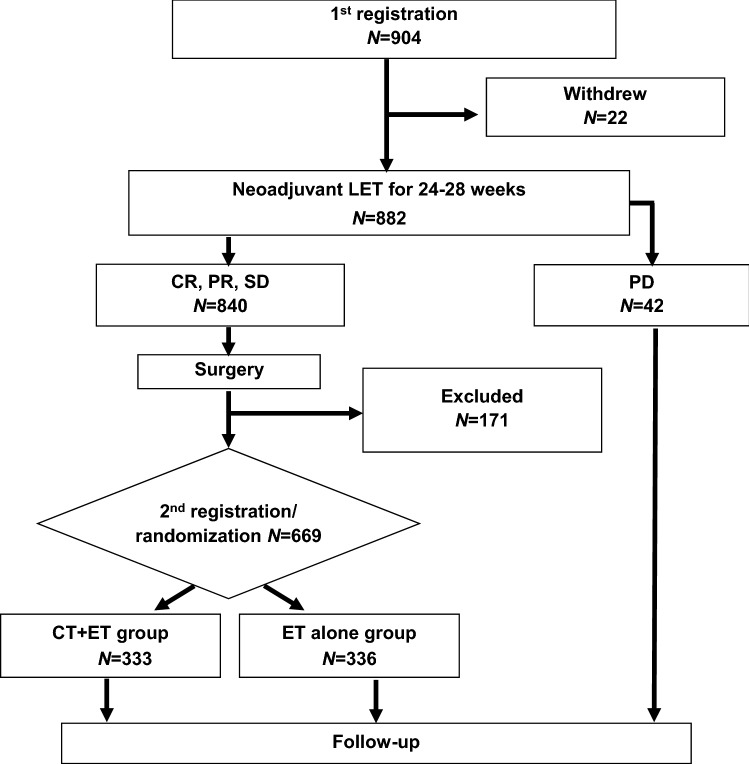
CONSORT diagram. *CR* complete response, *CT* chemotherapy, *ET* endocrine therapy, *LET* letrozole, *PD* progressive disease, *PR* partial response, *SD* stable disease

A total of 904 patients from 100 institutions in Japan were enrolled between 16 May 2008 and 7 June 2013, and treated with LET (Fig. 1). This study was terminated early because the number of events was unlikely to reach the planned number even after further observation. Among the 904 patients, 22 patients were withdrawn during NET because of patient request (15 patients), ineligibility (2 patients), transfer to another hospital (2 patients), and unknown reasons (3 patients). Among the remaining 882 patients who received NET, 15 patients (2%) showed CR, 422 (48%) showed PR, 403 (46%) showed SD, and 42 (5%) showed PD. The percentage of patients with a clinical response to NET (CR or PR) was 50% (437/882 patients). All PD patients had undergone complete resection of all residual disease following NET. Prior to randomization, 171 patients were excluded because they did not meet the postoperative enrollment criteria (65 patients: HER2+ in 8 patients; ≥ 4 positive lymph nodes in 8 CR or PR patients, 34 SD patients, and 8 SD patients with nuclear grade 3/vascular invasion, and other reasons in 7 patients), completion of the preoperative treatment in < 24 weeks for reasons other than PD (11 patients), refusal of surgery or postoperative protocol treatment (54 patients), patient’s preference (12 patients), other reasons (27 patients), and unknown reasons (2 patients). The 669 patients with a clinical response to NET of CR, PR, or SD and who completed breast cancer surgical treatment were assigned 1:1 to CT+ET or ET alone. The patients’ characteristics (age, tumor size, PgR status, nuclear grade, clinical and pathological responses to NET, and number of axillary lymph node metastases) were well balanced between the two groups (Table 1). The PgR+ and Ki67 < 20% rates were 81% and 60% in both groups, respectively. The CT regimens received by patients in the CT+ET group are summarized in Supplementary Table S1.

**Table 1:** Patient characteristics

	CT+ET (*N* = 333)	ET alone (*N* = 336)	*P* value
Age (at 2nd registration), years			
Mean (SD)	64 (5.6)	64 (5.8)	0.88
Median (min–max)	63 (49–76)	64 (50–76)	
< 60	76 (23%)	81 (24%)	0.70
≥ 60	257 (77%)	255 (76%)	
Clinical T stage			
T1c	136 (41%)	128 (38%)	0.47
T2	197 (59%)	208 (62%)	
PS			
0	331 (99%)	331 (99%)	0.26
1	2 (1%)	5 (1%)	
Menopausal status			
Pre	0	0	
Post	333 (100%)	336 (100%)	
ER status (local)			
Positive	333 (100%)	336 (100%)	
Negative	0	0	
PgR status (local)			
Positive	270 (81%)	272 (81%)	0.97
Negative	63 (19%)	64 (19%)	
HER2 status (IHC or FISH, local)			
Negative	333 (100%)	336 (100%)	
Positive (3+ or amplification)	0	0	
Histological grade			
1	83 (29%)	85 (29%)	0.98
2	193 (68%)	195 (67%)	
3	8 (3%)	9 (3%)	
Unknown	49	47	
Nuclear grade			
G1	223 (68%)	217 (67%)	0.36
G2	81 (25%)	76 (23%)	
G3	23 (7%)	33 (10%)	
Unknown	6	10	
Ki67 status (central)			
< 20%	173 (60%)	174 (60%)	0.94
≥ 20%	113 (40%)	115 (40%)	
Unknown	47	47	
Clinical response			
Complete response	7 (2%)	5 (1%)	0.84
Partial response	192 (57%)	195 (58%)	
Stable disease	134 (40%)	136 (40%)	
Surgical treatment			
Breast-conserving	280 (86%)	281 (84%)	0.53
Mastectomy	47 (14%)	54 (16%)	
Unknown	6	1	
Axillary dissection			
No	263 (81%)	268 (80%)	0.77
Yes	62 (19%)	67 (20%)	
Unknown	8	1	
Number of lymph node metastases			
0	276 (83%)	275 (82%)	0.67
1	48 (14%)	46 (14%)	
2	8 (2%)	13 (4%)	
3	1 (0.3%)	2 (1%)	

*CT* chemotherapy, *ER* estrogen receptor, *ET* endocrine therapy, *FISH* fluorescence in situ hybridization, *HER2* human epidermal growth factor receptor 2, *IHC* immunohistochemistry, *PgR* progesterone receptor, *PS* performance status, *SD* standard deviation

**Fig. 2:**
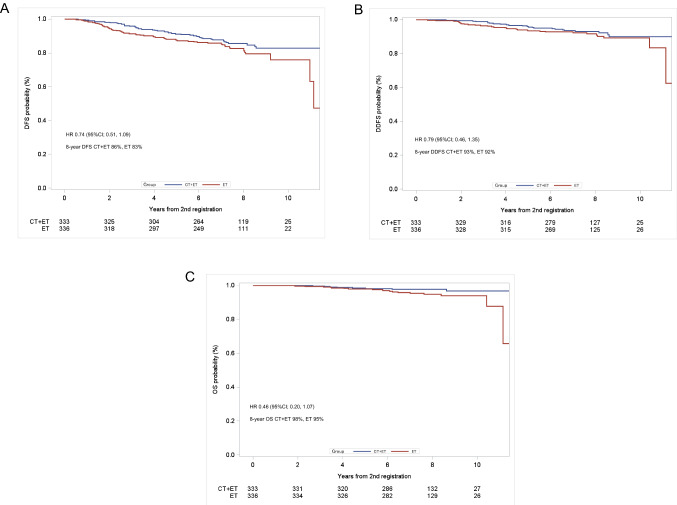
Outcomes in patients treated with CT+ET and ET. **A** DFS, **B** DDFS, and **C** OS. *CI* confidence interval, *CT* chemotherapy, *DDFS* distant disease-free survival, *DFS* disease-free survival, *ET* endocrine therapy, *HR* hazard ratio, *OS* overall survival

**Fig. 3:**
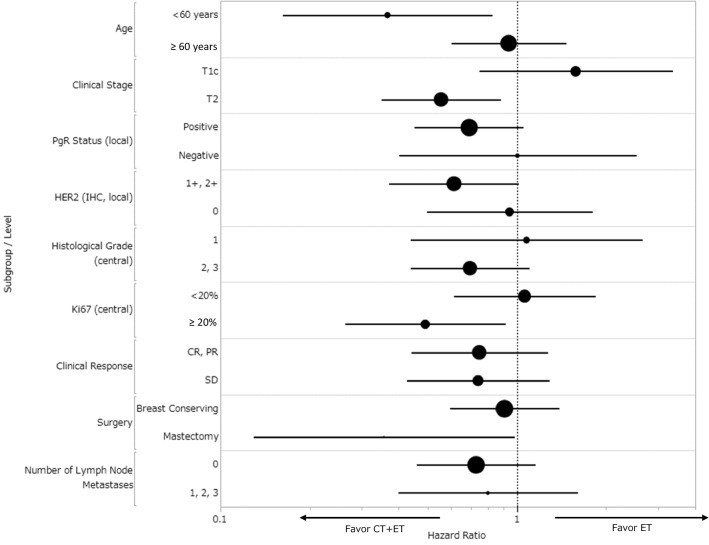
Subgroup analyses of DFS. *CR* complete response, *CT* chemotherapy, *DFS* disease-free survival, *ET* endocrine therapy, *HER2* human epidermal growth factor receptor 2, *IHC* immunohistochemistry, *PgR* progesterone receptor, *PR* partial response, *SD* stable disease

The median duration of follow-up was 7.8 years (range 0.1–19.6 years). Formal analysis of the primary end point was not possible because there were fewer DFS events (CT+ET 47, ET alone 70) than planned (HR 0.74 [95% CI 0.51, 1.09]) (Table 2, Fig. 2A). Similarly, there was no formal analysis of DDFS for the same reason (HR 0.79 [95% CI 0.46, 1.35]) (Fig. 2B). The DFS/DDFS rates at 8 years were 86%/93% and 83%/92% in the CT+ET and ET alone groups, respectively (Fig. 2A, B). There was no significant difference in OS between the two groups (HR 0.46 [95% CI 0.20, 1.07]) (Fig. 2C). DFS was significantly better in the CT+ET than in the ET alone group for some subgroups (Fig. 3): age < 60 years (HR 0.37,* P* = 0.016), clinical T2 (HR 0.56,* P* = 0.013), and Ki67 ≥ 20% patients (HR 0.49,* P* = 0.026) at baseline (Supplementary Fig. S1). However, there was no difference in DFS between CT+ET and ET alone in patients aged ≥ 60 years (HR 0.94,* P* = 0.78), T1c (HR 1.58,* P* = 0.23), and Ki67 < 20% (HR 1.06,* P* = 0.83) (Supplementary Fig. S2). There was no significant difference in DFS between the two groups according to response to NET (HR 0.75,* P* = 0.28 in CR and PR groups; HR 0.74,* P* = 0.29 in SD group) (Supplementary Fig. S3). Predictive markers of a clinical response to LET were determined by comparing clinical and pathological factors among patients with CR, PR, SD, and PD. Multivariate analyses showed that PgR (+ vs. −) and histological grade (3 vs. 1), but no other factors (age, body mass index, T, Ki67, or HER2), were markers of a clinical response to LET (Supplementary Table S2). No new adverse events were observed during NET and adjuvant therapy.

**Table 2:** Disease-free survival events among randomized patients

	CT+ET	ET alone
Total DFS events	47	70
Distant recurrence	14	19
Recurrence in ipsilateral preserved breast	3	4
Recurrence in in ipsilateral chest wall	0	6
Recurrence in regional lymph node	3	13
Any secondary cancer	26	24
Death due to other causes	1	4

*CT* chemotherapy, *DFS* disease-free survival, *ET* endocrine therapy

## Discussion

To the best of our knowledge, this was the first study to assess the value of response-guided therapy using NET. We found no difference in survival outcomes of patients with ER+EBC treated with NET, excluding patients with PD, compared with previous data for the same population [13]. Capecitabine and trastuzumab emtansine are standard treatments for patients with residual triple-negative and HER2+ breast cancer after neoadjuvant standard treatment, respectively, based on escalation studies in patients with non-pathological clinical response after neoadjuvant CT as the standard regimen [14, 15]. Response-guided therapy using neoadjuvant CT is currently a standard of care for patients with residual cancer with TN and HER2+. However, no de-escalation studies have excluded CT after neoadjuvant treatment for EBC, regardless of subtype. Furthermore, there are no confirmed data on NET-response-guided therapy for luminal-type breast cancer.

We considered that patients with luminal-type EBC who had PD following NET might have worse outcomes than those with CR, PR, and SD, based on the poorer prognosis of patients with endocrine primary resistance compared with those with a good response in patients with ER+metastatic breast cancer, and we therefore planned a randomized controlled trial to compare CT+ET and ET alone in patients with CR, PR, and SD, excluding PD, after NET. Previous small studies found different response outcomes to ET [16, 17].

The prognostic value of the clinical response to NET has previously been examined in small-scale studies [16, 17]. The correlation between tumor shrinkage by NET and survival has been reported in several studies [16, 17]. CR rates are generally lower in patients treated with NET than in those with neoadjuvant CT [18, 19]. Indeed, the CR rate was only 2%.

The results of this study show that it is difficult to decide whether to administer adjuvant CT based solely on the effect of NET. Among patients with CR, PR, or SD (excluding PD) following NET, the CT+ET group had better outcomes compared with the ET alone group, as demonstrated by the 8-year DFS/DDFS rates, especially among patients aged < 60 years or with clinical T2 or high proliferation. In a previous report, tumor size and high proliferation factors were shown to be predictive markers for benefit from CT [5, 20]. In contrast, however, the current results suggested that adjuvant CT may not be needed in patients with CR, PR, and SD by NET with clinical T1c or low proliferation at baseline. These results warrant further studies to determine the benefits of CT in patients who respond to NET.

The definitions of PR and SD used in this study were equivalent to the Response Evaluation Criteria in Solid Tumors (RECIST) criteria, and a reduced tumor size ≤ 30% and an increased tumor size ≤ 20% were both classified as SD. However, the possible differences in DFS and DDFS rates between these two groups in patients with SD should be investigated in future studies.

In this study DFS/DDFS were significantly better in the CT+ET compared with the ET alone group in patients aged < 60 years. The benefit of additional CT in patients aged < 60 years might be a result of the CT regimen, with more patients aged > 60 years receiving the cyclophosphamide, methotrexate, and fluorouracil regimen compared with the docetaxel and cyclophosphamide regimen (data not shown).

A previous study showed that Ki67 down-regulation at an early phase during NET and a preoperative endocrine prognostic index score were predictive markers for survival benefit in patients with ER+EBC [21–24]. However, it is currently unclear if these factors can determine the need for adjuvant CT.

Based on the results of a large-scale trial [7], multigene assays have been used to decide on the need for adjuvant CT. Adjuvant CT is not recommended in patients with postmenopausal EBC with pathological negative to three positive nodes with estimated low or intermediate RS [7, 25]. However, a multigene assay may not be a convenient tool because of its high cost. In contrast, NET is a convenient strategy worldwide because of its lower cost. In the TransNEOS study, using the same population as the current study, we validated the use of the RS to predict clinical response to NET [10]. In patients with T2 tumors and high proliferation at baseline, we recommend using the multigene assay before NET using a core needle sample. The response to NET may help to inform the decision of whether to use adjuvant CT.

No patients in this study experienced any serious adverse events or discontinued the study due to an adverse event during NET (data not shown), indicating that NET was safe and well-tolerated in these patients. The results of our previous study demonstrating that NET had no impact on health-related quality of life in EBC patients [9] further support our present findings on the safety and tolerability of NET using LET.

This study had some limitations, including low power due to the low incidence of DFS events, with 8-year DFS and OS rates of > 90% and > 95%, respectively, in the overall population, regardless of the relatively long follow-up period (7.8 years). There were many patients in this study who did not receive the prescribed treatment after randomization. In particular, 67 of the patients assigned to CT+ET did not receive chemotherapy (Supplementary Table S1). Of those who did receive chemotherapy, 63 received cyclophosphamide, methotrexate, and 5-fluorouracil (Supplementary Table S1). This may be one of the reasons why we could not show the benefit of receiving chemotherapy.

## Conclusion

NET may be used as standard treatment for patients with ER+EBC. Although it is difficult to decide whether to administer adjuvant CT based solely on the effect of NET, the response to NET may help to inform this decision.

## Supplementary Information

Below is the link to the electronic supplementary material.

Supplementary file1 (PDF 1159 KB) Study protocol

Supplementary file2 (PDF 358 KB)

Supplementary file3 (DOCX 12 KB)

## Data Availability

The data underlying the results presented in the study are available from CSPOR data center after publication (no end date). Data include individual de-identified participant data, a data dictionary as well as clinical trial protocols. Some restrictions apply due to confidentiality of patient data. Because these data are derived from a prospective clinical trial with ongoing follow-up collection there are legal and ethical restrictions to sharing sensitive patient-related data publicly. Data will be shared with researchers who provide a methodologically sound proposal to achieve aims in the approved proposal. Data can be requested in context of a research project sent to the corresponding author. Research proposals are approved by the NEOS steering committee.

## Abbreviations

CI: Confidence interval
CR: Complete response
CT: Chemotherapy
DDFS: Distant disease-free survival
DFS: Disease-free survival
EBC: Early breast cancer
ET: Endocrine therapy
HER2: Human epidermal growth factor receptor 2
HR: Hazard ratio
LET: Letrozole
MRI: Magnetic resonance imaging
NET: Neoadjuvant endocrine therapy
OS: Overall survival
PD: Progressive disease
PgR: Progesterone receptor
PR: Partial response
RS: Recurrence score
SD: Stable disease
US: Ultrasonography
