# Supplementary material for: Phase III study of long-term prognosis of estrogen receptor-positive early breast cancer treated with neoadjuvant endocrine therapy with/without adjuvant chemotherapy
Source: Breast Cancer Res Treat. 2023 Mar 22;199(2):231–41. doi: 10.1007/s10549-023-06874-7 (PMC10175450; doi:10.1007/s10549-023-06874-7)
Supplement: Supplementary file 1 — Supplementary file1 (PDF 1159 KB) Study protocol [file 10549_2023_6874_MOESM1_ESM.pdf]

N-SAS BC 06

National Surgical Adjuvant Study of Breast Cancer

CSPOR

Comprehensive Support Project for Oncology Research (CSPOR)
National Surgical Adjuvant Study of Breast Cancer (N-SAS BC)

Randomized Phase III Study of Adjuvant Endocrine-Therapy with or without Chemotherapy for Postmenopausal Breast Cancer Patients who Responded to Neoadjuvant Letrozole

Principal Investigator: Hiroji IWATA, Department of Breast Oncology, Aichi Cancer Center
1-1 Kanokoden, Chikusa-ku, Nagoya, Aichi 464-8681, JAPAN
Phone : 052-762-6111
Fax : 052-764-2963
E-mail: hiwata@aichi-cc.jp

Data Center : Data Management Office, General Affairs Department, Comprehensive Support Project (CSP), Public Health Research Foundation
1-1-7 Nishiwaseda, Shinjuku-ku, Tokyo 169-0051, Japan
Phone : 03-5287-2635
Fax : 03-5287-2644
E-mail: phrf-dm@csp.or.jp

Drafted: On March 28, 2007
Approved by the Independent Data Monitoring Committee: On January 25, 2008
Version 1.0 prepared : On February 1, 2008
Version 1.1 prepared: On September 1, 2010
Version 1.2 prepared: On March 25, 2011

Approved by Independent Data Monitoring Committee:	On May 31, 2012
Version 2.1 prepared:	On June 1, 2012
Version 2.2 prepared:	On February 1, 2021

Contents

0. Synopsis ... 4
1. Objectives ... 8
2. Backgrounds ... 9
3. Criteria and Definitions Used in This Study ... 18
4. Inclusion and Exclusion Criteria (for primary and secondary enrollment) ... 25
5. Enrollment and Treatment Plan ... 28
6. Drugs Used ... 36
7. Assessment of Adverse Events ... 38
8. Clinical and Laboratory Monitoring and Assessments ... 40
9. Plan for Follow-up of Patients with PD ... 50
10. Data Collection ... 52
11. Reporting of Adverse Events ... 53
12. HRQOL and Pharmacoeconomic Assessments ... 55
13. Central Pathological Review ... 63
14. Central Imaging Evaluation ... 65
15. Study Endpoints ... 66
16. Statistics ... 69
17. Ethics ... 74
18. Monitoring and Auditing ... 77
19. Publication of Study Findings ... 79
20. Conflict of Interest and Source of Funding ... 79
21. Clinical Study Registration ... 79
22. Related Studies ... 80
23. Study Organization and Administration ... 81
24. Protocol Changes and Study Termination ... 83
25. List of Study Centers ... 85
26. References ... 86

0. Synopsis

0.1 Study design and diagram

This will be a multicenter study with two-staged (preoperative and postoperative) enrollment and intervention. This will include an open-label, randomized, parallel-group controlled study involving patients responding to neoadjuvant endocrine therapy.

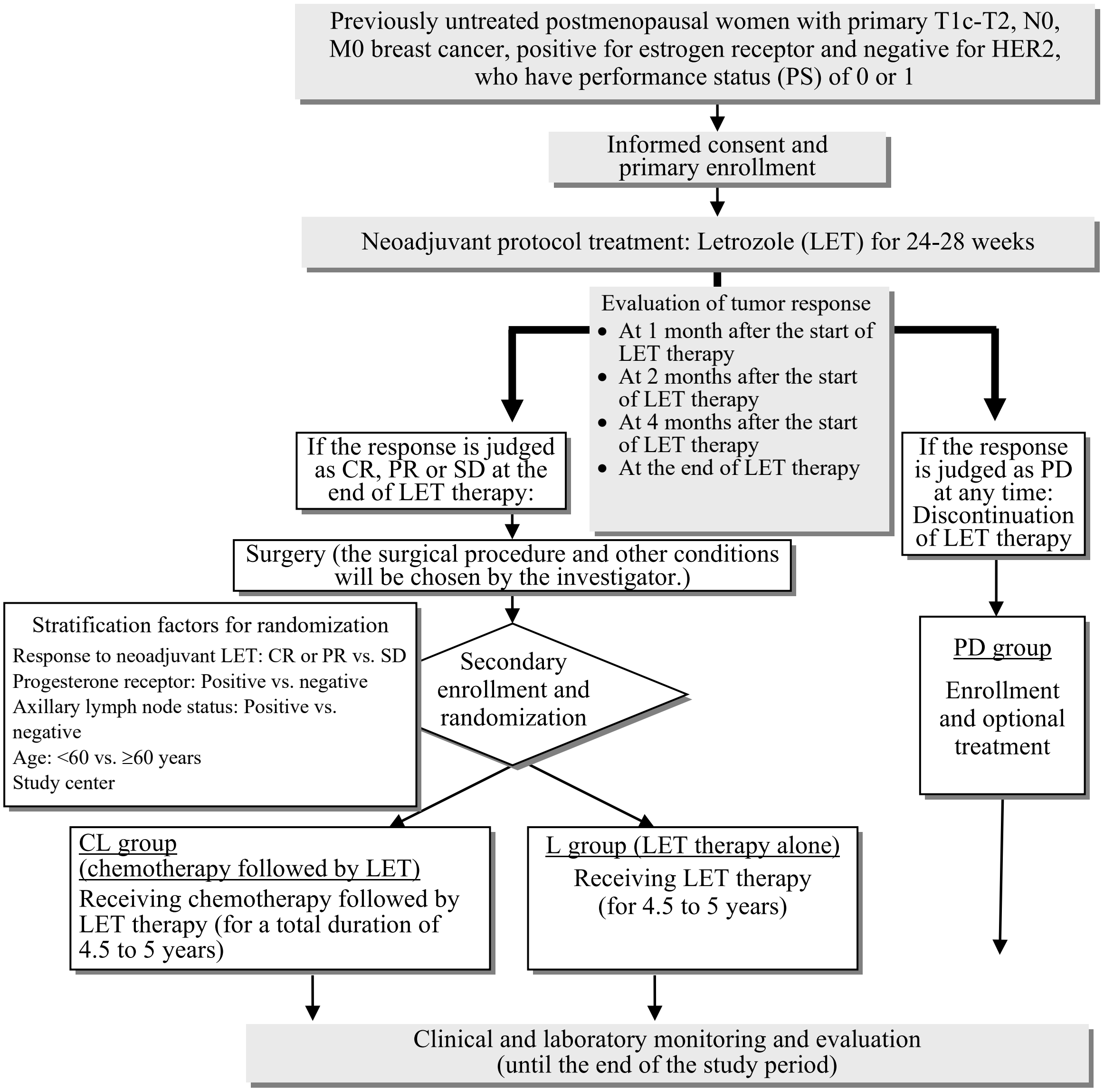

0.2 Objectives

1) To compare the disease-free survival (DFS) between the two randomized groups and thus to determine the need of postoperative adjuvant chemotherapy for patients responding to neoadjuvant LET.

2) In the context of the entire study, to determine the DFS and overall survival (OS) in different groups of all initially enrolled patients according to clinical response to neoadjuvant LET for evaluating the impact of neoadjuvant endocrine therapy on long-term prognosis of primary breast cancer.
3) To relate the clinical and histological tumor responses to neoadjuvant LET with the outcome of additional adjuvant chemotherapy in an exploratory manner.
4) To evaluate the effects of various treatments on health-related quality of life (HRQOL) and their cost-effectiveness (cost utility).
5) To promote clinical oncologists practicing in treating breast cancer to establish and expand their personal networks through participation in this clinical study.

Primary endpoint : DFS
Secondary endpoints : OS, distant disease-free survival (DDFS), percentage of patients clinically responding to neoadjuvant LET, histological tumor response to neoadjuvant LET, percentage of patients undergoing breast-preserving surgery, DFS/OS in patients showing CR, PR, SD or PD response to neoadjuvant LET, safety, HRQOL, and cost-effectiveness (cost utility).

0.3 Eligibility criteria used for primary enrollment

(1) Inclusion criteria; those who meet all of the following requirements will be considered for admission:

1) Postmenopausal women with histologically diagnosed primary invasive breast cancer.
   A woman will be considered postmenopausal if she:
   i) Is 60 years old or older,
   ii) Is under 60 years of age and had her last menstrual period more than 1 year ago (and if she has not undergone hysterectomy), and/or
   iii) Has undergone bilateral ovariectomy.

   Even if the above criteria cannot clarify the menopausal status, a woman will be considered postmenopausal if she has postmenopausal plasma levels of FSH (e.g., ≥30 mIU/mL) and estradiol (e.g., <10 pg/mL) designated at each center.
2) T1c-T2, N0, M0 carcinoma according to the TNM classification
   (See Section 3.1 Clinical staging.)
3) Positivity for estrogen receptor (≥10% cells stained in an immunohistochemical [IHC] assay) in a pretreatment needle biopsy specimen
4) Negativity for HER2 (2+ or a worse rating in an IHC assay or a negative FISH assay available) in a pretreatment needle biopsy specimen (Negativity for FISH assay, if the assay has been already obtained)
5) Age under 76 years at primary enrollment
6) PS graded as 0 or 1
   (According to the ECOG criteria; see Section 3.3 Performance status grading.)
7) No previous treatments of breast cancer at primary enrollment

8) Maintenance of adequate organ function as indicated by the following clinical and laboratory data (obtained within 4 weeks before primary enrollment):
   i) WBC ≥3,000/mm^3
   ii) Platelet count ≥100,000/mm^3
   iii) GOT/GPT ≤2.5 times the upper limit of normal (ULN) adopted at the center
   iv) Serum creatinine ≤1.5 mg/dL
   v) No treatments of ischemic stroke at enrollment
   vi) No history of myocardial infarction or congestive heart failure and absence of concurrent ischemic heart disease and valvular disease requiring any therapeutic intervention
9) Patient's informed consent to the study obtained after providing written information (Appendix A)

(2) Exclusion criteria; those who have any of the following conditions will be excluded from the study:

1) Proved metastasis to a sentinel lymph node, if biopsied before primary enrollment
2) Synchronous or asynchronous bilateral breast cancer
3) Multiple tumors located in multiple breast segments
4) Double primary invasive cancer untreated or diagnosed within 5 years after completion of treatment of the previous cancer
5) A history of breast cancer
6) Current treatment with any continuous systemic corticosteroid, any estrogen-containing agent, or any selective estrogen receptor modulator (SERM)
7) Current participation in any other clinical study of breast cancer
8) Any other condition that disqualifies the patient for inclusion in this study in the investigator's opinion

0.4 Eligibility criteria used for secondary enrollment

(1) Inclusion criteria; those who meet all of the following requirements will be considered for inclusion in the randomized part of the study:

1) Clinical response to the neoadjuvant protocol treatment (LET therapy) judged as CR, PR or SD (See Section 3.4.1 Clinical response rating criteria.)
2) Completion of any surgical treatment of breast cancer as scheduled in the protocol (See Section 5.6.)
3) The following lymph node status found after axillary lymph node dissection:
   i) Patients with CR or PR: No lymph node metastasis (node negative), or metastasis positive (1 to 3 nodes involved)
   ii) Patients with SD: No lymph node metastasis or metastasis positive (1 to 3 nodes involved), and the following criteria are met.

• Nuclear grade ≤ Grade 2
• No widespread invasion of the vasculature surrounding the tumor

However, a patient who does not undergo the lymph node dissection because of negative sentinel lymph node biopsy will be considered as “node negative

2) Exclusion criteria; those who have any of the following conditions will not enter the randomized part of the study:
   i) A positive FISH assay for HER2 (proved after primary enrollment) in a needle biopsy specimen
   ii) Patient’s refusal to enter the postoperative randomized part of the study

0.5 Protocol treatments

0.5.1 Neoadjuvant protocol treatment

Subjects will start the following adjuvant treatment within 4 weeks after the date of primary enrollment:

Oral once-daily treatment with LET at 2.5 mg/day for 24 to 28 weeks

0.5.2 Postoperative protocol treatments

Subjects will be randomized to one of the following two groups at secondary enrollment and will start the following postoperative adjuvant therapy within 4 weeks after the date of secondary enrollment:

**CL group (chemotherapy followed by LET)**	**:**	**Receiving chemotherapy followed by LET*<br>(for a total duration of 4.5 to 5 years).**
**L group (LET alone)**	**:**	**Receiving LET* (for 4.5 to 5 years)**

0.6 Planned sample size and study duration

Planned sample size	:	A total of 850 patients, 425 per group, are planned to be enrolled.
Enrollment	:	Until 5 years after enrollment of the first subject.
Follow-up	:	Until 10 years after enrollment of the last subject.
Study	:	For up to 15 years.

* Oral once-daily treatment with LET at 2.5 mg/day.

1. Objectives

This will be a multicenter study in postmenopausal patients with hormone-sensitive primary breast cancer with two-staged (preoperative and postoperative) enrollment and intervention. This will include a randomized controlled study involving patients responding to neoadjuvant endocrine therapy which is designed to compare chemotherapy followed by endocrine (letrozole [LET]) therapy (CL group) with endocrine (LET) therapy alone (L group) as postoperative adjuvant therapy.
The objectives of this study will include:

1) To compare the disease-free survival (DFS) between the two randomized groups and thus to determine the need of postoperative adjuvant chemotherapy for patients responding to neoadjuvant LET.
2) In the context of the entire study, to determine the DFS and overall survival (OS) in different groups of all initially enrolled patients according to clinical response to neoadjuvant LET for evaluating the impact of neoadjuvant endocrine therapy on long-term prognosis of primary breast cancer.
3) To relate the clinical and histological tumor responses to neoadjuvant LET with the outcome of additional adjuvant chemotherapy in an exploratory manner.
4) To evaluate the effects of various treatments on health-related quality of life (HRQOL) and their cost-effectiveness (cost utility).
5) To promote clinical oncologists practicing in treating breast cancer to establish and expand their personal networks through participation in this clinical study.

1.1 Primary endpoint

The primary endpoint of this study will be DFS.

1.2 Secondary endpoints

Secondary endpoints of this study will include OS, distant disease-free survival (DDFS), percentage of patients clinically responding to neoadjuvant LET, histological tumor response to neoadjuvant LET, percentage of patients undergoing breast-preserving surgery, DFS/OS in subgroups of patients according to CR, PR, SD or PD to neoadjuvant LET, safety, HRQOL, and cost-effectiveness (cost-utility).

1.3 Hypotheses to be tested in this study

1) Addition of chemotherapy does not improve the outcome of postoperative adjuvant therapy in postmenopausal women with HER2-negative, endocrine-responsive, node-negative primary breast cancer who have responded to neoadjuvant endocrine therapy.
2) Patients without disease progression (judged as achieving CR, PR or SD) during neoadjuvant endocrine therapy have better survival than those who do experience disease progression during the therapy.
3) Patients who receive postoperative adjuvant therapy with LET alone have better QOL than those who receive adjuvant chemotherapy followed by LET.

2. Backgrounds

2.1 Indication studied

2.1.1 Target illness

Breast cancer is a carcinoma with breast lobular or ductal origin. About 90% of breast cancer cases are classified as ductal carcinoma. Breast cancer is easy to spread to distant organs via the lymphatic and vascular systems at relatively early stages.
According to the Cancer Statistics in Japan 2007,[1] breast cancer was newly diagnosed in 40,675 Japanese persons and was the most prevalent carcinoma in Japanese women in 2001. As the number of breast cancer patients has been increasing since 1990 at a rate of more than 1,200 per year, about 48,000 Japanese persons will have newly diagnosed breast cancer in 2007. About one fourth the women with breast cancer die of this malignancy per year. Breast cancer is the fourth most frequent cause of cancer deaths in women in 2005, following colorectal cancer, gastric cancer, and lung cancer. This may reflect a relatively high survival probability for women with breast cancer.
When analyzed by age, the prevalence of breast cancer in women shows an increase from ages 30-39 years, reaches a peak at ages 45-49 or 50-54 years, and gradually becomes lower at higher ages. Trends of both breast cancer prevalence and mortality show consistent increases over years, resulting in higher prevalence and mortality rates in women born more recently. Comparison across countries shows that the prevalence rate is higher in Europe and the US (especially in Caucasians) than in East Asia and tends to be higher in Japanese American women than in native Japanese women living in Japan.
Patients with resectable, non-metastatic (stage I-IIIA) breast cancer usually receive systemic drug therapy postoperatively to control micrometastasis and to prevent recurrence. Drug therapy given for such purpose is referred to as postoperative drug therapy. Drug therapy may be given before surgical resection and is referred to neoadjuvant drug therapy. What regimen should be chosen as drug therapy depends on the patient’s risk of recurrence and age. Histopathological findings obtained by biopsy before drug therapy may be used to assist the choice of regimens.

2.1.2 Prognostic factors and predictors of response

Known prognostic factors for breast cancer include tumor size, axillary node status and number of nodes involved, hormone receptor expression status, histological grade (1 or 2 vs. 3), vascular invasion, and HER2 status (protein overexpression or gene amplification).[2] Of these factors, hormone receptor expression status predicts the response to endocrine therapy, while HER2 status predicts the response to trastuzumab.

2.1.3 Rationale for the study population

At the 2007 International Conference on Primary Therapy of Early Breast Cancer in St. Gallen,[2] endocrine therapy alone or chemotherapy followed by endocrine therapy was recommended as postoperative adjuvant therapy for highly endocrine-responsive, or incompletely endocrine-responsive AND HER2-negative, intermediate-risk breast cancer. However, any other characteristics of patients who need addition of chemotherapy and whether an aromatase inhibitor can replace tamoxifen as adjuvant endocrine therapy remain unknown.

Sufficient data have not been available about the long-term prognosis of patients receiving neoadjuvant endocrine therapy so that how the long-term treatment outcome differs between patients responding and not responding to neoadjuvant endocrine therapy and between those receiving and not receiving neoadjuvant endocrine therapy. With these backgrounds, the present study is designed to involve postmenopausal women with estrogen receptor (ER)-positive, T1c-T2N0M0 primary breast cancer, who are considered to be at intermediate risk for recurrence according to the 2007 St. Gallen Consensus Risk Classification[2] (Table 1). All patients enrolled are to receive neoadjuvant therapy with the aromatase inhibitor LET and those who do not experience disease progression during the neoadjuvant therapy are to be randomized to receive LET plus chemotherapy or LET alone postoperatively. DFS will be compared between the two randomized groups to determine which regimen is better as postoperative adjuvant therapy for this population.
Patients with HER2-positive breast cancer are not to be included in this study because patients with HER-2 positive disease are usually offered postoperative treatment with trastuzumab and respond well to anthracycline-based chemotherapy so that their assignment to the LET alone group would be ethically unacceptable. Even if a patient is found to be node-positive by postoperative pathological studies, she may be randomized if the number of nodes involved is not more than 3 according to the 2007 St. Gallen Consensus Risk Classification.

Table 1 Risk Classification of Patients with Operable Breast Cancer: 2007 St. Gallen Recommendation[2]

Risk categories	
Low risk	*Node negative* AND all of the following features:<br>Pathological tumor size (pT) ≤2 cm, AND<br>Grade 1, AND<br>Absence of peritumoral vascular invasion, AND<br>ER- and/or progesterone receptor (PgR)-positive, AND<br>HER2/*neu* gene neither overexpressed nor amplified, AND<br>Age ≥35 years
Intermediate risk	*Node negative* AND at least one of the following features:<br>Pathological tumor size (pT) >2 cm, OR<br>Grade 2-3, OR<br>Presence of peritumoral vascular invasion, OR<br>ER- and PgR-negative<br>HER2/*neu* gene overexpressed or amplified, OR<br>Age <35 years<br>*Node positive* (1-3 involved nodes) AND<br>ER- and/or PgR-positive, AND<br>HER2/neu gene neither overexpressed nor amplified
High risk	*Node positive* (1-3 involved nodes) AND<br>ER- and PgR-negative, OR<br>HER2/*neu* gene overexpressed or amplified<br>*Node positive* (4 or more involved nodes)

2.2 Standard therapy for the study population

Table 2 presents the regimens of postoperative drug therapy recommended for postmenopausal endocrine-responsive breast cancer at the 2005 International Expert Consensus Conference in St. Gallen.[3,4] Both endocrine therapy alone and chemotherapy plus endocrine therapy were recommended for the intermediate-risk group of patients with hormone-sensitive breast cancer. No major changes were made to the drug therapy recommendations for this group in St. Gallen Consensus 2007.

Table 2 Postoperative Drug Therapy Recommendations for Postmenopausal, HER2-negative, Endocrine-responsive Breast Cancer: St. Gallen Consensus 2005[3,4]

Risk groups	Endocrine-responsive, postmenopausal
Low risk	Tamoxifen or an aromatase inhibitor, or no therapy
Intermediate risk	Tamoxifen or an aromatase inhibitor, OR<br>Chemotherapy then tamoxifen, OR<br>Chemotherapy then an aromatase inhibitor,<br>Including tamoxifen for 2-3 years then exemestane or anastrozole, AND<br>Tamoxifen for 5 years then letrozole.<br>Chemotherapeutic regimens: AC, CMF, AC/A→CMF, FEC (every 3 weeks)<br>(AC/A→paclitaxel, FEC100→docetaxel, TAC)
High risk	Chemotherapy then tamoxifen, OR<br>Chemotherapy then an aromatase inhibitor<br>Including tamoxifen for 2-3 years then exemestane or anastrozole, AND<br>Tamoxifen for 5 years and then letrozole<br>Chemotherapeutic regimens: AC/A→CMF, CEF/CAF (days 1 & 8 every 4 weeks),<br>FEC (every 3 weeks), AC/A→paclitaxel,<br>FEC100→docetaxel, TAC (dose-dense regimen)

Five years' treatment with tamoxifen has been shown to be beneficial postoperative endocrine therapy for postmenopausal hormone-sensitive breast cancer.[5] More recently, selective aromatase inhibitors, including anastrozole, LET, and exemestane, have been used in postoperative adjuvant therapy and have been compared with 5 years' treatment with tamoxifen in many countries. In Japan, 5-year tamoxifen therapy has been compared with tamoxifen followed by anastrozole for a total duration of 5 years (N-SAS-BC03) or with tamoxifen for 2.5 to 3 years followed by exemestane for a total duration of 5 years (N-SAS BC-04) (a Japanese substudy of TEAM trial, which included an additional group receiving anastrozole for 5 years in Japan). Table 3 summarizes the results of representative randomized controlled studies of aromatase inhibitors that have been published when this study is planned.

Table 3 Representative Randomized Controlled Studies of Aromatase Inhibitors

Studies	ATAC[6]	IES[7]	BIG 1-98[8]	MA.17[9]
Aromatase inhibitor	ANA	EXE	LET	LET
Setting	Primary treatment	Sequential to 2-3 years' TAM therapy	Primary treatment *	Extended therapy after 5 years' TAM therapy
Randomized treatments	TAM vs. ANA (vs. ANA+TAM**)	TAM→TAM vs. EXE	TAM vs. LET (vs. TAM→LET vs. LET→TAM)	TAM→PLC vs. LET
No. of patients randomized	6,241 (TAM vs. ANA)	4,742	8,028***	5,170
ER status	84% ER+	81% ER+	98% ER+	97% ER+
Median duration of follow-up (months)	68	55.7	51	30
Relative risk reduction for recurrence	↓ 13% *P*=0.01	↓ 24% *P*=0.0001	↓ 18% *P*=0.007	↓ 42% *P*<0.001
Relative risk reduction for death	↓ 3% *P*=0.7	↓ 15% *P*=0.08	↓ 9% *P*=0.35	↓ 18% P=0.3
Adverse events	TAM / ANA	TAM / EXE	TAM / LET	PLC / LET
Hot flushes	40.9% / 35.7% *P*<0.0001	38.6% / 41.3% *P*=0.16	37.4% / 32.8% *P*<0.001	54% / 58% *P*=0.003
Genital bleeding	10.2% / 5.4% *P*<0.0001	6.5% / 4.6% *P*=0.008	8.3% / 3.8% *P*<0.001	8% / 6% *P*=0.005
Arthralgia	29.4% / 35.6% *P*<0.0001	15.1% / 20.8% *P*<0.0001	13.5% / 20.0% *P*<0.001	21% / 25% *P*<0.001
Fractures	7.7% / 11.0% *P*<0.0001	4.9% / 7.0% *P*=0.003	5.8% / 8.6% *P*<0.001	4.6% / 5.3% *P*=0.25
Thromboembolic events	4.5% / 2.8% *P*=0.0004	3.1% / 1.9% *P*=0.01	3.8% / 2.0% *P*<0.001	0.2% / 0.4% -
Ischemic cardiovascular disease	3.4% / 4.1% *P*=0.1	8.6% / 9.9% *P*=0.12	1.7% / 2.2% *P*=0.21	5.6% / 5.8%† *P*=0.76

*: Excluding the TAM→LET and LET→TAM groups. **: The study was prematurely terminated in patients assigned to the ANA+TAM group. ***: Including the numbers of patients receiving TAM in the TAM→LET group and those receiving LET in the LET→TAM group. †: Overall cardiovascular disease.
TAM: tamoxifen; ANA: anastrozole; EXE: exemestane; LET: letrozole; PLC: placebo.
Data are shaded for adverse events which occurred at significantly higher frequency in patients treated with an aromatase inhibitor than in the control group.

Based on these results, such international guidelines as those established by the American Society of Clinical Oncology (ASCO) expert panel,[10] the National Comprehensive Cancer Network (NCCN),[11] and St. Gallen International Consensus Conference[2] recommend the following regimens as the standard adjuvant endocrine therapy for postmenopausal hormone-sensitive breast cancer as of 2007: 5 years' treatment with an aromatase inhibitor, 2-3 years' treatment with tamoxifen followed by an aromatase inhibitor for a total duration of 5 years, 5 years' treatment with tamoxifen followed by 5 years' treatment with an aromatase inhibitor, and 5 years' treatment with tamoxifen.
In the absence of decisive evidence, no standard regimen of standard adjuvant chemotherapy has been established for postmenopausal hormone-sensitive breast cancer. Therefore, regimens generally accepted as standard adjuvant chemotherapy for breast cancer have been used in this population such as CMF, AC/EC, TC, AC/EC followed by paclitaxel/docetaxel, FEC/FAC, and FEC followed by docetaxel. No standard neoadjuvant therapy has been established for the study population when this study is planned.

2.3 Rationale for the protocol treatments

2.3.1 Drug under investigation

LET is a type II reversible aromatase inhibitor synthesized by Novartis (former Ciba-Geigy) in Switzerland. Compared with anastrozole, another agent in this class, LET is more potent in inhibiting aromatase and in suppressing estrogenic activity.[12,13]
Since its first approval in France in 1996 as second-line treatment of postmenopausal breast cancer, LET has been approved and used clinically in about 100 countries.[14] In Japan, its clinical development was begun in 1992 and a marketing application was filed in 2000 for the treatment of postmenopausal breast cancer with oral once-daily treatment at 1 mg. Subsequently, a large overseas clinical trial comparing LET with tamoxifen showed favorable outcomes with LET at 2.5 mg/day,[15] establishing this as the standard dose of LET. The efficacy and safety of LET at this dose was then evaluated in Japanese postmenopausal women with tamoxifen-resistant breast cancer. The study confirmed the efficacy and safety of LET at 2.5 mg/day in Japanese patients and the similarity of its efficacy/safety profiles between Japanese and non-Japanese patients. LET was approved by the Japanese health authority in January 2006 for the treatment of postmenopausal breast cancer at a 2.5 mg once-daily dosage, and was listed in the Japanese National Health Insurance Tariff in May 2006.
When this study is planned, LET has been used worldwide as first-line and second-line treatment of metastatic/recurrent breast cancer in postmenopausal women, as well as in neoadjuvant and adjuvant settings (primary treatment and sequential to 5 years' treatment with tamoxifen) for primary breast cancer in postmenopausal women.

2.3.2 Protocol treatment regimens and post-study therapy

The aromatase inhibitor LET has been suggested to be useful as neoadjuvant endocrine therapy for hormone receptor-positive (especially ER-positive), postmenopausal breast cancer.
Eiermann et al.[16] reported a randomized double-blind controlled study of 4-month neoadjuvant therapy with LET versus tamoxifen in 337 postmenopausal women with primary breast cancer not amenable to breast-preserving surgery. Objective tumor response was achieved in a significantly higher percentage of patients treated with LET than with tamoxifen when assessed by palpation (55% vs. 36%; P<0.001), by ultrasonography (35% vs. 25%; P=0.042) and by mammography (34% vs. 16%; P<0.001). A significantly higher percentage of patients treated with LET than those treated with tamoxifen (45% vs. 35%; P=0.022) subsequently underwent breast-preserving surgery. In a randomized double-blind controlled study, Ellis et al.[17] compared 4-month neoadjuvant treatment with LET and tamoxifen in patients with ER-positive and ErbB-1/ErbB-2 positive breast cancer. They reported that objective response confirmed by direct measurement was obtained in a higher percentage of patients treated with LET than with tamoxifen (60% vs. 41%; P=0.004). Both studies only assessed 4 months' treatment with LET, while Paepke et al.[18] compared the outcomes of 4-month and 8-month neoadjuvant LET therapy. They found a much higher response rate with 8-month than with 4-month treatment (90% vs. 57%). Based on this finding, LET is to be administered for at least 24 weeks (up to 28 weeks) in this study as the neoadjuvant protocol treatment.
LET is included in the standard armament for adjuvant endocrine therapy for postmenopausal hormone receptor-positive breast cancer (see Section 2.2 for the outcomes of adjuvant therapy with LET). No standard regimens have been established as postoperative adjuvant chemotherapy for postmenopausal hormone-sensitive breast cancer. Therefore, regimens generally accepted as standard adjuvant chemotherapy for breast cancer are to be used as adjuvant chemotherapy in the

CL group in this study, such as CMF, AC/EC, TC, AC (EC) followed by paclitaxel (docetaxel), FEC/FAC, FEC followed by docetaxel, and TAC. Monotherapy with any oral fluoropyrimidine (e.g., TS-1, doxyfluridine, capecitabine, 5-fluorouracil) or taxane is not to be used in the adjuvant setting because these regimens have not been accepted as the standard adjuvant treatments for the study population when this study is planned.
As post-study therapy, extended adjuvant endocrine therapy after completion of 5 years' postoperative treatment with LET is not permitted to minimize between-group variability.

2.4 Study design

2.4.1 Rationales for the study endpoints

There is a better chance of long-term survival for patients with breast cancer than those with other malignancies. Therefore, if overall survival is used as the primary endpoint, the number of events will be too small to test study hypotheses, especially in patients at low risk of recurrence such as those to be included in this study. Since overall survival is also affected by post-recurrence treatment, disease-free (recurrence-free) survival is an appropriate endpoint of this study, which is intended to evaluate the efficacy of neoadjuvant and postoperative endocrine therapy. Furthermore, no published studies of postoperative drug therapy for breast cancer have used overall survival as the primary endpoint. Based on these considerations, disease-free (recurrence-free) survival is to be used as the primary endpoint of this study.

2.4.2 Feasibility of subject recruitment

In January 2007, 216 Japanese centers were sent a preliminary questionnaire about their willingness to participate in this study and how many patients potentially eligible for the study were seen at these centers. As shown in Table 5, 124 centers responded to the questionnaire and reported that a total of 966 patients potentially eligible for the study presented to these centers in the previous year. Therefore, the planned enrollment duration (3 years) is considered sufficient for achieving the sample size target at these centers.

Table 5 Results of the Survey about Feasibility of the Study (Conducted from December 2006 to January 2007 by CSPOR)

	Total	Mean	Minimum	Maximum
No. of patients operated on in the year 2005	9,841	107	0	500
No. of patients with postmenopausal breast cancer	5,522	61	3	260
No. of patients with ER-positive cancer	3,737	41	2	200
No. of patients with node-negative cancer	2,470	27	2	180
No. of patients potentially eligible for the study per year	966	11	0	70

2.4.3 Rationale for the stratification factors for randomization

Regarding the known prognostic factors mentioned in Section 2.1.2, patients have to have HER2-negative, ER-positive, and node-negative breast cancer to be included in this study. Patients postoperatively proven to be node-positive are also to be randomized if they have responded (CR or PR) to neoadjuvant therapy and if the number of nodes involved is not more than 3. Therefore, lymph node status is to be used as a stratification factor. In addition, degree

of response to neoadjuvant LET (which may affect survival outcome), PgR (another hormone receptor) status, age (which roughly determines the estrogen level in the circulation), and study center are to be used as the stratification factors.

2.5 Summary of expected benefits and foreseeable risks of participating in this study

All participants in this study will receive the neoadjuvant protocol treatment (LET), and those clinically responding to the neoadjuvant therapy (excluding those whose response is rated as "PD") will then be randomized to receive either of the two postoperative protocol treatments. Thus, the overall outcome of the entire scheme of treatments will be evaluated in the study population. Expected benefits of the neoadjuvant protocol treatment include its potential down-staging effect and the known response to neoadjuvant endocrine therapy, which may provide a hint for optimal postoperative therapy. Currently, there is no decisive evidence that favors either of the two postoperative protocol treatments. Except for the randomization procedure, the postoperative protocol treatments are basically the same as treatment options available in practice and their expected effects and safety would be the same as those expected outside the study.

All medical expenses during participation in this study, including those of medication and laboratory tests, will be paid by the participating patients and/or their health insurances. Therefore, treatment in this study will not bring about any economic benefit or inconvenience to patients compared to treatment in practice. The examinations and tests planned during this study are the same as those routinely performed in practice so that study participation will not increase the economic burden upon patients. However, some of the participants will be requested to periodically fill in questionnaires for HRQOL and pharmacoeconomic assessments, which may be an additional burden upon the patients.

During participation in the study, patients will periodically undergo physical examinations and laboratory tests and will be given the opportunity to freely consult with their physicians or the CRC about their illness and treatment. Overall, patients participating in the study will receive more attentive care for their illnesses, which may be a benefit to the patients.

2.6 Significance of this study

In patients with hormone receptor-positive breast cancer, pathological complete response (pCR), a positive prognostic factor, can hardly be obtained with neoadjuvant endocrine therapy. However, pCR is also difficult to achieve in this population even with intensive neoadjuvant chemotherapy.[19,20] Therefore, it is not meaningful to make efforts to devise neoadjuvant therapy which can achieve a higher rate of pCR in this population.

Intensive neoadjuvant chemotherapy is often intolerable for elderly patients. Moreover, large overseas clinical studies have shown a smaller survival benefit of postoperative adjuvant chemotherapy than endocrine therapy in patients with hormone receptor-positive breast cancer at low risk of recurrence.[21] Furthermore, available data on response of hormone receptor-positive breast cancer to endocrine therapy indicate that not all patients in this population can benefit from postoperative endocrine therapy. In the absence of macroscopic lesions, it is difficult to identify postoperatively a subgroup of patients who are likely or unlikely to benefit from postoperative endocrine therapy.

Based on these considerations, the role of neoadjuvant therapy for postmenopausal, hormone receptor-positive breast cancer is not to increase the rate of pCR but to obtain tumor response data that will help determine optimal postoperative drug therapy, i.e., decide to omit

unnecessary or ineffective postoperative treatments. Neoadjuvant endocrine therapy for postmenopausal, endocrine-responsive, intermediate-risk breast cancer will allow prior identification of a subgroup of patients more likely to benefit from postoperative endocrine therapy. For patients responding to neoadjuvant endocrine therapy, the same endocrine therapy may be sufficient as postoperative adjuvant therapy and postoperative adjuvant chemotherapy may not always be necessary. Such screening for optimizing postoperative adjuvant therapy will provide a treatment strategy of great clinical significance.

Gene screening kits like Oncotype DX® and MammaPrint® have been used overseas to make attempts to prospectively identify patients likely or unlikely to benefit from postoperative chemotherapy. TAILORx Study conducted by a North American group is using Oncotype DX®,[22] while MINDACT Study conducted by an European group is using MammaPrint®.[23] These gene screening kits are very expensive, which may prevent their health insurance coverage and wide acceptance in practice in Japan. Therefore, response to neoadjuvant endocrine therapy may become a very useful tool in practice if it can help determine postoperative drug therapy.

2.7 Significance of HRQOL assessments

For the evaluation of total benefit of cancer care to patients, it is very important to assess patient-reported outcomes such as HRQOL in addition to survival, an objective outcome measure. Recent large clinical studies of postoperative chemotherapy for breast cancer have commonly used a QOL measure as a primary or secondary endpoint.[24-27] Several large clinical studies conducted in the US/Europe have evaluated the benefit of postoperative therapy with selective aromatase inhibitors for breast cancer, while few studies have addressed neoadjuvant endocrine therapy. Since QOL assessment may involve some cross-cultural issues, QOL assessment in Japanese breast cancer patients in this study will have clinical significance.

2.8 Significance of pharmacoeconomic (cost-effectiveness) assessments

Pharmacoeconomic assessments take account of all related costs, not only the cost of medication to treat the illness but also the cost of medication to relieve any adverse effect and the cost of patient attendance to outpatient clinics. Comparison of quality-adjusted life year (QALY) (which measures the cost of survival with good QOL for 1 year) and calculation of incremental cost per QALY (the ratio of cost per QALY in the study group relative to the control group) lead to many-sided assessment of DFS, QOL and pharmacoeconomic variables, which will make a great contribution to the choice of treatments in cancer care and will help discuss the social significance of individual treatments.

2.9 Comprehensive Support Project for Oncology Research (CSPOR)

In 2000, the public interest incorporated foundation Public Health Research Foundation (PHRF; formerly an incorporated foundation) launched a project for supporting development of breast cancer treatments, including sociopsychological interventions, to increase QALY in breast cancer patients. The PHRF was founded in 1984 and certified as a designated public interest corporation by the Ministry of Health, Labour and Welfare. The missions of this corporation are to promote studies of the effects of stress on human mental and physical health, to facilitate use of the findings from such studies for disease prevention and health promotion, and thus to contribute to the maintenance and improvement of public health. The prevalence of breast cancer in the Japanese has been increasing with changes of their lifestyles, while patients are

becoming more aware of the importance of their QOL. New insights into the biological characteristics of cancer have been successfully translated into development of novel agents effective for cancer treatment. Investigator-initiated clinical studies which also stress QOL assessments must be conducted to establish evidence-based standard cancer treatments and to facilitate more individualized treatments that take account of each patient's QOL. However, the infrastructure for conducting a large multicenter study is still fragile in Japan. Thus, for the ultimate goals of improving the quality of cancer research and the QOL of cancer patients in Japan, the CSPOR is going to:

1) Plan and execute investigator-initiated cancer clinical studies
2) Study QOL of cancer patients
3) Educate clinical research coordinators (CRCs)
4) Provide information to breast cancer patients, physicians/investigators, and CRCs through the internet.

This study will be conducted under the sponsorship of the CSPOR.

2.10 National Surgical Adjuvant Study of Breast Cancer (N-SAS BC)

The N-SAS BC is a study group which belongs to the Group for Postmarketing Study of Anticancer Agents (led by Dr. Kaoru Abe, the General Director of the National Cancer Center [as of 1993]), which was organized in fiscal 1993 under the "Pharmacoepidemiological Methodology Research Project" sponsored by the Ministry of Health and Welfare (currently Ministry of Health, Labour and Welfare). In the first multicenter study conducted by the N-SAS BC (N-SAS BC 01), 732 patients were enrolled at 45 Japanese centers from October 1996 to March 2001. This was followed by the N-SAS BC 02 Study (conducted from December 2001), 03 Study (conducted from November 2002), 04 Study (conducted from September 2003), and 05 Study (initiated in 2007). This study (N-SAS BC 06) will also be a nationwide multicenter study conducted by the N-SAS BC and will make the best use of the knowledge, experience, and know-how obtained in the previous N-SAS BC studies.

3. Criteria and Definitions Used in This Study

3.1 Clinical staging

The General Rules for Clinical and Pathological Recording of Breast Cancer 15th Edition (2004) will be used. These rules accord with the UICC-TNM Classification 6th Edition (2002).

3.1.1 T category: Extent of primary tumor[Note 1)]

		Dimension (diameter, cm)	Adhesion to the chest wall[Note2)]	Skin edema, ulcers, and satellite skin nodules
TX		Primary tumor cannot be assessed		
Tis		Non-invasive cancer or Paget's disease with no tumors.		
T0		No evidence of primary tumor[Notes 3,4)]		
T1[5)]		≤2.0	–	–
T2		2.0< ≤5.0	–	–
T3		5.0<	–	–
T4	a	Any dimension	+	–
	b		–	+
	c		+	+
	d	Inflammatory breast cancer[Note 6)]		

Note 1) : T classification should be based on overall evidence arising from inspection/palpation and imaging.

Note 2) : The chest wall includes the ribs, sternum, intercostal muscles and anterior serratus muscle but does not include the pectoral muscles.

Note 3) : No evidence of primary tumor arises from inspection/palpation or imaging (mammography or ultrasonography).

Note 4) : In the presence of nipple secretion or breast calcification detected by mammography, immediate classification to T0 should be avoided; classification should be deferred until a final pathological diagnosis becomes available, after which the tumor should be decisively classified as Tis, T1mic or anything else.

Note 5) : T1 tumors should be subclassified into T1*a* (≤0.5), T1*b* (0.5<, ≤1.0), and T1*c* (1.0<, ≤2.0).
If the greatest diameter of the largest focus of invasion is not more than 0.1 cm, the tumor should be additionally described as T1mic.

Note 6) : Inflammatory breast cancer is usually not associated with tumors and presents with diffuse skin redness, edema and induration.

Note 7) : If multiple intramammary tumors are present, the greatest of the numbers assigned to the multiple tumors should be adopted.

3.1.2 N category: Absence or presence and extent of regional lymph node metastasis[Note1)]

		Ipsilateral axillary lymph node		Parasternal lymph node[Note 2)]	Ipsilateral infraclavicular lymph node	Ipsilateral supraclavicular lymph node
		Movable	Fixed (to the surrounding tissue or between lymph nodes)			
NX		Regional lymph node status cannot be assessed				
N0		–	–	–	–	–
N1		+	–	–	–	–
N2	a	–	+	–	–	–
	b	–	–	+	–	–
N3	a	+/–	+/–	+/–	+	–
	b	+	or +	+	–	–
	c	+/–	+/–	+/–	+/–	+

Note 1) : N classification should be based on evidence arising from palpation and imaging.
Note 2) : Metastasis to the parasternal lymph node should be considered absent if this lymph node has not been examined.

3.1.3 M category: Absence or presence of metastatic tumor

MX: Metastatic tumor cannot be assessed
M0 : Absence of metastatic tumor
M1 : Presence of metastatic tumor

3.1.4 Staging by TNM Classification

		T0	T1	T2	T3	T4
M0	N0		I	IIA	IIB	IIIB
	N1	IIA	IIA	IIB	IIIA	IIIB
	N2	IIIA	IIIA	IIIA	IIIA	IIIB
	N3	IIIB	IIIB	IIIB	IIIB	IIIB
M1		IV	IV	IV	IV	IV

Stage 0: Tis (non-invasive cancer)
: Not applicable
Stages I-IV: Invasive cancer

Patients with T1c-2 N0 M0 carcinoma out of stage I-IIA disease (in the non-shaded groups in the above figure) are eligible for this study.

3.2 Histological typing

The General Rules for Clinical and Pathological Recording of Breast Cancer 15th Edition (2004) will be used.

1 Noninvasive carcinoma
  1a. Noninvasive ductal carcinoma
  1b. Lobular carcinoma *in situ*
2 Invasive carcinoma
  2a. Invasive ductal carcinoma
    2a 1 Papillotubular carcinoma
    2a 2 Solid-tubular carcinoma
    2a 3 Scirrhous carcinoma
  2b Carcinomas of specific types
    2b 1 Mucinous carcinoma
    2b 2 Medullary carcinoma
    2b 3 Invasive lobular carcinoma
    2b 4 Adenoid cystic carcinoma
    2b 5 Squamous cell carcinoma
    2b 6 Spindle cell carcinoma
    2b 7 Apocrine carcinoma
    2b 8 Carcinoma with cartilaginous and/or osseous metaplasia
    2b 9 Tubular carcinoma
    2b 10 Secretory carcinoma (Juvenile carcinoma)
    2b 11 Miscellaneous
3 Paget’s disease

3.3 Performance status (PS) grading

The Japanese version of the ECOG scale[28] will be used.

Grade	Performance status
0	Fully active, able to carry on all pre-disease performance without restriction.
1	Restricted in physically strenuous activity but ambulatory and able to carry out work of a light or sedentary nature, e.g., light house work, office work.
2	Ambulatory and capable of all selfcare but unable to carry out any work activities. Up and about more than 50% of waking hours.
3	Capable of only limited selfcare, confined to bed or chair more than 50% of waking hours.
4	Completely disabled. Cannot carry on any selfcare. Totally confined to bed or chair.

3.4 Criteria for rating response to the neoadjuvant protocol treatment (neoadjuvant LET)

3.4.1 Criteria for rating clinical response

The clinical response rating criteria in the General Rules for Clinical and Pathological Recording of Breast Cancer 15th Edition, which accord with the RECIST,[29] will be used with the following modifications:
Clinical response should be assessed by mono-dimensional measurement of the target tumor. At all specified times, however, the target tumor should be measured in two dimensions (three dimensions by ultrasonography) and all measurements should be reported.
If sister nodules are present in a segment, the largest nodule should be selected as the target tumor, which should be measured for assessing clinical response.

Response of the target tumor (confirmation of response after 4 weeks is not necessary)

1) Complete response (CR)
   The target tumor has disappeared, or completely undergone tumor-related secondary changes.
2) Partial response (PR)
   The largest diameter of the target tumor has been reduced from baseline by ≥30%.
3) Stable disease (SD)
   The largest diameter of the target tumor has been changed from baseline by >–30% or <+20%.
4) Progressive disease (PD)
   The largest diameter of the target tumor has increased from baseline by ≥20%.

3.4.2 Criteria for rating histological response

The General Rules for Clinical and Pathological Recording of Breast Cancer 15th Edition (2004) will be used with some modifications:

Grade 0 No response
Cancer cells have undergone no or little treatment-related changes

Grade 1 Fair response
1a) Mild response
Cancer cells have undergone minor changes, regardless of their area, OR
About less than 1/3 cancer cells have undergone major changes.
1b) Moderate response
About 1/3 or more and less than 2/3 cancer cells have undergone major changes.

Grade 2 Good response
About 2/3 or more cancer cells have undergone major changes.

Grade 3 Excellent response
All cancer cells have become necrotic or disappeared, OR have been replaced by granulomatous tissue or fibrotic foci. If all cancer cells have disappeared, there has to be pathological evidence for the presence of cancer before treatment.

These rating criteria may be tabulated as follows:

Extent of changes / Relative area of changes	Minor changes	Major changes
<1/3	1a	1a
≥1/3, <2/3	1a	1b
≥2/3	1a	2

Note 1: Minor changes are defined as minor degenerative changes which may not prevent survival of cancer cells (including cytoplasmic eosinophilic degeneration, vacuolation and nuclear enlargement)

Note 2: Major changes are defined as major degenerative changes which may prevent survival of cancer cells, almost causing their collapse (including lysis, necrosis, and disappearance).

Note 3: Findings in biopsy specimens (obtained by needle biopsy or partial tumorectomy) should not be used to assign a final response rating but should only be recorded for individual specimens.

Note 4: If the histological findings are ambiguous, the grade representing the poorer response should be adopted.

Note 5: Multiple pathological sections should be examined before assigning a rating of Grade 3.

Note 6: Grade 3 is equivalent to pathological CR, a rating used to report response to neoadjuvant therapy.

3.5 Evaluation of tumor recurrence

3.5.1 Definition and classification of recurrence (See Section 1-18 of the General Rules for Clinical and Pathological Recording of Breast Cancer 15th Edition.)

Recurrence is defined as reappearance of histologically (or cytologically) proven breast cancer which has clinically been resolved by any treatment (e.g., surgery, radiotherapy, chemotherapy). Another primary cancer should not be regarded as recurrence. According to the site of recurrence, recurrent breast cancer should be classified as follows:

1) Recurrence in ipsilateral preserved breast
2) Local recurrence in the ipsilateral chest wall
   In this context, the chest wall is defined as the area below the inferior clavicular margin, above the costal arch, lateral to the sterna midline, and medial to the anterior rim of the broadest muscle of back. If recurrence is found on the boundary, the recurrence should be regarded as local.
3) Recurrence in the regional lymph node
4) Distant recurrence

3.5.2 Diagnosis of recurrence

An established diagnosis of recurrence should be based on the following:

1) Recurrence in the ipsilateral preserved breast
   Histological (biopsy) or cytological diagnosis.
2) Local recurrence in the ipsilateral chest wall
   Histological (biopsy) or cytological diagnosis.
3) Recurrence in the regional lymph node
   Histological (biopsy), cytological, or imaging diagnosis.

4) Distant organ metastasis
   i) Contralateral breast
      Cytological or histological (biopsy) diagnosis of breast cancer with clinical or pathological evidence of metastasis. Asynchronous bilateral breast cancer should not be reported as recurrence but as secondary (multiple primary) cancer.
   ii) Distant lymph node
      Histological (biopsy), cytological, or imaging diagnosis.
   iii) Lungs
      Radiographic (e.g., plain chest X-ray, CT) findings consistent with pulmonary metastasis of breast cancer, i.e., multiple nodules.
      When a new solitary nodule appears which cannot be distinguished from primary lung cancer, appropriate procedures for differential diagnose should be followed before confirming metastasis of breast cancer.
      Any primary lung cancer should be reported as “secondary cancer”.
   iv) Bone marrow
      Diagnosis by marrow aspiration.
   v) Bone
      Characteristic radiographic findings (bone lysis or formation), or abnormal bone scintigraphic/MRI findings together with data that rule out other diagnostic possibilities.
   vi) Pleura (pleural effusion)
      Positive cytology or pleural biopsy.
   vii) Meninges
      Cytological diagnosis.
   viii) Ascites (peritoneum) and pericardial effusion
      Cytological or histological (biopsy) diagnosis.
   ix) Eyes
      Diagnosis by funduscopy, CT or MRI.
   x) Liver
      Diagnosis by ultrasonography, CT or MRI.
   xi) Skin
      Histological (biopsy) or cytological diagnosis.
   xii) Brain and spine
      Diagnosis by CT or MRI.
   xiii) Other organs
      Cytological or histological diagnosis. OR data that rule out other diagnostic possibilities even with ambiguous cytological or histological findings.

5) Diagnosis of recurrence using tumor markers
   i) Recurrence should not be diagnosed solely based on elevated levels of tumor markers.
   ii) Simultaneous and substantial elevations of multiple tumor markers or a continuous elevation of a single tumor marker over time strongly suggests recurrence. If such an elevation is noted, imaging and other appropriate diagnostic procedures should be performed to confirm recurrence.

3.5.3 Definition of date of recurrence

The first date of collecting a cytological or histological material or performing imaging that confirms recurrence defines the date of recurrence (definition based on the first-evidence principle).

4. Inclusion and Exclusion Criteria (for primary and secondary enrollment)

4.1 Criteria for primary enrollment

4.1.1 Inclusion criteria

Those who meet all of the following requirements will be considered for admission to the first part of the study:

1) Postmenopausal women with histologically diagnosed primary invasive breast cancer. A woman will be considered postmenopausal if she:
   i) Is 60 years old or older,
   ii) Is under 60 years of age and had her last menstrual period more than 1 year ago (and if she has not undergone hysterectomy), and/or
   iii) Has undergone bilateral ovariectomy.

   Even if the above criteria cannot clarify the menopausal status, a woman will be considered postmenopausal if she has postmenopausal plasma levels of FSH (e.g., ≥30 mIU/mL) and estradiol (e.g., <10 pg/mL) designated at each center.

2) T1c-T2, N0, M0 carcinoma according to the TNM classification (See Section 3.1 Clinical staging.)

3) Positivity for estrogen receptor (≥10% cells stained in an immunohistochemical [IHC] assay) in a pretreatment needle biopsy specimen

4) Negativity for HER2 (2+ or worse rating on the IHC assay or a negative FISH assay available) in a pretreatment needle biopsy specimen. (Negativity for FISH assay would be acceptable, if the assay has been already obtained.)

5) Age under 76 years at primary enrollment

6) PS graded as 0 or 1
   (According to the ECOG criteria; see Section 3.3 Performance status grading.)

7) No previous treatments of breast cancer at primary enrollment

8) Maintenance of adequate organ function as indicated by the following clinical and laboratory data (obtained within 4 weeks before primary enrollment):
   i) WBC : ≥3,000/mm^3
   ii) Platelet count : ≥100,000/mm^3
   iii) GOT/GPT : ≤2.5 times the upper limit of normal (ULN) adopted at the center
   iv) Serum creatinine : ≤1.5 mg/dL
   v) No treatments of ischemic stroke at enrollment
   vi) No history of myocardial infarction or congestive heart failure and absence of concurrent ischemic heart disease and valvular disease requiring any therapeutic intervention

9) Patient's informed consent to the study obtained after providing written information (Appendix A)

4.1.2 Exclusion criteria

Those who have any of the following conditions, even if they meet all of the inclusion criteria, will not be included in the first part of the study:

1) Node-positive carcinoma diagnosed by sentinel lymph node biopsy, performed before primary enrollment
2) Synchronous or asynchronous bilateral breast cancer
3) Multiple tumors located in multiple breast segments
4) Double primary invasive cancer untreated or diagnosed within 5 years after completion of treatment of the previous cancer
5) A history of breast cancer
6) Current treatment with any continuous systemic corticosteroid, any estrogen-containing agent, or any selective estrogen receptor modulator (SERM)
7) Current participation in any other clinical study of breast cancer
8) Any other condition that disqualifies the patient for inclusion in this study in the subinvestigator's opinion

4.2 Criteria for secondary enrollment

4.2.1 Inclusion criteria

Those who meet all of the following requirements will be considered for inclusion in the randomized part of the study:

1) Clinical response to the neoadjuvant protocol treatment (LET therapy) judged as CR, PR or SD (See Section 3.4.1 Clinical response rating criteria.)
2) Completion of any surgical treatment of breast cancer as scheduled in the protocol (See Section 5.6.)
3) The following lymph node status found after axillary lymph node dissection:
   i) Patients with CR or PR : No lymph node metastasis (Node-negative), or metastasis positive (1 to 3 nodes involved)
   ii) Patients with SD: No lymph node metastasis or metastasis positive (1 to 3 nodes involved), and the following criteria are met.
      - Nuclear grade ≤ Grade 2
      - No widespread invasion of the vasculature surrounding the tumor

   However, a patient who does not undergo lymph node dissection because of negative sentinel lymph node biopsy will be considered as "node negative".

4.2.2 Exclusion criteria

Those who have any of the following conditions, even if they meet all of the inclusion criteria, will not enter the randomized part of the study:

1) A positive FISH assay for HER2 (proved after primary enrollment) in a needle biopsy specimen

2) Patient's refusal to enter the postoperative randomized part of the study

5. Enrollment and Treatment Plan

5.1 Definitions of parts of the study

Various parts of the study are defined as follows:

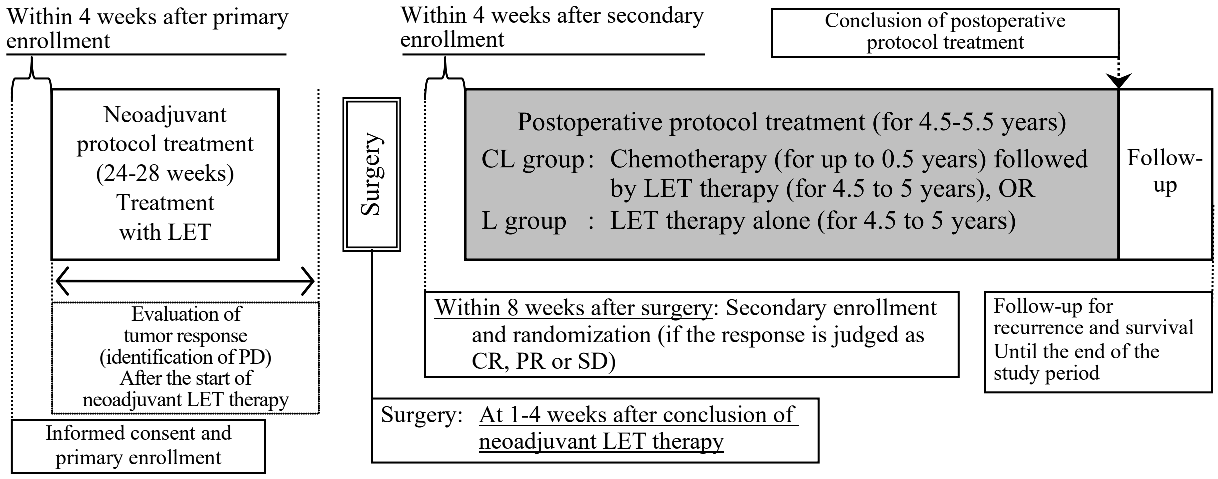

5.2 Primary enrollment procedure

5.2.1 Sending the primary enrollment form

After confirming the eligibility of a patient (that she meets all of the inclusion criteria [see Section 4.1.1] and does not have any of the conditions included in the exclusion criteria [see Section 4.1.2]) through screening procedures (see Section 8.1), the subinvestigator will record all information required (see Section 8.1.1) about the patient in the Primary Enrollment Form (Appendix B) and will send the form by fax to the CSPOR Data Center.

Contact with the CSPOR Data Center
Fax: 03-5298-8536 Phone: 03-3254-8029
Contact hours: From 10:00 to 17:00 on weekdays
(other than holidays, Saturdays and Sundays).

5.2.2 Central registration and communication with the subinvestigator

The CSPOR Data Center will reconfirm the eligibility of the patient according to the information recorded on the Primary Enrollment Form and will then enroll the patient.

1) The patient will not be enrolled if the data recorded in the Primary Enrollment Form are defective.
2) Approved enrollment will never be cancelled; a patient once enrolled will not be excluded from the database.
3) For a patient enrolled more than once, the data used for the earliest enrollment (subject number) will be considered valid.
4) Erroneous or repeated enrollment noticed at each center should be reported as soon as possible to the CSPOR Data Center as such a case requires specific processing on the database.

After approving enrollment, the CSPOR Data Center will send a written confirmation of primary enrollment (Primary Enrollment Confirmation Letter; Appendix B) by fax to the subinvestigator through the fax phone number recorded on the Primary Enrollment Form. This will be followed by a postal mail confirmation.
The CSPOR Data Center will register every eligible patient on the database via the Electric Data Capture (EDC) system.

5.2.3 Withdrawal before initiation of the neoadjuvant protocol treatment

The Study Initiation Report and the Progress Report will be collected for every patient once enrolled even if she is withdrawn before initiating the neoadjuvant protocol treatment. For a patient withdrawn before initiating the neoadjuvant protocol treatment at her request (who withdraws consent or decides to discontinue attendance to the center), only the backgrounds to and the status at withdrawal will be investigated and subsequent monitoring will not be performed.

5.3 Prohibited concomitant treatments after primary enrollment

The treatments listed below are not permitted after primary enrollment. No other restrictions or recommendations will be made about concomitant treatments during the study. Patients may be treated for concomitant illnesses (e.g., hyperlipidemia, hypertension, and cardiac disease) and adverse events at the discretion of the subinvestigator.

1) Non-protocol anticancer treatments (e.g., chemotherapy, endocrine therapy, antibody therapy, surgery, and radiotherapy)
2) Hormone replacement therapy
3) Treatment with selective estrogen receptor modulators (SERMs) (e.g., raloxifene).
   Patients can start to receive any antiosteoporotic treatment such as bisphosphonates and active vitamin D derivatives where appropriate.

5.4 Initiation of the neoadjuvant protocol treatment

After checking the Primary Enrollment Confirmation Letter received, the subinvestigator will initiate the neoadjuvant protocol treatment to the patient within 4 weeks after the date of primary enrollment. The subinvestigator or any other person assisting with the study (e.g., CRC) will submit a written report of initiating the neoadjuvant protocol treatment/study (Neoadjuvant Protocol Treatment/Study Initiation Report; Appendix B) to the CSPOR Data Center via the EDC system. The neoadjuvant protocol treatment, designated below, will be given before surgery. If this treatment could not be initiated as specified in the protocol, the reason for failure will be reported in writing (Study Initiation Report; Appendix B).

Oral once-daily treatment with LET at 2.5 mg/day for 24-28 weeks
A patient receiving LET for at least 24 weeks before surgery will be regarded as completing the neoadjuvant protocol treatment. LET will not be administered for more than 28 weeks.

5.4.1 Evaluation of clinical response to the neoadjuvant protocol treatment (neoadjuvant LET therapy)

Response to the neoadjuvant protocol treatment must be evaluated at 1, 2, and 4 months after the start of the treatment as well as at conclusion of the treatment (at 6 months) (see Section 8.9.1). To evaluate clinical response (see Section 3.4.1), inspection/palpation and ultrasonography must be performed at each specified time, and MRI or CT must also be performed at conclusion of adjuvant LET therapy.
Within each patient, the imaging modality used to evaluate clinical response will be the same as that used at screening for primary enrollment. Before and after treatment, ultrasonographic measurements will be made by a single observer using standardized procedures. Clinical response will be evaluated by mono-dimensional measurement of the target tumor. At all specified times, however, the target tumor will be measured in two dimensions (three dimensions by ultrasonography) and all measurements will be reported.
If sister nodules are present in a segment, the largest nodule will be selected as the target tumor, which will be measured for evaluating clinical response.

5.4.2 Identification of PD during the neoadjuvant protocol treatment

For identifying PD during the neoadjuvant protocol treatment, inspection/palpation and ultrasonography must be performed and MRI or CT should desirably be performed. Within each patient, the imaging modality used for evaluating clinical response will be the same as that used at screening for primary enrollment. Before and after treatment, ultrasonographic measurements will be made by a single observer using standardized procedures. Clinical response will be evaluated by mono-dimensional measurement of the target tumor. At all specified times, however, the target tumor will be measured in two dimensions (three dimensions by ultrasonography) and all measurements will be reported. If PD is identified, LET therapy will be promptly discontinued (see Section 5.5) and the subsequent follow-up will follow the procedure specified in Section 9 (Plan for Follow-up of Patients with PD).

5.5 Status of conclusion of the neoadjuvant protocol treatment

The status of conclusion of the neoadjuvant protocol treatment will be classified as follows:

1) Completion (neoadjuvant LET therapy for at least 24 weeks)
2) Termination due to PD
3) Termination due to death; the date and cause of death will be reported. A patient who has discontinued the neoadjuvant protocol treatment for any other reason before her death will not be classified to this category.
4) Termination by the subinvestigator due to any adverse event
5) Termination at the patient’s request for any reason related to an adverse event
6) Termination at the patient’s request for any reason unrelated to an adverse event
   Only patients discontinued for any reason definitely unrelated to any adverse event will be classified to this category.
7) Termination due to patient’s transfer to any other institution
8) Termination for any other reason (before completing 24 weeks of the neoadjuvant protocol treatment); the reason should be reported.

5.6 Surgery

Surgery of breast cancer will be performed at 1-4 weeks after conclusion of the neoadjuvant protocol treatment (LET therapy) (there should be a drug-free period of at least 1 week before surgery). The surgical procedure and other conditions will be chosen by the subinvestigator.

5.7 Measures for failure of secondary enrollment

Patients who do not satisfy the eligibility criteria for secondary enrollment (see Section 4.2) must not be included in the randomized part of the study.
For each such patient, the reason for failure of secondary enrollment will be recorded in the Secondary Enrollment Form (Appendix B), which will then be sent by fax to the CSPOR Data Center. All such patients, like patients with PD, will be followed up for recurrence, survival and HRQOL whenever possible until the end of the study period (see Section 9: Plan for follow-up of patients with PD). Patients found to have recurrence during follow-up will then be followed up for survival only until the end of the study period.
The date of and findings from each follow-up will be reported using the Follow-up Report Form (Appendix B). When follow-up is closed, the last date of follow-up, the reasons for closing follow-up, and the findings from follow-ups will be reported.

5.8 Secondary enrollment

5.8.1 Sending the secondary enrollment form

The subinvestigator will enroll each eligible patient for the randomized part of the study within 8 weeks after surgery. After confirming the eligibility of a patient for the randomized part of the study (that she meets all of the inclusion criteria [see Section 4.2.1] and does not have any of the conditions included in the exclusion criteria [see Section 4.2.2]), the subinvestigator will record all information required (see Section 8.4.1) about the patient in the Secondary Enrollment Form (Appendix B) and will send the form by fax to the CSPOR Data Center.

Contact with the CSPOR Data Center
Fax: 03-5298-8536 Phone: 03-3254-8029
Contact hours: From 10:00 to 17:00 on weekdays
(other than holidays, Saturdays and Sundays).

5.8.2 Central registration

The CSPOR Data Center will reconfirm the eligibility of the patient according to the information recorded on the Secondary Enrollment Form and will then enroll the patient.

1) The patient will not be enrolled if the data recorded in the Secondary Enrollment Form are defective.
2) Approved enrollment will never be cancelled; a patient once enrolled will not be excluded from the database.
3) For a patient enrolled more than once, the data used for the earliest enrollment (randomized group) will be considered valid.
4) Erroneous or repeated enrollment noticed at each center will be reported as soon as possible to the CSPOR Data Center as such a case requires specific processing on the database.

5.8.3 Randomization and stratification factors for randomization

Patients enrolled will be randomized to either the CL group (chemotherapy followed by LET) or the L group (LET therapy alone) at an approximate ratio of 1:1 by dynamic allocation with stratification by the following factors:

1) Response to neoadjuvant LET therapy (CR or PR vs. SD)
2) PgR status at primary enrollment (positive vs. negative)
3) Pathological node status (positive vs. negative)
4) Age at primary enrollment (<60 vs. ≥60 years)
5) Study center
   The randomization algorithm will be determined by the study statistician.

5.8.4 Communication with the subinvestigator

The CSPOR Data Center will send a written confirmation of secondary enrollment (Secondary Enrollment Confirmation Letter [Appendix B]; which includes the assigned treatment for an eligible patient) by fax to the subinvestigator through the fax phone number reported on the Secondary Enrollment Form. This will be followed by a postal mail confirmation.

5.8.5 Withdrawal before initiation of the postoperative protocol treatment

The Postoperative Protocol Treatment Initiation Report and the Progress Report (Appendix B) will be collected for every patient once enrolled even if she is withdrawn before initiating the postoperative protocol treatment. For a patient withdrawn before initiating the postoperative protocol treatment at her request (who withdraws consent or decides to discontinue attendance at the center), only the status at withdrawal will be investigated and subsequent monitoring will not be performed.

5.9 Concomitant treatments after secondary enrollment

Concomitant treatments after secondary enrollment will follow the rules below. No other restrictions or recommendations will be made about concomitant treatments during the study. Patients may be treated for concomitant illnesses (e.g., hyperlipidemia, hypertension, and cardiac disease) and adverse events at the discretion of the subinvestigator.

5.9.1 Postoperative radiotherapy

Radiation toward the residual breast following breast-preserving surgery or toward the chest wall/supraclavicular lymph nodes following mastectomy in node-positive patients will be performed at the following timing:

1) CL group : Performed simultaneously with LET therapy following completion of chemotherapy.
2) L group : Initiated at the same time as LET therapy.

5.9.2 Prohibited concomitant treatments

1) Non-protocol anticancer treatments
2) Hormone replacement therapy
3) Treatment with SERMs (e.g., raloxifene)
   Patients can start to receive any antiosteoporotic treatment such as bisphosphonates and active vitamin D derivatives where appropriate.

5.10 Initiation of the postoperative protocol treatment

After checking the Secondary Enrollment Confirmation Letter received and the assigned treatment, the subinvestigator will initiate the postoperative protocol treatment to the patient within 4 weeks after the date of secondary enrollment. The subinvestigator or any other person assisting with the study (e.g., CRC) will submit a written report of initiating the postoperative protocol treatment (Postoperative Protocol Treatment Initiation Report; Appendix B) to the CSPOR Data Center via the EDC system.
At secondary enrollment, two postoperative protocol treatments, as designated below, will be randomly assigned to individual patients. If the assigned treatment could not be initiated as specified in the protocol, the reason for failure will be reported in writing (Postoperative Protocol Treatment Initiation Report).

CL group (chemotherapy then LET): Chemotherapy (see Section 5.10.1) followed by LET therapy (for 4.5-5 years)
L group (LET alone) : LET therapy* (for 4.5-5 years)

A patient receiving LET for at least 4.5 years postoperatively will be regarded as completing the postoperative protocol treatment. LET will not be administered for more than 5 years postoperatively.

5.10.1 Postoperative chemotherapy (CL group only)

The regimen(s) used for postoperative chemotherapy in patients allocated to the CL group will be selected from those listed below.

1) Postoperative LET therapy will not be initiated before completion of postoperative chemotherapy.
2) Monotherapy with any oral anticancer agent will not be permitted as postoperative chemotherapy.
3) All patients enrolled at a center will be treated with an identical chemotherapeutic regimen, whenever possible.
4) Any other regimen than listed below may be used only if its use has previously been approved by the Executive Committee.
   i) If the regimen may become a new standard treatment, the protocol will be revised to permit its use.
   ii) If the regimen is originally devised by the center, the center will inform the CSPOR Administrative Office of the regimen when the center is registered for participation.

* Oral once-daily treatment with LET at 2.5 mg/day

	CPA	MTX	5-FU	DXR (ADR)	EPI	PTX (PAC)	DTX (DOC)
CMF<br>Every 4 weeks for 6 cycles	75-100 mg (p.o.)<br>d 1-14	40 mg/m²<br>d 1&8	500-600 mg/m²<br>d 1&8				
AC<br>Every 3 weeks for 4 cycles	600 mg/m²			60 mg/m²			
EC<br>Every 3 weeks for 4 cycles	600 mg/m²				75-100 mg/m²		
TC<br>Every 3 weeks for 4 cycles	600 mg/m²						60-75 mg/m²
FAC<br>Every 3 weeks for 6 cycles	500 mg/m²		500 mg/m²	50 mg/m²			
FEC<br>Every 3 weeks for 6 cycles	500 mg/m²		500 mg/m²		75-100 mg/m²		
AC-T/EC-T<br>Every 3 weeks for 4 cycles+4 cycles	600 mg/m²			60 mg/m²	(75-100 mg/m²)	175 mg/m²<br>(80-100 mg/m²/w)	(60-100 mg/m²)
FEC-T<br>Every 3 weeks for 4 cycles+4 cycles<br>Every 3 weeks for 3 cycles+3 cycles	500 mg/m²		500 mg/m²		100 mg/m²	(175 mg/m²)<br>(80-100 mg/m²/w)	60-100 mg/m²
TAC<br>Every 3 weeks for 6 cycles	500 mg/m²			50 mg/m²			60-75 mg/m²

5.11 Status of conclusion of the postoperative protocol treatment

The status of conclusion of the postoperative protocol treatment will be classified as follows:

1) Completion (postoperative LET therapy for at least 4.5 years)
2) Termination due to recurrence
3) Termination due to death; the date and cause of death will be reported. A patient who has discontinued the postoperative protocol treatment for any other reason before her death will not be classified to this category.
4) Termination by the subinvestigator due to any adverse event
5) Termination at the patient's request for any reason related to an adverse event
6) Termination at the patient's request for any reason unrelated to an adverse event
   Only patients discontinued for any reason definitely unrelated to any adverse event will be classified to this category.
7) Termination due to patient's transfer to any other institution
8) Termination for any other reason (before completing 4.5 years of postoperative LET therapy); the reason should be reported.

5.12 Treatment and follow-up after conclusion of the postoperative protocol treatment

After conclusion of the postoperative protocol treatment, patients will receive no anticancer therapy (e.g., chemotherapy, endocrine therapy, antibody therapy, surgery, and radiotherapy) unless they have recurrence or any other malignancy.
After conclusion of the postoperative protocol treatment, all patients will be followed up until the end of the study period.

5.12.1 Follow-up after conclusion of the postoperative protocol treatment until the end of the study period

In this study, all patients will be followed up for recurrence and survival after conclusion of the postoperative protocol treatment until the end of the study period. Patients found to have recurrence during follow-up will then be followed up for survival only until the end of the study period.
As far as possible, the subinvestigator will examine his/her patients once per year for specified clinical and laboratory monitoring and evaluations (see Section 8.7). If this is not possible, the subinvestigator will follow-up his/her patients by any available method at the center (by phone or postal mail) to confirm at least the recurrence and survival status of each patient.
The date of and findings from each follow-up will be reported using the Follow-up Report Form (Appendix B). When follow-up is closed, the last date of follow-up, the reasons for closing follow-up, and the findings from follow-ups will be reported.

6. Drugs Used

Summarized below is the profile of LET, which is based on the prescribing information for the drug. Refer to the prescribing information (Appendix D) for LET as well as chemotherapeutic agents used in this study. The information on all drugs used in this study should continually be updated by making reference to the current drug information available from the official website of the Pharmaceuticals and Medical Devices Agency (http://www.info.pmda.go.jp/).

6.1 Letrozole (FEMARA® Tablet 2.5 mg)

Generic name : Letrozole (LET)

Product name : FEMARA Tablet 2.5 mg
(Novartis Pharma K.K./Chugai Pharmaceutical Co., Ltd.)

6.1.1 Contraindications and conditions requiring administration with care

(1) Contraindications
Women of confirmed or potential pregnancy or in lactation.
Patients with a history of allergy to any ingredient of FEMARA Tablet.

(2) Conditions requiring administration with care
Severe hepatic or renal impairment.

6.1.2 Most frequent adverse drug reactions

(1) Clinically significant adverse drug reactions
Thrombosis and embolism (incidence unknown)

(2) Other adverse drug reactions

1) Unknown incidence (reported overseas only)
Increased appetite, weight decrease, depression, anxiety, insomnia, memory impairment, paraesthesia, cataract, eye irritation, blurred vision, tachycardia, angina pectoris, dyspnea, diarrhea, dry skin, urticaria, bone pain, fractures, osteoporosis, pollakiuria, urinary tract infection, vaginal dryness, fever, mucosal dryness, and tumor pain

2) Incidence ≥5%
Increased blood cholesterol, hot flushes, increased GOT, increased GPT, increased ALP, and increased γ-GTP.

3) Incidence between 1% and 5%
Decreased white blood cell count, hypercalcemia, anorexia, weight increase, headache, floating dizziness, hypertension, nausea, vomiting, increased LDH, pruritus, rash, increased sweating, arthralgia, myalgia, protein urine positive, fatigue, malaise, and thirst.

6.1.3 Drug interactions; caution should be exercised when LET is co-administered with the following drugs:

(1) Drugs which inhibit CYP2A6, such as methoxsalen
These drugs inhibit the metabolism of LET, potentially increasing its blood level.

(2) Drugs which inhibit CYP3A4, such as azole antifungals
These drugs inhibit the metabolism of LET, potentially increasing its blood level.

(3) Drugs which induce CYP3A4, such as tamoxifen and rifampicin
These drugs promote the metabolism of LET, potentially decreasing its blood level.
LET inhibits CYP2A6, potentially increasing the blood concentrations of other drugs which are metabolized by CYP2A6.

6.1.4 Metabolism and excretion

LET is metabolized to the pharmacologically inactive carbinol metabolite, which is then glucuronized and excreted primarily via the kidney. *In vitro* experiments using human hepatic microsome have suggested that CYP3A4 and CYP2A6 may primarily be responsible for the biotransformation of LET. A study of the ability of LET to inhibit various CYP isozymes has shown Ki values of 0.12 and 8.5 μmol/L against CYP2A6 and CYP2C19, respectively. In healthy (non-Japanese) postmenopausal women given a single oral dose of LET, 88.2% and 3.8% of the dose were recovered in the urine and feces, respectively, by 336 hours post-dose. The parent compound and β-O-glucuronide of the carbinol metabolite, respectively, accounted for about 6% and 75% of related compounds excreted in the urine.

7. Assessment of Adverse Events

An adverse event is defined as any undesirable symptom/sign (including an abnormal laboratory finding) occurring to a subject after the start of the study treatment, whether related or unrelated to the treatment. If an adverse event occurs, the subinvestigator will promptly take necessary measures (e.g., examinations, treatments of the adverse event, discontinuation of the study treatment) to ensure the subject's safety. In this study, adverse events will be evaluated as follows:

7.1 Evaluation of adverse events

Evaluated in this study will be adverse events occurring to a subject during the neoadjuvant or postoperative protocol treatment whose causal relationship with the protocol treatment cannot completely be ruled out. Recurrence of breast cancer during the study period will be evaluated as worsening of the underlying malignancy and will not be reported as an adverse event.
Each adverse event will be described and graded according to the Japanese (JCOG/JSCO) version of the Common Toxicity Criteria for Adverse Events (CTCAE) Version 3.0 (Appendix F).

7.1.1 Timing of evaluation

In this study, adverse events will be evaluated only during the neoadjuvant and postoperative protocol treatments. Adverse events occurring during the following parts of the study period will not be evaluated:

1) From primary enrollment until the start of the neoadjuvant protocol treatment
2) From conclusion of the neoadjuvant protocol treatment until the start of the postoperative protocol treatment
3) From conclusion of the postoperative protocol treatment onward

7.1.2 Adverse event categories

According to the CTCAE terminology, adverse events will be classified into the categories described below and reported as such in the Progress Report. They will be graded and those classified as "miscellaneous" will be described according to the CTCAE terminology.

1) Adverse events to be evaluated during LET therapy (both in the CL and L groups)

Cardiovascular-general	:	Hypertension
Body as a whole	:	Fatigue (asthenia, lethargy, malaise) and sweating
Dermatologic/Cutaneous	:	Pruritus/itching, rash/exfoliation, rash: acne/acneiform, rash: erythema multiform, rash: hand-foot skin reaction
Endocrine	:	Hot flushes (facial flushing)
Gastrointestinal	:	Anorexia and nausea
Metabolic/Investigations	:	Increased ALT/SGPT, increased AST/SGOT, and increased serum cholesterol (hypercholesterolemia)
Musculoskeletal/soft tissue	:	Fractures, joint function, and osteoporosis
Neurologic	:	Mood change-choice: Depression
Pain	:	Pain-choice: joint, pain-choice: head/headache
Miscellaneous		

2) Adverse events to be evaluated during chemotherapy (in the CL group only)

Allergic/immunologic	:	Allergic reaction/hypersensitivity
Hematologic/bone marrow	:	Hemoglobin, white blood cells, neutrophils/granulocytes, and platelets.
Body as a whole	:	Fever (in the absence of neutropenia)
Dermatologic/cutaneous	:	Alopecia (scalp and entire body) and nail changes
Gastrointestinal	:	Diarrhea, mucositis/stomatitis (physical findings)-choice: oral, mucositis/stomatitis (function/symptom)-choice: oral, taste alteration (taste disturbance), and vomiting
Infections	:	Neutropenic fever (fever of unknown origin with no evidence of infection)
Lymphatic vessels	:	Edema: occipital, edema: limbs, edema: trunk/genital, edema: visceral.
Neurologic	:	Neuropathy: motor, neuropathy: sensory
Pain	:	Pain-choice: muscle
Miscellaneous		

7.2 Known and unknown adverse events

Adverse events listed in the current prescribing information for the drug are defined as known adverse events, while those not listed in the current prescribing information for the drug are defined as unknown adverse events.

8. Clinical and Laboratory Monitoring and Assessments

8.1 Screening for primary enrollment

Prior to primary enrollment, each patient will be screened for eligibility for the study based on information about the items listed below. Laboratory data obtained within 4 weeks and imaging data obtained within 6 weeks before primary enrollment may be used for the screening purpose.

1) Date of diagnosing breast cancer and the surgical procedure planned to be used
2) Clinical stage (See Section 3.1 Clinical staging.)
3) Tumor size, measured bidimensionally by inspection/palpation, tridimensionally by ultrasonography, and bidimensionally by MRI or CT (all 3 modalities mandatory).
4) Axillary node status, assessed by inspection/palpation with or without imaging
5) Bone, liver, and lung screening (to confirm absence of metastasis to these organs) by bone scintigraphy, plain chest X-ray or CT, and abdominal CT or ultrasonography (all 3 modalities mandatory)
6) Pathological examination for 1) histological typing (see Section 3.2 Histological typing) and 2) histological grading (only for invasive carcinoma) (according to the N-SAS BC Criteria for histopathological grade)
7) Hormone receptor (ER and PgR) status: carcinoma with 10% or more positive cells in an IHC assay will be defined as "receptor-positive".
8) HER2 status, assessed by an IHC and/or FISH assay.
9) Whether the patient has undergone sentinel lymph node biopsy.
10) Gestational status and whether the patient has undergone hysterectomy or ovariectomy.
    The blood levels of estradiol and FSH should desirably be measured in a patient aged between 50 and 59 years.
11) Height, body weight, and PS (See Section 3.3 Performance status grading.)
12) Routine laboratory tests to assess the following: WBC, Hb, platelet count, GOT, GPT, creatinine, γ-GTP, ALP, total cholesterol, and triglycerides.
13) Concomitant illnesses and a history of malignancies
14) HRQOL: Investigated at each center before sending the Primary Enrollment Form.

*No survey is required in patients who were newly enrolled after December 15, 2011.

8.1.1 Filling in the Primary Enrollment Form

Based on the results of screening, information on the following will be entered into the Primary Enrollment Form:

1) Date of birth, the scheduled date of initiating preoperative treatments, height and body weight.
2) That the patient meets each of the inclusion criteria (answering yes or no) and the date of obtaining the patient's informed consent.
   Laboratory data obtained at screening (WBC, platelet count, GOT, GPT, and creatinine)
3) That the patient does not have any condition included in the exclusion criteria (answering yes or no).

4) Whether the patient's HRQOL has been assessed.

*No survey is required in patients who were newly enrolled after December 15, 2011.

8.1.2 At the start of the neoadjuvant protocol treatment: Filling in the Study Initiation Report

Based on the results of screening and the status of initiating treatment, information on the following will be entered into the Study Initiation Report:

1) Information on the primary tumor (date of diagnosis, tumor size, methods used to determine tumor size, clinical stage, histological type, and histological grade before the start of the neoadjuvant protocol treatment, HER2 status, whether the patient has undergone sentinel lymph node biopsy, and the surgical procedure planned to be used), the gestational status, PS, and a history of malignancies
2) Results of bone, liver and lung screening by bone scintigraphy, chest X-ray or CT, and abdominal CT or ultrasonography.
3) Date of initiating the protocol treatment, or the reason for failure to initiate the treatment as specified in the protocol, where appropriate.
4) Concomitant illnesses

8.2 During the neoadjuvant protocol treatment

At 1, 2, and 4 months after the start of neoadjuvant LET therapy, each patient will be monitored for the items listed below. As the exceptions, HRQOL will be examined only at 1 and 4 months and pharmacoeconomic assessments will be made only at 4 months after the start of neoadjuvant LET therapy (see Section 8.9.1)*.

1) Tumor size, measured bidimensionally by inspection/palpation (mandatory) with or without MRI/CT, and tridimensionally by ultrasonography (mandatory).
2) Whether the protocol treatment and any prohibited concomitant treatment have been given.
3) Medical interview
4) Routine laboratory tests (as defined above)
5) Adverse events
(Baseline data will be obtained at the start of the neoadjuvant protocol treatment.)
6) *HRQOL: Only at 1 and 4 months after the start of treatment.
7) *Pharmacoeconomic assessments (direct non-medical cost plus indirect cost):
Only at 4 months after the start of treatment.

*No survey is required in patients who were newly enrolled after December 15, 2011.

8.3 At conclusion (termination or completion) of the neoadjuvant protocol treatment

At conclusion of neoadjuvant LET therapy (including termination due to PD judged during the neoadjuvant protocol treatment), each patient will be monitored for the following:

1) Clinical stage at conclusion of the neoadjuvant protocol treatment.
2) Tumor size, measured bidimensionally by inspection/palpation, tridimensionally by ultrasonography, and bidimensionally by MRI or CT (all measurements mandatory).
3) Tumor response and the method of evaluating response
Within each patient, the imaging modality used to evaluate clinical response (see Section 3.4.1) will be the same as that used at screening for primary enrollment. If PD is identified during the neoadjuvant protocol treatment, MRI or CT will be performed on an optional

basis (see Section 5.4.2) and the subsequent follow-up will follow the procedure specified in Section 9 (Plan for Follow-up of Patients with PD).

4) Protocol treatment status (date of concluding the treatment and conclusion status [see Section 5.5], and whether any prohibited concomitant treatment has been given.
5) Medical interview, body weight, and PS
6) Routine laboratory tests (as defined above)
7) Adverse events

8.4 After surgery

8.4.1 At secondary screening: Filling in the Secondary Enrollment Form

1) Date of birth, date of surgery, the scheduled date of initiating postoperative treatment
2) That the patient meets the inclusion criteria with regard to:
   Clinical response to the neoadjuvant protocol treatment
   Whether sentinel lymph node biopsy has been performed or the lymph node status has been examined after lymph node dissection
   Postoperative pathological axillary node status and number of nodes involved
   Histological grade, presence of widespread invasion of the vasculature surrounding the tumor (in case of SD only)
3) That the patient does not have the following condition included in the exclusion criteria:
   A positive FISH assay for HER2 (proved after primary enrollment)

8.4.2 At the start of the postoperative protocol treatment

1) Surgery: Surgical procedure, whether axillary lymph node dissection has been performed, and whether breast reconstruction surgery has been performed (as well as the procedure)
   For a patient who has undergone breast-preserving surgery, the resection margin status (a margin ≥5 mm, a margin <5 mm, or cancer cells exposed) will also be reported.
2) Tumor size (the greatest diameter of the largest focus of invasion) measured in histopathological specimens
3) Pathological response
4) Hormone receptor (ER, PgR) status, if reassessed after surgery by an IHC assay
5) HER2 status, if reassessed after surgery by an IHC or FISH assay
6) Protocol treatment: Date of initiation, the assigned treatment, and the chemotherapeutic regimen (only if assigned to the CL group).
   Concomitant treatments: Whether adjuvant radiation therapy is planned and whether any prohibited concomitant treatment has to be given.
7) Body weight and PS
8) Routine laboratory tests (as defined above)
9) Adverse events (assessed to obtain baseline data)
10) *HRQOL

*No survey is required in patients who were newly enrolled after December 15, 2011.

8.5 During the postoperative protocol treatment

8.5.1 During the first year

At 2, 6, and 9 months after the start of the postoperative protocol treatment, each patient will be monitored for the items listed below. Exceptionally, body weight and PS will be examined only at 6 months; while *HRQOL and *pharmacoeconomic assessments will be made only at 2 months after the start of the postoperative protocol treatment (see Section 8.9.2).

1) Protocol and concomitant treatments: Whether adjuvant radiation therapy has been performed and whether any prohibited concomitant treatment has been given.
2) Medical interview, inspection/palpation, body weight, and PS
3) Routine laboratory tests (as defined above)
4) Adverse events
5) *HRQOL
6) *Pharmacoeconomic assessments (direct non-medical cost + indirect cost)
7) Screening for recurrence/metastasis/secondary cancer
   (See Section 8.8 Monitoring for recurrence.)

*No survey is required in patients who were newly enrolled after December 15, 2011.

8.5.2 During the second and subsequent years of the postoperative protocol treatment

During the second and subsequent years until conclusion of the postoperative protocol treatment, each patient will be monitored every 6 months for the items listed below. As the exceptions, *HRQOL and *pharmacoeconomic assessments will be made only at 1 year after the start of the postoperative protocol treatment (see Section 8.9.2).

1) Protocol and concomitant treatments: Whether adjuvant radiation therapy has been performed and whether any prohibited concomitant treatment has been given.
2) Medical interview, inspection/palpation, body weight, and PS
3) Routine laboratory tests (as defined above)
4) Adverse events
5) *HRQOL
6) *Pharmacoeconomic assessments (direct non-medical cost + indirect cost)
7) Screening for recurrence/metastasis/secondary cancer (see Section 8.8 Monitoring for recurrence); Mammography must be performed every year.

*No survey is required in patients who were newly enrolled after December 15, 2011.

8.6 At conclusion (termination or completion) of the postoperative protocol treatment

At conclusion (termination or completion) of the postoperative protocol treatment, each patient will be monitored for the following:

1) Protocol treatment : Date of concluding the protocol treatment and the conclusion status (see Section 5.11)

Concomitant treatments : Whether adjuvant radiation therapy has been performed and whether any prohibited concomitant treatment has been given.

2) Medical interview, inspection/palpation, body weight, and PS
3) Routine laboratory tests (as defined above)
4) Adverse events
5) Screening for recurrence/metastasis/secondary cancer (see Section 8.8 Monitoring for recurrence): Mammography

8.7 During follow-up:

From conclusion of the postoperative protocol treatment until the end of the study period

1) Whether follow-up has been closed or is ongoing:
   If follow-up is closed, the last date of follow-up and the reason for closing follow-up.
2) Survival status: If the patient has died, the date and cause of the death.
3) Screening for recurrence/metastasis/secondary cancer (see Section 8.8 Monitoring for recurrence)

8.8 Monitoring for recurrence

Inspection/palpation will be performed at every visit, while bilateral mammography will be performed every year. If recurrence is suspected, all examinations necessary for establishing the diagnosis will be performed (see Section 3.5).
Examinations of other organs than the mammary (e.g., chest, abdomen, and bone) will be performed on an optional basis and in accordance with each center's oncology practice policy.

8.9 Schedule of monitoring and assessments

8.9.1 From primary enrollment until conclusion of the neoadjuvant protocol treatment

	Visit	Medical interview and inspection/palpation	Body weight and PS	Routine laboratory tests	Adverse events	Tumor size: Inspection/palpation	Tumor size: Ultrasonography	Tumor size: CT or MRI	HRQOL**	Pharmacoeconomic assessments** (direct non-medical cost + indirect cost)	Pharmacoeconomic assessments** (direct medical cost)
Primary enrollment	1	○	○	○*	○†	○	○*	○*	○‡		↑
After the start of neoadjuvant LET therapy: At 1 month	2	○		○	○	○	○	△	○		
At 2 months	3	○		○	○	○	○	△			
At 3 months	4	△				△	△				
At 4 months	5	○		○	○	○	○	△	○	○	
At 5 months	6	△				△	△				
At conclusion of neoadjuvant LET therapy	7	○	○	○	○	○	○	○			↓
At termination of neoadjuvant LET therapy	-	○	○	○	○	○	○	●			

△ : Performed whenever possible.

● : Performed whenever possible if the protocol treatment is discontinued due to PD identified during the treatment.

* Laboratory data obtained with 4 weeks and imaging data obtained within 6 weeks before primary enrollment may be used.

† Concomitant illnesses present at primary enrollment will be identified.

‡ HRQOL will be assessed after obtaining the patient's informed consent and before sending the Primary Enrollment Form for the patient by fax.

** *No survey is required in patients who were newly enrolled after December 15, 2011.

8.9.2 From secondary enrollment until conclusion of the postoperative protocol treatment

	Visit	Medical interview and inspection/palpation	Body weight and PS	Routine laboratory tests	Adverse events	Mammography	HRQOL**	Pharmacoeconomic assessments** (direct non-medical cost + indirect cost)	Pharmacoeconomic assessments** (direct medical cost)
At secondary enrollment	-						○		
At the start of the postoperative protocol treatment	1	○	○	○	○*				↑
After the start of postoperative protocol treatment: At 2 months ‡	2	○		○	○		○	○	|
At 6 months	3	○	○	○	○				|
At 9 months	4	○		○	○				|
At 12 months (1 year)	5	○	○	○	○	○	○	○	↓
At 1.5 years	6	○	○	○	○				
At 2 years	7	○	○	○	○	○			
At 2.5 years	8	○	○	○	○				
At 3 years	9	○	○	○	○	○			
At 3.5 years	10	○	○	○	○				
At 4 years	11	○	○	○	○	○			
At 4.5 years	12	○	○	○	○				
At 5 years	13	○	○	○	○	○			
At conclusion of the postoperative protocol treatment § (at 5 years to 5.5 years in the CL group)	14	○	○	○	○	○			
At termination of the postoperative protocol treatment	-	○	○	○	○	○			
During follow-up. From conclusion of post-operative LET therapy until the end of the study period.	Every year	○				○			

* Baseline data will be obtained at the start of the postoperative protocol treatment.
‡ Date of visit at 2 months after the start of the postoperative protocol treatment will be determined as follows:
CL group : At the start of the 4th cycle of an every-3-week regimen, at the start of the 3rd cycle of an every-4-week regimen, or at the 9th dose of a weekly regimen.
L group : At 2 months after the start of daily LET therapy.
§ The concluding time depends on the length of the postoperative protocol treatment (varies from 4.5 years to 5.5 years).
** No survey is required in patients who were newly enrolled after December 15, 2011.

9. Plan for Follow-up of Patients with PD

In this study, a patient who has initiated the neoadjuvant protocol treatment and whose response has then been judged as PD will be classified into the PD group and followed up until the end of the study period. Subsequent treatments given to each patient with PD will be determined by the subinvestigator and the results of follow-up will be reported.

9.1 Registration of a patient with PD

If PD is identified in a patient, the subinvestigator will promptly record all information required about the patient in the PD Registration Form (Appendix B) and will send the form by fax to the CSPOR Data Center.

<u>Contact with the CSPOR Data Center</u>
Fax: 03-5298-8536 Phone: 03-3254-8029
Contact hours: From 10:00 to 17:00 on weekdays
(Other than holidays, Saturdays and Sundays)

9.1.1 Information required for registration of a patient with PD

1) Scheduled date of performing surgery

9.2 Monitoring of a patient with PD

Patients with PD will be monitored every year during the study period. As the exceptions, *HRQOL and *pharmacoeconomic assessments will be made at 3 weeks after surgery (before the start of any subsequent treatment) and at 19 months after primary enrollment.
At each specified time, patients in the PD group will be monitored for the items listed below. The date and results of monitoring will be reported in the PD Group Report Form (Appendix B). When follow-up is closed, the last date of follow-up, reasons for closure, and results of follow-ups will be reported.

1) Whether follow-up has been closed or is ongoing:
   When follow-up has been closed, the last date of follow-up and reasons for closing follow-up.
2) Treatments of breast cancer (during the first year) after identification of PD:
   Surgical treatments, preoperative and postoperative drug therapy (whether such therapy has been performed and the regimen), and radiation therapy
3) Treatments given after identification of PD until recurrence or until the end of the study period
4) Screening for recurrence/metastasis/secondary cancer (see Section 9.3 Monitoring for recurrence)
5) Survival status: If the patient has died, the date and cause of the death.
6) *HRQOL and *pharmacoeconomic assessments (direct non-medical cost + indirect cost)

*No survey is required in patients who were newly enrolled after December 15, 2011.

9.3 Monitoring for recurrence

Whenever possible, bilateral mammography will be performed every year. If recurrence is suspected by inspection/palpation (and mammography), other examinations necessary for establishing the diagnosis will be performed (see Section 3.5 Evaluation of tumor recurrence). Examinations of other organs than the mammary (e.g., chest, abdomen, and bone) will be performed on an optional basis and in accordance with each center's oncology practice policy.

9.4 Schedule of monitoring and assessment of patients with PD

	Visit	Recording of post-PD treatments of breast cancer	Monitoring for recurrence	HRQOL**	Pharmacoeconomic assessments** (direct non-medical cost + indirect cost)	Pharmacoeconomic assessments** (direct medical cost)
At registration in the PD group*	-					↑
At 3 weeks after surgery	1			○	○	↕
During the first year after registration in the PD group	2	○	○			↓
(At 19 months after primary enrollment)	…			○	○	
During the second and subsequent years until the end of the study period	Every year		○			

* : Tumor size, how to rate clinical response to the neoadjuvant protocol treatment as PD, and the scheduled date of surgery will be examined.
** No survey is required in patients who were newly enrolled after December 15, 2011.

10. Data Collection

10.1 Submission of data

In this study, most of the data collection procedures will be performed via the EDC system. The subinvestigator or any other person assisting with the study (e.g., CRC) will serially submit data on all patients enrolled in this study to the CSPOR Data Center according to the progress of the study until completion of the study. All manual or electronic data entered by CRCs or any other assistants must be confirmed by the subinvestigator. The report forms and document types used and how and when to send and submit them are summarized in the following table:

No.	Report forms/document types	How and when to send	How and when to submit
1	Center's normal ranges of laboratory values (GOT/GPT)	By postal mail previously to each center	By fax at 1 week before enrollment of the first subject
2	Primary Enrollment Form (Appendix B)	By postal mail previously to each center	By fax at enrollment of each subject
3	Neoadjuvant Protocol Treatment/Study Initiation Report (Appendix B)	(EDC Form)	Via the EDC system within 1 month after the start of treatment
4	Neoadjuvant Protocol Treatment/Progress Report (Appendix B)	(EDC Form)	Via the EDC system within 1 month after each monitoring activity)
5	Secondary Enrollment Form (Appendix B)	By postal mail after primary enrollment	By fax at enrollment of each subject
6	Postoperative Protocol Treatment Initiation Report (Appendix B)	(EDC Form)	Within 1 month after the start of treatment
7	Postoperative Protocol Treatment/Progress Report (Appendix B)	(EDC Form)	Via the EDC system within 1 month after each monitoring activity
8	Follow-up Report (Appendix B) (about recurrence and survival status)	(EDC Form)	Via the EDC system within 1 month after each monitoring activity
9	PD Registration Form (Appendix B)	By postal mail after primary enrollment	By fax at registration of each subject
10	PD Group Report (Appendix B) (about recurrence and survival status)	(EDC Form)	Via the EDC system within 1 month after each monitoring activity.
11	Questionnaire about life and expenses (Appendix C)	By postal mail previously to each selected center	By postal mail within 1 month after each time of assessment
12	Copies of medical practice health insurance claim forms and out-of-hospital prescriptions (hard copies or electronic media).	To be determined for each selected center.	To be determined for each selected center.
13	Expedited Adverse Event Report Form (Appendix B)	By postal mail previously to each center; a center's original form may also be used.	By fax within 72 hours after becoming aware of event onset.

10.2 Data management

According to the standard operating procedures and manuals for managing data, which will be established separately, the CSPOR Data Center will remind subinvestigators of missed submissions, make review and inquiries about data submitted, correct errors in accordance with replies to inquiries, and manage the database. Based on data entries, the CSPOR Data Center will prepare monitoring documents and will determine the data sets to be analyzed in cooperation with the Executive Committee.

11. Reporting of Adverse Events

If any adverse event occurs that has to be reported as defined below, the investigator will report the event to the CSPOR Administrative Office. If any serious adverse event (as defined in the ICH-E2A Guideline) occurs that could potentially be related to the study treatment, the investigator will appropriately assume his/her responsibilities of reporting this to the health authority under the Pharmaceutical Safety Information Reporting System (Item 2 of Article 77-4-2 of the Pharmaceutical Affairs Law) and to the relevant pharmaceutical manufacturer for cooperation with manufacturer's spontaneous adverse drug reaction reporting (Item 1 of Article 77-4-2 of the Pharmaceutical Affairs Law) in accordance with the center's rules.

11.1 Adverse events subject to expedited reporting

Adverse events specified below will be promptly reported using the Expedited Adverse Event Report Form (Appendix B).

1) All deaths that occur during the protocol treatment or within 30 days after the last day of the protocol treatment, whether related or unrelated to the treatment. A death of a patient discontinued from the protocol treatment during post-study therapy is also subject to expedited reporting if this occurs within 30 days after discontinuation of the protocol treatment.
2) Unknown non-hematological toxicities of grade 4 intensity (adverse events not classified in the "hematologic/bone marrow" category according to the CTCAE terminology)
   Unknown adverse events are defined as adverse events not listed in the prescribing information for the drug.

11.2 Adverse events subject to non-expedited reporting

Adverse events to be reported in a non-expedited manner are specified in Section 7.1 (Evaluation of adverse events).

11.3 Investigator's reporting responsibilities and reporting procedures

11.3.1 Expedited reporting

If any adverse event subject to expedited reporting (see Section 11.1) occurs, the subinvestigator will promptly report this to the investigator. If the investigator cannot be contacted, the subinvestigator must assume the investigator's reporting responsibilities in place of the investigator. The investigator must report, in an oral form, every adverse event subject to expedited reporting to the center's head immediately and to the CSPOR Administrative Office within 24 hours. The investigator must also submit a written report of each such event using the Expedited Adverse Event Report Form (or any equivalent center's original form) by fax (phone number: 03-5298-8536) to the CSPOR Data Center **within 72 hours** after becoming aware of the event. The investigator must prepare a more detailed written case report (in any A4 sized format) and submit this together with the initial report by fax to the CSPOR Data Center **within 15 days** after becoming aware of the event.

11.3.2 Non-expedited reporting

The investigator must report all adverse events subject to non-expedited reporting in the earliest Progress Report (Appendix B), which will be submitted at the specified time to the CSPOR Data Center.

11.4 Duties of the CSPOR Administrative Office

11.4.1 Judgment on whether to suspend subject enrollment and whether to promptly report an event to individual centers

When receiving an adverse event report from the investigator, the CSPOR Administrative Office will consult the urgency, significance and influences of the event with the principal investigator or his deputy and will take necessary actions such as temporarily suspending subject enrollment (reporting this action to the CSPOR Data Center and all study centers) and informing all centers of the event.
The CSPOR Administrative Office will strongly recommend the reporting investigator to report the event to the health authority and to the relevant pharmaceutical manufacturer to comply with the pharmaceutical product safety information reporting requirements and to cooperate with the manufacturer's spontaneous adverse drug reaction reporting.

11.4.2 Reporting to the Independent Data Monitoring Committee

If any adverse event reported in an expedited or non-expedited manner from a center is judged as one that must be reported, the principal investigator will report this to the Independent Data Monitoring Committee in writing within 15 days after becoming aware of the event onset. The principal investigator will attach his opinion about the adverse event to the report and will request the committee to review the appropriateness of his decision about measures against the event.

11.5 Review by the Independent Data Monitoring Committee

The Independent Data Monitoring Committee will review the adverse event report submitted by the principal investigator and will make written recommendations to the principal investigator about measures to be taken, including the handling of the specific subject and the suitability of continuing subject enrollment.

12. HRQOL and Pharmacoeconomic Assessments

12.1 HRQOL assessments

12.1.1 Objectives of HRQOL assessments

In this study, health-related quality of life (HRQOL) will be evaluated and compared between groups. Primary domains focused on in the HRQOL assessments include physical health, mental health, social function and daily living function. This study will consist of two parts; in the first part, the subjects will receive neoadjuvant endocrine therapy with LET after primary enrollment. In the second part, the subjects will receive randomized postoperative treatments after secondary enrollment.

(1) Neoadjuvant endocrine therapy
In this study, neoadjuvant endocrine therapy is intended to determine each patient's responsiveness to endocrine therapy and, in this regard, differs from conventional neoadjuvant therapy, which is intended to increase the probability of breast-preserving therapy. In the study population (T1c-T2 N0 breast cancer), for which surgery has long been the primary treatment of choice, it is unknown what impacts neoadjuvant endocrine therapy intended to determine tumor endocrine responsiveness has upon the patient's physical health, mental health, social function, and daily living function.

(2) Postoperative treatment
The objective of this study is to confirm whether response to neoadjuvant endocrine therapy can help determine optimal postoperative drug therapy, i.e., decide to omit unnecessary or ineffective postoperative treatments. After secondary enrollment, patients will be randomized either to the CL group (receiving chemotherapy then LET) or the L group (receiving LET alone). Although it can easily be expected that postoperative treatment with chemotherapy will have greater impacts on HRQOL than non-chemotherapeutic treatment, it is critical in this study to scientifically confirm the difference between the two randomized treatments with respect to physical health, mental health, social function and daily living function. It is also important to compare the economic burdens and relative benefits to the patients between the two groups (see Section 12.2 Pharmacoeconomic assessments).

Thus, the objectives of HRQOL assessments in this study will be:

1) To determine the impacts of neoadjuvant endocrine therapy intended to determine tumor endocrine responsiveness upon HRQOL of patients with T1c-T2 N0 primary breast cancer.

2) To demonstrate the difference in HRQOL of patients screened by responsiveness to neoadjuvant endocrine therapy and pathological tumor characteristics proven in surgical specimens during the two randomized postoperative treatments (with and without chemotherapy).

12.1.2 Assessment tools (questionnaires) used

The following questionnaires (Appendix C) will be used as the HRQOL scales:

1) Functional Assessment of Cancer Therapy (FACT) Endocrine Symptom (ES) and Breast (B) supplemental scales[30-32]: FACT-ES (composed of 18 questions) and FACT-B (composed of 9 questions) are supplemental to FACT-General (G) (the Japanese version composed of 29 questions), a QOL scale generally used in clinical studies of oncology.
2) Hospital Anxiety and Depression Scale (HADS)[33,34]: A scale for rating anxiety and depression, composed of 14 questions.
3) EuroQol 5 Dimension (EQ-5D)[35-37]: A preference-based scale (composed of 5 sets of questions only, excluding VAS ratings) used to quantify utility for pharmacoeconomic assessments.

12.1.3 Schedule of assessments and acceptable time windows

In patients assigned to the CL or L groups, HRQOL will be assessed at the following 6 time points: at primary enrollment (after obtaining informed consent before sending the Primary Enrollment Form by fax), at 1 and 4 months after the start of the neoadjuvant protocol treatment, at secondary enrollment (after sending the Secondary Enrollment Form by fax), and at 2 and 12 months after the start of the postoperative protocol treatment.
Baseline data will be obtained at enrollment. At 1 and 4 months after the start of neoadjuvant endocrine therapy, the impacts of the treatment in early and stable phases on patient's QOL will be assessed. QOL assessments at the subsequent 3 time points will be used to make comparisons between groups and to clarify changes over time.
In patients registered to the PD group, HRQOL will be assessed at 3 weeks after surgery (before initiating any postoperative treatment) and at 19 months after primary enrollment (corresponding to 1 year after the postoperative protocol treatment in the CL or L group).

Deviations from the specified times of HRQOL assessment within the following ranges will be permitted:

1) During the neoadjuvant protocol treatment
   At primary enrollment: After obtaining informed consent before sending the Primary Enrollment Form by fax
   At 1 month after the start of the neoadjuvant protocol treatment : ±2 weeks
   At 4 months after the start of the neoadjuvant protocol treatment : ±2 weeks
2) During the postoperative protocol treatment
   At secondary enrollment: During the 2 weeks after sending the Secondary Enrollment Form by fax
   At 2 months after the start of the postoperative protocol treatment : ±2 weeks
   At 12 months after the start of the postoperative protocol treatment : ±2 months
3) PD group
   At 3 weeks after surgery : ±2 weeks
   At 19 months after primary enrollment : ±2 months

12.1.4 Procedures

(1) Assessment at primary enrollment
QOL assessment at primary enrollment will be performed at each center after obtaining patient's informed consent and before sending the Primary Enrollment Form by fax. Patient's self-rating of QOL in an outpatient clinic should desirably be made in a quiet room other than the doctor's office before examination by her doctor.

<Procedure>
The subinvestigator will previously receive the questionnaire forms by postal mail. The subinvestigator will distribute the questionnaire form to each patient selected for HRQOL assessment and will ask her to fill in the form and to submit the form at a specified time. At the specified time, the subinvestigator will collect the completed forms and send them to the CSPOR Data Center by postal mail. Each patient may directly send the completed form to the Data Center by postal mail if the subinvestigator can check and confirm that the patient has completed and submitted the questionnaire.
If a patient becomes unable to fill in the questionnaire by herself due to worsening of her illness (or for any other reason), a third-party individual such as a CRC or her family member may read out the questions and may fill in the questionnaire in place of the patient. In such cases, this procedure will be documented in the questionnaire. See Appendix C for important notes for third-party rating and measures against missed entries.

(2) Assessments after the start of the neoadjuvant protocol treatment
All questionnaires used after the start of the neoadjuvant protocol treatment will be previously sent after primary enrollment by postal mail from the CSPOR Data Center. The Data Center will inform each study center by fax of individual specified times of assessment. The subsequent procedures will be the same as those for assessment at primary enrollment.

(3) Assessments in patients discontinued from protocol treatments
If the protocol treatment has been discontinued due to disease progression, any new concomitant illness, aggravation of any pre-existing concomitant illness, or any adverse event, QOL assessment will be made whenever the patient is willing to cooperate with the assessment. QOL assessment will also be made whenever possible in a patient transferred to another institution. QOL will not be assessed after a patient has been lost to follow up and discontinued from the protocol treatment. If QOL assessment becomes no longer possible, the reasons (e.g., worsening of an illness, non-medical reasons for patient's refusal) will be investigated. In a patient discontinued from the protocol treatment, QOL assessment will be made at 3 weeks after surgery (where appropriate) and at 19 months after primary enrollment.

12.1.5 Analysis of QOL data

Detailed statistical methods will be specified in the statistical analysis plan, which will be prepared separately. A brief plan of analyses of QOL is described below. Whenever possible, the reliability and validity of QOL questionnaires should be tested and established.

1) To evaluate the impacts of 1 and 4 months of neoadjuvant therapy, analysis of covariance will be performed using a model involving the 1-month or 4-month QOL score as the objective variable and the baseline (pretreatment) score as the covariate.

2) To compare the changes over time after randomization between the two randomized postoperative treatments, serial data analysis will be performed using a linear mixed effect model or a generalized estimating equation involving 2-month and 12-month QOL scores as the objective variables and the baseline score (score before the start of postoperative therapy) as the covariate.
3) Main analyses specified in the statistical analysis plan will be performed by duration of treatment with LET to evaluate the effects of treatment duration on the outcomes in an exploratory manner.

12.1.6 Number of patients selected for QOL assessment and future plan

QOL assessment will be made in about 500 patients out of those initially enrolled in the N-SAS BC 06 Study. When all of the patients in this set have completed 6 months of the neoadjuvant protocol treatment and 1 year of the postoperative protocol treatment, the HRQOL data obtained will be presented at a scientific meeting and published in an English medical journal.

12.2 Pharmacoeconomic assessments

12.2.1 Objectives

Cost-effectiveness (cost-utility) of the two randomized postoperative treatments (chemotherapy followed by LET vs. LET alone) following neoadjuvant endocrine therapy and surgery will be compared in patients with T1c-T2 N0 primary breast cancer screened by endocrine responsiveness and pathological tumor characteristics proven in surgical specimens.

12.2.2 Subjects and duration

1) Participating centers
   Direct medical cost:
      Centers willing to cooperate with pharmacoeconomic assessments and able to make medical practice health insurance claim forms available.
   Direct non-medical cost and indirect cost:
      All study centers (as all patients selected for QOL assessment will be included in the assessment of these cost variables)
2) Number of patients assessed
   Direct medical cost will be investigated in about 50 patients.
3) Duration
   Pharmacoeconomic assessments will be made during the 19 months after primary enrollment.

12.2.3 Methods

1) Analytical method
   Cost-utility analysis (CUA; Note 1) will be performed. If the outcome is comparable between the two groups, cost minimization analysis (CMA) will be performed.
2) Perspective
   Analyses will be performed from societal perspective (Note 2).

3) Outcome variable
   <u>Quality adjusted life year (QALY)</u> will be used as the outcome variable. This will be calculated from DFS (primary endpoint) and QOL score.

4) Cost categories (Note 3)
   Direct and indirect costs will be investigated and calculated from societal perspective.
   Direct costs will include:
      Direct medical cost: Cost of treatments of illnesses and adverse reactions.
   Direct non-medical cost:
      Cost of patient's transport to treatment sites and prostheses relating to illnesses (e.g., supporter, pad, and wig).
   Indirect costs will include:
      Loss of work related to treatments

5) Discount (Note 4)
   Costs and outcomes will be discounted at an annual rate of 3%.[38]

6) Presentation of results (Note 5)
   Cost-effectiveness ratio (CER) and incremental cost-effectiveness ratio (ICER) will be calculated and presented. CER represents the cost of obtaining 1 QALY and will be compared between the two groups. ICER represents the cost of obtaining one additional QALY, calculated in case of a better outcome with chemotherapy than without chemotherapy. If the outcome is comparable between the two groups, ICER will not be calculated and cost minimization analysis will be performed instead to only compare the cost of treatments between groups.

7) Sensitivity analyses
   Sensitivity analysis will be performed for each parameter used to calculate either the cost or outcome by varying the parameter over a specified range. In sensitivity analyses for discount rate, the rate will be varied within the 0-7% range.

12.2.4 Collection of data

(1) Outcomes

<u>DFS</u>

For the patients selected for pharmacoeconomic assessments, data on this primary study endpoint will be extracted from the study database.

<u>QOL (Note 6)</u>

QOL data will be obtained from the QOL questionnaires. For pharmacoeconomic assessments, the QOL scores calculated using the EQ-5D will be used.

(2) Costs

<u>Direct medical cost</u>

Direct medical cost will be based on data from medical practice health insurance claim forms as well as out-of-hospital prescriptions, if issued at the center. Copies of medical practice health insurance claim forms printed and out-of-hospital prescriptions issued will be collected once per year. Electronic data from these data sources may be accepted. The standard procedures for collecting direct medical cost data are described below. For medical practice health insurance claim forms, whether to collect non-electronic or

electronic data would be collected will be determined previously by discussion between each center and the CSPOR Data Center.

1) Request the center to cooperate with pharmacoeconomic assessments (by the Data Center)
2) Reach agreements with the center about the procedures for collecting data
3) Initiate collection of data
4) Copy the medical practice health insurance claim forms and out-of-hospital prescriptions issued for a specific patient, mask the patient identification information (e.g., patient name), write down the subject number assigned to the patient, and send the documents to the Data Center.
5) Review the documents received and enter the data to the database (by the Data Center)

Direct non-medical cost

Questionnaires about direct non-medical cost will be administered to all patients selected for QOL assessments at some of the specified times of QOL assessments, i.e., at 4 months after the start of the neoadjuvant protocol treatment, at 2 months after the start of the postoperative protocol treatment (at 3 weeks after surgery in patients with PD or those discontinued from the protocol treatment), and at 19 months after primary enrollment.

Indirect cost

Indirect cost will be investigated together with direct non-medical cost.

12.2.5 Analysis of data

1) Outcomes
   For each patient selected for pharmacoeconomic assessments, QALY will be calculated from DFS and QOL score.

2) Costs
   Direct medical cost will be classified into the costs of physical examination, medication, injections, other treatments, surgery, laboratory tests, imaging, hospital care, and diet, before entering the data into the database. Costs of individual practices as well as the total medical cost will be calculated.
   Direct non-medical cost and indirect cost will be calculated per patient by treatment regimens after entering the data into the database.

3) Statistical analyses
   Detailed statistical methods and procedures will be specified in the statistical analysis plan, which will be prepared separately.

Note 1: CUA

CMA, cost-effectiveness analysis (CEA), CUA, and cost-benefit analysis (CBA) are among the most commonly used analyses for pharmacoeconomic assessments. All these analyses are classified as full economic evaluations that compare multiple treatments and investigate the efficiency of treatments on the basis of both cost and outcome data.[39]

CMA is used when an outcome is comparable among the treatments to be compared; it only compares the costs and concludes a less costive treatment as more efficient. CEA is most commonly used and compares the cost of achieving one unit of a predefined primary outcome measure. The life year gained is most often used as the primary outcome measure. CUA uses

life year gained and the utility value that measures the QOL during the year(s) as the outcome measures, and compares the cost of achieving one unit of the utility value. QALY is most often used as the unit of utility. In calculating QALY, the QOL is rated using a scale from "0" indicating death to "1" indicating perfect health. Thus, 1 QALY means 1 year of survival in perfect health. CBA converts all impacts of treatments into benefit, i.e., calculates the benefit per cost and compares the net benefit, which is calculated by subtracting the cost from the gross benefit.

Note 2: Pharmacoeconomic perspective
It is important to clearly define the perspective of a pharmacoeconomic study. Although an improvement of patient's health and QOL is universally the target outcome, costs in different ranges are taken into consideration from different perspectives. Pharmacoeconomic studies can be performed from patient, medical practitioner, health insurance, or governor perspective. From patient perspective, costs include direct payments to medical practitioners and costs of transport to treatment sites. From medical practitioner perspective, all (e.g., personnel, material) expenses paid for providing care to the illness are taken into consideration. From health insurance perspective, the focus of attention lies on health insurance payments to medical practitioners. Societal perspective encompasses all these perspectives relevant to the target illness.

Note 3: Classification of costs
For pharmacoeconomic studies, costs may be classified in several ways. Here, direct and indirect costs are explained. Direct costs mean the expenses paid to receive treatments of the illness. What expenses should be included in direct cost depends on the pharmacoeconomic perspective. In general, direct cost falls into direct medical cost (expenses paid to medical practitioners and pharmacies) and direct non-medical cost (other expenses). Indirect cost represents an opportunity cost of lost work, i.e., failure to make activities due to an illness and/or treatments of an illness. Indirect cost is not associated with any actual payment but is important for a pharmacoeconomic study from the viewpoint of lost resources.

Note 4: Discount
Discount converts costs arising over multiple years to a value at any specified time. People generally have high time preference, preferring current benefit to future benefit. Therefore, future costs need discount to be converted to current costs. Several values have been proposed as the optimal discount rate. Whether outcomes should be discounted remains controversial, although most have agreed to apply the same discount rate to outcomes as that applied to costs.

Note 5: CER and ICER
The results of pharmacoeconomic assessment are often expressed as the cost of achieving one unit of the outcome, which is referred to as CER (or cost utility ratio, cost benefit ratio according to the analysis used). These ratios are calculated by dividing the cost of the treatment by its effect.

The CER for treatment A is calculated as:

$$CER = \frac{cost(A)}{effectiveness(A)}$$

When treatment A is compared to treatment B, the ICER of treatment A relative to treatment B (the standard or less expensive treatment) is calculated as:

$$ICER = \frac{cost(A) - cost(B)}{effectiveness(A) - effectivenss(B)}$$

ICER represents the cost of achieving one additional unit of an outcome with the study treatment compared with the control treatment.

Note 6: QOL rating for pharmacoeconomic assessments
In CUA, QOL is rated using a scale from “0” indicating death to “1” indicating perfect health. QOL may be directly estimated on a visual analog scale, or by the time-trade-off or standard gamble technique. Indirect rating of QOL may use global health profile instruments designed to provide a single overall QOL score, such as EQ-5D (an instrument with a 5-dimension/3-level scale) and HUI (an 8-dimension instrument).

13. Central Pathological Review

13.1 Procedures and items

In this study, central pathologists will review pathological sections to determine the pathological response to neoadjuvant endocrine therapy at surgery, the hormone receptor (ER and PgR) status at primary enrollment, changes in ER/PgR, HER2 expression status, and Ki67 expression status. Central pathological characterization will be based on decision by a majority of the Central Pathological Evaluation Committee. If decision by majority is difficult, the Committee will have a discussion to reach an agreement. Changes in ER and PgR status will be determined using Allred Scores and J Score. Changes in HER2 expression status will be determined using scores on IHC specimens (scores 0-3). The proportion of Ki67-positive cells (Ki labeling index) will be determined by changes in the expression of Ki67 protein on immunostaining. Final decisions will be made by majority or discussion among the Central Pathological Evaluation Committee. Each center will follow the rules and procedures established by the center in submitting pathological sections for central review.

13.2 Preparation of pathological specimens

A formalin-fixed, paraffin-embedded block of a pretreatment needle biopsy specimen or surgical specimen will be sliced into 4-μm thick sections, which will then be mounted on MAS-GP glass slides.

13.3 Pathological sections to be collected

Unstained and HE-stained specimens during pretreatment needle biopsy and surgery will be collected in all patients, except those judged as PD for clinical response rating after neoadjuvant therapy with letrozole at each center.
Each section collected from each center will be labeled with the least identification information that links the section with the subject number. More concretely, the attached document should only bear the center name, the subject number, and center pathological number; the patient name or initials or her medical record number must not appear on the section or the attached document.

13.3.1 Sections for determining ER, PgR, and HER2 expression status

1) Five unstained sections of a needle biopsy specimen
2) Five unstained sections during surgery (specimens including representative sections of residual tumor)

13.3.2 Sections for evaluating histological response

1) One HE-stained section of a needle biopsy specimen
2) One HE-stained section during surgery (specimens including representative sections of residual tumor)

13.4 Procedures for transporting pathological sections and an institution to receive sections

When central pathological review is to be made, the CSPOR Administrative Office will inform each center and ask the center to send pathological sections to be collected. More detailed

procedures will be specified in the Written Procedures for Central Pathological Evaluations, which will be established separately.

13.5 Return of pathological sections

After central pathological review, the pathological sections collected from a center will be returned to the center. However, sections stained after collection from a center will not be returned to the center; they will be disposed of by the CSPOR Administrative Office after completion of the study when their retention becomes no longer necessary.

13.6 Use of specimens in other studies

The patient's informed consent will again be needed if all or some of the specimens submitted are used for related studies of this study (translational research) other than the central pathological evaluation. The details will be specified in the protocol of each related study (translational research). Even in such a case, the internal rules of each center or the judgment of the ethical committee should be followed.

14. Central Imaging Evaluation

14.1 Purpose

Central imaging evaluation will be performed for the following purpose.

(1) Correlation of the clinical response and pathological evaluation will be examined based on changes in the images of the tumor due to neoadjuvant endocrine therapy.

(2) Central review for clinical evaluation will be performed again, and the concordance rate with the local review for clinical evaluation in each site will be examined.

(3) Standardization of the imaging procedure and improvement of quality control will be sought by investigating the imaging procedure at each center.

14.2 Procedure and items for central imaging evaluation

In this study, ultrasound images before neoadjuvant protocol treatment, during treatment, and after treatment, as well as MRI images before and after treatment will be collected in an electronic file, and central imaging evaluation will be performed. Internal rules/procedures defined by each submitting center should be followed when these images from each center are submitted.

14.3 Submission procedure of image data

Ultrasound and MRI images of enrolled patients will be converted to an electronic format such as JPEG, and be saved on a USB flash memory sent from the CSPOR Data Center before they are returned to the Center using a self-addressed envelope.

14.4 Imaging data to be collected

1) Ultrasound: Before neoadjuvant protocol treatment (at primary enrollment), at 1, 2, and 4 months after the start of neoadjuvant treatment, and at the end of the neoadjuvant protocol or at discontinuation.

2) MRI: Before neoadjuvant protocol treatment (at primary enrollment), and at the end of neoadjuvant protocol or at discontinuation.

The minimum personal identification information whereby the imaging data submitted by each center can be linked to a certain enrolled patient will be attached to the imaging data file. The imaging data file will be saved with the minimum personal identification information, namely, the “center name” and the “subject identification number,” for the folder name and the “imaging type (US, MRI),” “subject identification number,” and “year and month (day) of imaging” for the file name. The name, initials, and/or chart number of patients should not be input in these data for submission.

14.5 Submission procedure of imaging data and place of submission

1) The Data Center will ask for the submission of imaging data and send a submission list and a USB flash memory for submission during the central imaging evaluation.
2) Create a folder by patient based on the submission list, name “center name” and “subject identification number” for the folder name and the “imaging type (US, MRI),” “subject identification number,” and “year and month (day) of imaging” for the file name, and save them on a USB flash memory at each center”.

Submission to: CSPOR Data Center
204 Lions Plaza Ochanomizu, 1-2-12 Yushima, Bunkyo-ku, Tokyo
113-0034, Japan
TEL: 03-3254-8029 FAX: 03-5298-8536

14.6 Planned number of patients for imaging evaluation

Approximately 200 patients among the patients initially enrolled in the study will be included.

15. Study Endpoints

15.1 Primary endpoint

The primary endpoint of this study will be DFS.

(1) Definition
DFS is defined as the time from the date of primary enrollment until the date of the first event.

(2) Event
An event is defined as any one of the following:
1) Diagnosis of recurrence in the ipsilateral preserved breast, the ipsilateral chest wall, or the regional lymph node, or distant organ metastasis (see Section 3.5)
2) Diagnosis of asynchronous breast cancer or any secondary cancer (excluding cutaneous basal cell carcinoma/spindle cell carcinoma, and uterine carcinoma *in situ*)
3) All-cause deaths

(3) Censoring
1) Last date of confirming any of the above-mentioned events
2) Last date of confirming survival for those who survive

15.2 Secondary endpoints

15.2.1 Percentage of patients clinically responding to neoadjuvant LET therapy

Defined as the percentage of patients initiating the neoadjuvant protocol treatment who are judged as having a clinical response of CR or PR to the treatment according to the criteria

specified in Section 3.4.1.

15.2.2 Histological tumor response to neoadjuvant LET therapy

Defined as the percentage of patients initiating the neoadjuvant protocol treatment who are judged as having a histological response of Grade 1b, Grade 2 or Grade 3 to the treatment according to the criteria specified in Section 3.4.2.

15.2.3 Percentage of patients undergoing breast-preserving surgery

Defined as the percentage of patients initiating the neoadjuvant protocol treatment who then undergo breast-preserving surgery.

15.2.4 Overall survival (OS)

(1) Definition
OS is defined as the time from the date of primary enrollment until the date of death from any cause.

(2) Event
An event is defined as death from any cause.

(3) Censoring
1) Last date of confirming survival for those who survive.
2) Last date of confirming survival before loss of follow-up for those who are lost to follow up.

15.2.5 DFS/OS in subgroups of patients according to clinical response (CR, PR, SD or PD) to neoadjuvant LET therapy

DFS and OS will be calculated in individual subgroups of patients according to clinical response (CR, PR, SD or PD) to neoadjuvant LET therapy.

15.2.6 Safety

Adverse events occurring during the neoadjuvant and postoperative protocol treatments will be evaluated using the Japanese (JCOG/JSCO) version (Appendix F) of CTCAE Version 3.0.

15.2.7 HRQOL and pharmacoeconomic assessments

See Section 12: HRQOL and pharmacoeconomic assessments.

15.2.8 Distant disease-free survival (DDFS)

(1) Definition
DDFS is defined as the time from the date of primary enrollment until the date of the first DDFS event.

(2) Events
An event is defined as a diagnosis of distant organ metastasis.

(3) Censoring

Last date on which the above-mentioned event did not occur
In surviving patients, the last date of confirmed survival

16. Statistics

16.1 Statistical methods and criteria used for reaching a conclusion regarding the primary endpoint

The hazard ratio (HR) of DFS in the CL group relative to the L group will be calculated using a proportional hazard model. The HR will be compared with the threshold HRs obtained in a pre-study questionnaire survey (the lowest HR strongly discouraging oncologists to add adjuvant chemotherapy as well as the lowest HR strongly encouraging oncologists to add adjuvant chemotherapy) to reach a conclusion about the choice between LET plus chemotherapy or LET alone. To help interpretation of the data, a posterior distribution of the logarithmic HR will be determined from a non-informative prior distribution using normal approximation, and the posterior probability will be calculated that the logarithmic HR falls into each of the intervals constructed by the above-mentioned threshold HRs. To confirm the proportionality of hazard between groups, log-log plots of the Kaplan-Meier curves will be used. If there is a great deviation from proportional hazards, similar analyses will be performed for 3-5-, 7-, and 10-year cumulative survival rates.

16.2 Justification of the planned sample size and enrollment duration

In this study, the patients choose (determine) one of the two treatments. The primary objective of this study is to make a choice between the two postoperative protocol treatments, i.e., to determine at the moment which treatment, LET alone or LET plus chemotherapy, as postoperative adjuvant therapy will achieve better survival outcome in patients responding to neoadjuvant LET. To prepare for this analysis, all centers scheduled to participate in this study were sent a questionnaire and 78 of them responded to the questionnaire. The results of the questionnaire survey are as follows: the mean predicted 5-year DFS with LET alone was 85.20%; mean highest 5-year DFS with LET plus chemotherapy that would strongly discourage oncologists to add adjuvant chemotherapy was 86.6% (condition A), whereas the mean lowest 5-year DFS with LET plus chemotherapy that would strongly encourage oncologists to add adjuvant chemotherapy was 92.1% (condition B). Assuming an exponential distribution for DFS, the expected HRs for LET plus chemotherapy relative to LET alone under conditions A and B were calculated to be 0.90 and 0.52, respectively.

Based on these survey results, the HR thresholds used to choose between the two treatments were set at 0.9 and 0.6. A total of about 200 events (see Section 14) will be needed to provide a statistical power (referred to as selection probability) of 90% with these thresholds. Assuming that the 5-year DFS in the entire population is 88% and that 90% of the subjects will show CR, PR or SD to neoadjuvant LET, about 1,460 patients in total will be needed to observe occurrence of about 200 events during planned follow-up of up to 8 years (a 3-year enrollment period plus a 5-year follow-up period). By taking account of expected withdrawal of about 10% of the subjects, 1,700 patients in total are planned to be enrolled.

When questioned about the feasibility of this study, centers willing to participate answered that about 800 patients in total potentially eligible for this study would be seen at these centers per year. Therefore, the planned enrollment duration (3 years) is sufficient for achieving the planned sample size.

Although this study was initiated in May 2008 based on the above justification, subject enrollment was actually unsuccessful. Thus, as a result of discussions by the Independent Data Monitoring Committee in March 2011, at 2 years and 10 months after the start of enrollment, it

was approved to extend the enrollment duration from 3 years to 5 years and the study period from 8 years to 10 years. Subsequently, although the number of enrolled patients per month increased, it was impossible to reach the planned sample size of 1700 patients by the day of the end of enrollment (May 2013). Further extension of the enrollment duration was impossible in consideration of the importance of this study, and extension of the follow-up period was proposed based on the opinion that the initial purpose of the study could be attained by extension of the follow-up period, as discussed by the principal investigator, the Data Center representatives, and study statisticians at the steering committee meeting held in January 2012.
Although the follow-up period was set as 5 years until protocol version 1.2, hormone receptor-positive breast cancer frequently relapses more than 5 years postoperatively [39]. Therefore, the follow-up period was extended from 5 to 10 years, and the long-term events were to be followed up, and the planned sample size was calculated again as follows. First, although the selection probability was set as 90% when the initial planned sample size was set, the initial purpose was almost reached, even with a selection probability of 80 to 85%. In such a case, approximately 170 events are required in both groups. Moreover, as described above, an overall number of approximately 630 patients are required when the 5 year DFS is assumed to be 88%, the enrollment duration and follow-up period to be 5 and 10 years, respectively (follow-up period will be up to 15 years). When approximately 1/4 of the initially enrolled patients are not assumed to be enrolled secondarily, approximately 850 patients are finally required overall. This number of patients can be enrolled in this study by May 2013 at the end of enrollment.

16.3 Handling of subjects and analysis sets

Individual subjects will be handled according to criteria that will be drafted by the steering committee before enrollment for each stage of the study and will be finalized by the same committee before the scheduled first interim efficacy analysis. The primary analysis set for each survival variable will be composed of eligible subjects initiating the protocol treatment (i.e., full analysis set). The population for primary analysis of tumor response will be composed of eligible subjects initiating the protocol treatment and having evaluable data. Analysis of adverse events will include all subjects initiating the protocol treatment. The populations for secondary analyses of each survival variable will be defined in the statistical analysis plan that will be finalized by the steering committee before interim analysis.

16.4 Statistical methods used for secondary endpoints

According to criteria for subject handling, point estimates and their confidence intervals will be calculated for percentage of patients clinically responding to neoadjuvant LET, histological response to neoadjuvant LET, and percentage of patients undergoing breast-preserving surgery. For OS and DDFS, cumulative survival rates will be estimated by the Kaplan-Meier method. Confidence intervals for the 1-, 3-, 5-, 7-, and 10-year DFS rates, survival rates and their variance will be constructed using Greenwood's formula. Subgroup analyses will be performed according to important prognostic factors. The following variables will be investigated as prognostic factors for treatment response: Age (<65 and ≥65 years), clinical disease stage (T1c and T2), PgR status (negative and positive), HER2 status (0, 1+, and 2+), histopathology grade (1, 2, and 3), baseline Ki67 expression (<20% and ≥20%), response to neoadjuvant therapy (CR+PR and SD), lymph node infiltration (negative or 1/2/3 involved nodes), surgical procedure (mastectomy and partial lumpectomy), chemotherapy regimen (CMF and AC [EC] and TC and AC-Taxane), factors considered at treatment randomization, and other factors considered during blinded review. Cox regression analysis will also be performed for these factors. Moreover, confidence intervals will be calculated for restricted mean disease-free survival time (RMDFST) in each group at 1, 3, 5, 7 and 10 years and their variances. Descriptive statistics will be presented for DFS as the total incidence per event in each group, and each event will also be analyzed as the endpoint. Specifically, the following events will be investigated: distant recurrence, local recurrence within the preserved breast, ipsilateral chest wall recurrence, regional lymph node recurrence, contralateral breast cancer, secondary cancer, death and other events. Kaplan-Meier analysis of grouped DFS and OS by histological response to neoadjuvant therapy (CR+PR, SD, and PD) will be performed to determine the cumulative survival rate for each survival time.

16.5 Analysis of safety

For each group, incidence of adverse events during each course will be calculated by grade. For most common adverse events, cumulative incidence over time will be determined by the Kaplan-Meier method. For each adverse event, the worst grades in individual patients will be determined and will be compared between the two groups using the Mantel test. Analyses of grade 3 or severer adverse events only will also be performed.

16.6 Additional analyses

Exploratory analyses will be performed, if appropriate. Further details of all analyses will be

specified in the statistical analysis plan. Criteria for subject handling and statistical analysis plan will be prepared separately for HRQOL and pharmacoeconomic assessments.

16.7 Blinded monitoring

The steering committee will monitor the subject accrual and survival across groups in a blinded manner. By reference to newly published results of other clinical studies, the committee will reconsider the necessary number of subjects (events) and the appropriateness of the planned timing of interim and final analyses as well as publication of the results. When it becomes necessary to change the protocol, the committee will propose the change to the Institutional Review Board and the Independent Data Monitoring Committee. After the validity of the proposed change has been reviewed and approved by these committees, analyses will be performed under the changed protocol. Before the scheduled first interim efficacy analysis, the steering committee will perform blinded review of data to help finalize the details of the statistical analysis plan.

16.8 Interim analyses

The Independent Data Monitoring Committee will perform interim analysis and evaluate the antitumor effects of letrozole (LET) when the response to neoadjuvant therapy is determined in 140 patients to confirm the efficacy of neoadjuvant LET treatment.
When the number of enrolled patients is considered for the denominator and the hypothesis that the proportion of patients with ≥ SD is below 75% is not rejected using a significance level of 5%, it is judged that neoadjuvant LET treatment is unlikely to obtain the expected effect, and the steering committee was recommended to terminate the study or change the protocol. When the hypothesis is rejected, it is judged that neoadjuvant LET treatment is likely to obtain the expected effect, and the enrollment is continued.

When the proportion of the threshold clinical response ≥ SD is 75%, and the proportion of the expected clinical response is 85% (corresponding to 80% and 90%, respectively, when evaluable patients are considered as the denominator) with error α 0.05 and error ß 0.1, approximately 140 patients are required based on binomial distribution.

17. Ethics

17.1 Patient protection

All study-related personnel will conduct this study in compliance with the Declaration of Helsinki (Appendix E) and the Ethical Guidelines for Medical and Health Research Involving Human Subjects established by the Ministry of Health, Labour and Welfare on December 22, 2014 (partially revised on February 28, 2017).

17.2 Informed consent

17.2.1 Subject information

Prior to primary enrollment, the subinvestigator will provide each candidate subject with written information approved by the center's ethical committee or the Institutional Review Board (IRB) (a sample document shown in Appendix A, appropriately revised at each center) and a detailed oral explanation of the following:

1) Patient's illness

2) That this is a clinical study

3) The design of and rationale for this study (e.g., significance of the study, expected number of patients enrolled in the study, need of the study, and objectives of the study)

4) Details of the protocol treatments (e.g., drug name, dosage, dose per time, and duration of treatment)

5) Expected effects of the protocol treatments

6) Foreseeable adverse events and their treatments

7) Expenses of participation and available compensations (that all these are the same as in general practice; that the expenses of the protocol treatments will be covered by the patient's health insurance and that the patient will be compensated for any health injury according to the rule applicable to general practice)

8) Alternative treatments
Details of alternative treatments currently available in practice

9) Expected benefits and foreseeable risks of participation in the study

10) Direct access to the patient's medical history
Explanation of acceptance of audits

11) Refusal to participate and withdrawal of consent
That the patient is free to refuse to participate and to withdraw her consent once given without penalty or loss of benefits otherwise entitled in the treatment of her illness.

12) Protection of subject's rights
That greatest effort will be made to keep the confidentiality of subject identities (e.g., subject name).

13) Secondary use of data from the study
    That data from the study may be used for secondary purposes without linkage to information that can identify individual subjects.

14) Subject's right to make questions
    That the subject will be informed in writing of how to contact her physician (subinvestigator) and the principal investigator (or the study administrative office) and can make any question to these individuals about this study and treatment of her illness.

17.2.2 Procedure for obtaining informed consent

After informing a candidate subject about this study and confirming that she has well understood the explanation, the physician will ask her to participate in this study. If the patient agrees to participate, the informed consent form designated at the center will be signed and personally dated by the physician involved in the informed consent discussion and the patient. Two duplicate copies of the signed informed consent form will be prepared. One of them will be handed over to the patient, while the other will be kept at the center. The original consent form will be attached to the patient's medical record.

17.2.3 Timing of obtaining informed consent

Informed consent must be obtained from a patient before her enrollment.

17.3 Confidentiality and subject identification

The name of any patient enrolled will not be reported from the center to the CSPOR Data Center. Individual patients enrolled will be identified or referenced by their subject identification numbers, initials, and/or dates of birth. Information that can directly identify individual patients (e.g., names) will not be entered into the study database (database managed by the Data Center). To keep the confidentiality of each subject's identity, her initials and the day of her birth may be masked by entering "x" to the corresponding space.
Subject data will be transmitted between centers, the CSPOR Data Center, and the CSPOR Administrative Office by the following means:

1) Facsimile transmission for subject enrollment, references of urgent need, and expedited adverse event reporting
2) Electronic data transmission with encrypting function for entering data via the EDC system
3) Postal mail or personal transmission of hard copies of questionnaires/report forms

17.4 Compliance with the protocol

All research individuals involved in this study will comply with this protocol unless this may potentially jeopardize the subject's safety or rights.

17.5 Approval by the center's ethical committee or the Institutional Review Board (IRB)

17.5.1 Approval of center's participation

Prior to each center's participation in this study, this protocol and written subject information must be approved by the center's ethical committee or IRB. If IRB approval is obtained, a copy of written IRB approval will be sent to the CSPOR Administrative Office. The original written IRB approval will be kept at the center, while its copy will be kept at the CSPOR Administrative Office.

17.5.2 Continued (annual) IRB review and approval

Continued (annual) review and approval of this protocol and written subject information by each center's ethical committee or IRB will follow each center's rules.

18. Monitoring and Auditing

18.1 Monitoring

18.1.1 Objective of monitoring

The objective of monitoring is to ensure that the study is conducted safely and in compliance with the protocol and that accurate data are collected.

18.1.2 In-house monitoring

The Executive Committee and the CSPOR Data Center will cooperate in monitoring data collected via the EDC system and HRQOL questionnaires by reference to the results of processing electronic data. On-site monitoring is not planned.

18.1.3 Monitoring of individual subjects

1) Subject's eligibility
2) Protocol treatment status (especially whether the protocol treatment has been terminated)
3) Whether any adverse event, especially any serious adverse event has occurred and has been reported appropriately.
4) Follow-up and survival status after conclusion of protocol treatments
5) Other items

18.1.4 Monitoring of study progress

1) Accrual in individual groups
2) Overall subjects' eligibility
3) Protocol treatment status
4) Adverse event status
5) Follow-up status
6) Other items

18.2 Auditing

18.2.1 Objective of auditing

The objective of auditing is to determine whether this study has been conducted properly and to assure the reliability of data arising from the study. Audits will be conducted as the need arises.

18.2.2 Auditing committee

To audit this study, the Auditing Committee will be established at EP-CRSU Co., Ltd. The Auditing Committee will be composed of physicians or experts of equivalent qualifications who have full knowledge and experience in clinical research and are not directly involved in the care of the patients to be audited. The Auditing Committee Chair will be selected from the committee members.

18.2.3 Duties of the auditing committee

1) To audit the study in order to assure that individual patients enrolled are eligible for the study and that medical treatments, clinical and laboratory monitoring, and follow-ups have been performed in compliance with the protocol.

2) To prepare the standard operating procedures (SOPs) and manuals for auditing the study and to perform on-site auditing of the CSPOR Data Center and individual study centers according to the auditing plan.

3) On-site auditing of study centers
   To serially visit the study centers and have direct access to all study-related documents and source documents on the subjects (e.g., office records and imaging data) in order to confirm that this study has been conducted in compliance with the ICH-GCP guideline and this protocol.

4) Auditing of the CSPOR Data Center
   To audit the works of the CSPOR Data Center and to direct the center to perform its duties in accordance with its relevant SOPs.

5) Preparation of an auditing report
   For each auditing activity made according to its duties, to prepare a written report of the findings (auditing report) and an auditing certificate which certifies that auditing has been performed, and to submit these documents to the principal investigator.

18.2.4 Confidentiality

In auditing the study, the Auditing Committee will make efforts to keep the confidentiality of records and medical information that identify individual subjects.

18.2.5 Extramural review by the auditing committee

The Auditing Committee will perform extramural review of patients who have experienced recurrence, died, or developed any serious or unexpected adverse event and will report the results of review to the principal investigator. Based on the results, the principal investigator and the Executive Committee will discuss the need of monitoring any study center and will perform necessary monitoring.

19. Publication of Study Findings

Study findings will be published according to the policies established by the Institutional Review Board. Publication about this study will be made at the stages of planning and obtaining interim and final results. Presentations will be made at scientific meetings and/or manuscripts will be submitted for publication in medical journals. At the stage of planning, the outline of the study based on this protocol will be published at appropriate timing. Interim results of the study will be published as soon as possible after preplanned interim analyses have given any notable result. Unless the study is prematurely terminated, final results of the study (results of confirmative analysis of the primary endpoint) will be published after closure of follow-up of all subjects.

Whether study findings are published at a scientific meeting or in a medical journal, the authors and their order will follow the above-mentioned policies and the publication will receive prior review and approval of the Institutional Review Board and the Independent Data Monitoring Committee.

20. Conflict of Interest and Source of Funding

No individuals involved in this study will have any potential conflict of interest in planning, executing or reporting this study. In this context, conflicts of interest indicate both financial and private interests that may affect findings from the study.

This study will be funded by the CSPOR. The CSPOR Administrative Office will perform all secretarial works for the study but will not make any decision on the planning, executing or reporting of the study. The Executive Committee, which will be organized for this study, will make all decisions on the planning, executing, and reporting of the study.

21. Clinical Study Registration

This study will be registered at the UMIN Clinical Trials Registry (UMIN-CTR; http://www.umin.ac.jp/ctr/index-j.htm) to make the study outline public. Registration will be made prior to enrollment of the first subject by the CSPOR Administrative Office.

The UMIN-CTR is a clinical trial registry which meets the international requirements for such registries. Need of registering clinical studies/trials has been an issue since the 1970s. In September 2004, the International Committee of Medical Journal Editors (ICMJE) announced its policy which required prior registration of a clinical study for the acceptance of an article about the study for publication in a medical journal,[40,41] which has led to the start of several clinical trial registry systems. The World Health Organization (WHO) has taken the initiative by launching the International Clinical Trials Registry Platform and establishing the requirements for registration[42] in April 2005. In the light of this international trend, the UMIN-CTR was launched in June 2005 as the first Japanese clinical trial registry. The UMIN-CTR has been recognized as an acceptable registry by the ICMJE and has also acted in concert with the WHO's clinical trial registry system.

As of March 2019, the latest study-related information has been published on the Japan Registry of Clinical Trials (jRCT)–a web-based registry for public disclosure of clinical study protocols and summaries of study data. This registry was established to facilitate the submission of study protocols and other notifications to the Minister of Health, Labour and Welfare in accordance with the provisions of the "Clinical Trials Act" and the "Act on Securing Quality, Efficacy and Safety of Pharmaceuticals, Medical Devices, Regenerative and Cellular Therapy Products, Gene Therapy Products, and Cosmetics" (PMDA Act) concerning clinical research

performed by medical institutions and other healthcare providers in order to ensure the disclosure of information required to be published by the World Health Organization (WHO) and to guarantee the transparency of study processes pursuant to the standards on clinical trial conduct stipulated in the above-mentioned legislation, as well as publishing relevant study information to assist potential volunteers in making informed choices regarding participation in national clinical studies.

22. Related Studies

In relation to this study, the following studies will be performed, for which protocols will be prepared separately:

1) An epidemiological study: Multipurpose Cohort Study 06 in Breast Cancer Patients
2) Translational research

23. Study Organization and Administration

This is one of the clinical studies conducted under the sponsorship of the Comprehensive Support Project for Oncology Research (CSPOR). The organizations involved in this study are listed below. At the top of the list is the Executive Committee, an organization specific to this study. This is followed by various committees which commonly work for the entire project.

23.1 N-SAS BC 06 executive committee

Chair (Principal Investigator)
Hiroji Iwata (Department of Breast Oncology, Aichi Cancer Center: Principal Investigator)
Study Statistician (Director of Biostatistics)
Takuhiro Yamaguchi (Division of Biostatistics, Tohoku University Graduate School of Medicine)
Steering Committee Members (in Kana order)
Shoichiro Ohtani (Department of Breast Surgery, Hiroshima City Hospital)
Masahiro Kashiwaba (Adachi Nyusen Clinic [Adachi Breast Clinic], Kyoto Breast Center, Medical Foundation Imai-kai Adachi Hospital)
Naruto Taira (Department of Breast and Endocrine Surgery, Okayama University Graduate School of Medicine, Dentistry, and Pharmaceutical Sciences)
Tatsuya Toyama (Department of Breast and Endocrine Surgery, Nagoya City University Hospital)
Tomomi Fujisawa (Department of Breast Oncology, Gunma Prefectural Cancer Center)
Norikazu Masuda (Department of Surgery, National Hospital Organization, Osaka National Hospital)
Yutaka Yamamoto (Department of Breast and Endocrine Surgery, Kumamoto University Hospital)

23.2 Institutional Review Board

Up to March 31, 2017:
Chair
Tatsuya Toyama (Department of Breast and Endocrine Surgery, Nagoya City University Hospital)
Vice chair
Eishin Boku (Department of Breast Surgery, Tohoku Medical and Pharmaceutical University Hospital)
Committee Members
Satoshi Teramukai (Study Statistician; Department of Biostatistics, Graduate School of Medicine, Kyoto Prefectural University of Medicine)
Toshimi Takano (Department of Medical Oncology, Toranomon Hospital)
Kenji Tamura (Department of Breast and Medical Oncology, National Cancer Center Hospital Central Hospital)
Nobuaki Matsubara (Department of Breast and Medical Oncology, National Cancer Center Hospital East)

23.3 Independent Data Monitoring Committee

Chair
Seigo Nakamura (Department of Breast Surgery, Showa University Hospital)
Vice chair
Noriyuki Katsumata (Department of Medical Oncology, Nippon Medical School Musashikosugi Hospital)
Committee Members
Taro Shibata (Research Support Center, National Cancer Center)
Akihiro Yanagisawa (Medical Mobile Communications Co., Ltd.)

23.4 Data Center (formerly CSPOR Data Center)

Responsible for enrollment, study progress management, monitoring, and data management operations.

Up to March 31, 2020:
EP CRSU Co., Ltd. (formerly NPO Japan Clinical Research Support Unit [J-CRSU])
3F Acropolis Tokyo,
6-29 Shinogawa-machi, Shinjuku-ku, Tokyo 162-0814, Japan
Tel: 03-6759-9908
Fax: 03-5946-8274
Email: support@csp.or.jp

Up to April 1, 2020:
Data Management Office, General Affairs Department,
Comprehensive Support Project (CSP),
Public Health Research Foundation
1-1-7 Nishiwaseda, Shinjuku-ku, Tokyo 169-0051, Japan
Tel: 03-5287-2635
Fax: 03-5287-2644
Email: phrf-dm@csp.or.jp

23.5 Office of Comprehensive Support Project for Oncological Research (CSPOR office)

Address: 3F, 1-1-7 Nishiwaseda, Shinjuku-ku Tokyo
169-0051, Japan
Public Health Research Foundation
Tel: 03-5287-2633
Fax: 03-5287-2634
E-mail: info@csp.or.jp

24. Protocol Changes and Study Termination

24.1 Protocol changes

24.1.1 Classification of protocol changes

Changes to the protocol that has once approved by the Clinical Study Review Committee will be classified into amendments and revisions, which will be handled separately. Protocol amendments and revisions, defined below, will be made as follows:

(1) Amendments
A protocol amendment is defined as a partial change to the protocol which may increase the risk to the study subjects or is relevant to the primary endpoint of the study.
A protocol amendment must be reviewed and approved by the Independent Data Monitoring Committee and each center's ethical committee.
The cover page of the amended protocol will bear the date of approval by the Independent Data Monitoring Committee.

(2) Revisions
A protocol revision is defined as a change to the protocol which is unlikely to increase the risk to the study subjects and is not relevant to the primary endpoint of the study.
A protocol revision need not be reviewed by the Independent Data Monitoring Committee but must be approved by the Executive Committee Chair and reported to the Independent Data Monitoring Committee. Whether a protocol revision needs review and approval of each center's ethical committee depends on the rules of the center.
The cover page of the revised protocol will bear the date of approval by the Executive Committee Chair.

24.1.2 Approval of protocol amendments/revisions by each center's ethical committee

Any amendment to this protocol or written subject information made during the study and approved by the Independent Data Monitoring Committee must receive approval of each center's ethical committee (or IRB) before implementation.
Whether a revision to the protocol or written subject information needs review and approval of each center's ethical committee (or IRB) depends on the rules of the center.
After an amendment has received IRB approval, the clinical study coordinator at the center will send a copy of written IRB approval to the CSPOR Administrative Office. The original written IRB approval will be kept at the center, while its copy will be kept at the Administrative Office.

24.2 Study termination

The Independent Data Monitoring Committee will review the appropriateness of continuing the study based on safety data and the results of interim analyses of efficacy. If the Committee judges study continuation inappropriate, the Committee will recommend the Executive Committee to terminate or suspend the study. If the Executive Committee decides to terminate the study according to the recommendation, the principal investigator will inform all investigators as soon as possible of study termination, the reasons for the action, and measures to be taken for patients remaining on the study. The investigator at each center will report study termination and the reasons for it to the center's IRB and will take appropriate measures for patients remaining on the

study according to the directions of the principal investigator and the center's IRB.

25. List of Study Centers

A list of the study sites currently participating in this clinical study is available on the Japan Registry of Clinical Trials (jRCT) website (https://jrct.niph.go.jp/).
Clinical Study Protocol No. jRCTs041180095

26. References

1. The Editorial Board of the Cancer Statistics in Japan. Cancer Statistics in Japan 2007. Tokyo: Foundation for Promotion of Cancer Research (FPCR). 2007. Available from: www.fpcr.or.jp.
2. Goldhirsch A, Wood W, Gelber R, Coates A, Thurlimann B, Senn HJ, et al. Progress and promise: highlights of the international expert consensus on the primary therapy of early breast cancer 2007. Ann Oncol. 2007; 18: 1133-44.
3. Goldhirsch A, Glick JH, Gelber RD, Coates AS, Thurlimann B, Senn HJ; Panel members. Meeting highlights: international expert consensus on the primary therapy of early breast cancer 2005. Ann Oncol. 2005; 16: 1569-83.
4. Goldhirsch A, Coates AS, Gelber RD, Glick JH, Thurlimann B, Senn HJ; St Gallen Expert Panel Members. First--select the target: better choice of adjuvant treatments for breast cancer patients. Ann Oncol. 2006; 7: 1772-6.
5. Early Breast Cancer Trialists' Collaborative Group (EBCTCG). Effects of chemotherapy and hormonal therapy for early breast cancer on recurrence and 15-year survival: an overview of the randomised trials. Lancet. 2005; 365: 1687-717.
6. Howell A, Cuzick J, Baum M, Buzdar A, Dowsett M, Forbes JF, et al.; ATAC Trialists' Group. Results of the ATAC (Arimidex, Tamoxifen, Alone or in Combination) trial after completion of 5 years' adjuvant treatment for breast cancer. Lancet. 2005; 365: 60-2.
7. Coombes RC, Kilburn LS, Snowdon CF, Paridaens R, Coleman RE, Jones SE, et al.; Intergroup Exemestane Study. Survival and safety of exemestane versus tamoxifen after 2-3 years' tamoxifen treatment (Intergroup Exemestane Study): a randomised controlled trial. Lancet. 2007; 369: 559-70.
8. Coates AS, Keshaviah A, Thurlimann B, Mouridsen H, Mauriac L, Forbes JF, et al. Five years of letrozole compared with tamoxifen as initial adjuvant therapy for postmenopausal women with endocrine-responsive early breast cancer: update of study BIG 1-98. J Clin Oncol. 2007; 25: 486-92.
9. Goss PE, Ingle JN, Martino S, Robert NJ, Muss HB, Piccart MJ, et al. Randomized trial of letrozole following tamoxifen as extended adjuvant therapy in receptor-positive breast cancer: updated findings from NCIC CTG MA.17. J Natl Cancer Inst. 2005; 97: 1262-71.
10. Winer EP, Hudis C, Burstein HJ, Wolff AC, Pritchard KI, Ingle JN, et al. American Society of Clinical Oncology technology assessment on the use of aromatase inhibitors as adjuvant therapy for postmenopausal women with hormone receptor-positive breast cancer: status report 2004. J Clin Oncol. 2005; 23: 619-29.
11. NCCN Clinical Practice Guidelines in Oncology™ Breast Cancer v.2. 2007. Available from: www.nccn.org.
12. Bhatnagar AS, Hausler A, Schieweck K, Lang M, Bowman R. Highly selective inhibition of estrogen biosynthesis by CGS 20267, a new non-steroidal aromatase inhibitor. J Steroid Biochem Mol Biol. 1990; 37: 1021-7.
13. Geisler J, Haynes B, Anker G, Dowsett M, Lonning PE. Influence of letrozole and anastrozole on total body aromatization and plasma estrogen levels in postmenopausal breast cancer patients evaluated in a randomized, cross-over study. J Clin Oncol. 2002; 20: 751-7.
14. Novartis Pharma K.K. Drug Interview Form: FEMARA® Tablet 2.5 mg, May 2007 (new format version 3)
15. Mouridsen H, Gershanovich M, Sun Y, Perez-Carrion R, Boni C, Monnier A, et al. Phase III study of letrozole versus tamoxifen as first-line therapy of advanced breast cancer in postmenopausal women: analysis of survival and update of efficacy from the International Letrozole Breast Cancer Group. J Clin Oncol. 2003; 21: 2101-9.

16. Eiermann W, Paepke S, Appfelstaedt J, Llombart-Cussac A, Eremin J, Vinholes J, et al.; Letrozole Neo-Adjuvant Breast Cancer Study Group. Preoperative treatment of postmenopausal breast cancer patients with letrozole: A randomized double-blind multicenter study. Ann Oncol. 2001; 12: 1527-32.
17. Ellis MJ, Coop A, Singh B, Mauriac L, Llombert-Cussac A, Janicke F, et al. Letrozole is more effective neoadjuvant endocrine therapy than tamoxifen for ErbB-1- and/or ErbB-2-positive, estrogen receptor-positive primary breast cancer: evidence from a phase III randomized trial. J Clin Oncol. 2001; 19: 3808-16.
18. Paepke S, Tulusan A, Kiesel L, Bastert G, Jaenicke FK, Bouterfa H, et al.; A multi-center study of pre-operative treatment with Letrozole for optimal duration of treatment in postmenopausal women with ER and/or PGR positive breast cancer. Proc Am Soc Clin Oncol 22: 2003 (abstr 321)
19. Kaufmann M, Hortobagyi GN, Goldhirsch A, Scholl S, Makris A, Valagussa P, et al. Recommendations from an international expert panel on the use of neoadjuvant (primary) systemic treatment of operable breast cancer: an update. J Clin Oncol. 2006; 24: 1940-9.
20. Ring AE, Smith IE, Ashley S, Fulford LG, Lakhani SR. Oestrogen receptor status, pathological complete response and prognosis in patients receiving neoadjuvant chemotherapy for early breast cancer. Br J Cancer. 2004; 91: 2012-7.
21. International Breast Cancer Study Group. Endocrine responsiveness and tailoring adjuvant therapy for postmenopausal lymph node-negative breast cancer: a randomized trial. J Natl Cancer Inst. 2002; 94: 1054-65.
22. Paik S. Development and clinical utility of a 21-gene recurrence score prognostic assay in patients with early breast cancer treated with tamoxifen. Oncologist. 2007; 12: 631-5.
23. Bogaerts J, Cardoso F, Buyse M, Braga S, Loi S, Harrison JA, et al.; TRANSBIG consortium. Gene signature evaluation as a prognostic tool: challenges in the design of the MINDACT trial. Nat Clin Pract Oncol. 2006; 3: 540-51.
24. Land SR, Wickerham DL, Costantino JP, Ritter MW, Vogel VG, Lee M, et al. Patient-reported symptoms and quality of life during treatment with tamoxifen or raloxifene for breast cancer prevention: the NSABP Study of Tamoxifen and Raloxifene (STAR) P-2 trial. JAMA. 2006; 295: 2742-51.
25. Bernhard J, Zahrieh D, Castiglione-Gertsch M, Hurny C, Gelber RD, Forbes JF, et al.; International Breast Cancer Study Group Trial VIII. Adjuvant chemotherapy followed by goserelin compared with either modality alone: the impact on amenorrhea, hot flashes, and quality of life in premenopausal patients--the International Breast Cancer Study Group Trial VIII. J Clin Oncol. 2007; 25: 263-70.
26. Fallowfield LJ, Bliss JM, Porter LS, Price MH, Snowdon CF, Jones SE, et al. Quality of life in the intergroup exemestane study: a randomized trial of exemestane versus continued tamoxifen after 2 to 3 years of tamoxifen in postmenopausal women with primary breast cancer. J Clin Oncol. 2006; 24: 910-7.
27. Whelan TJ, Goss PE, Ingle JN, Pater JL, Tu D, Pritchard K, et al. Assessment of quality of life in MA.17: a randomized, placebo-controlled trial of letrozole after 5 years of tamoxifen in postmenopausal women. J Clin Oncol. 2005; 23: 6931-40.
28. Oken MM, Creech RH, Tormey DC, Horton J, Davis TE, McFadden ET, et al. Toxicity and response criteria of the Eastern Cooperative Oncology Group. Am J Clin Oncol. 1982; 5: 649-55.
29. Therasse P, Arbuck SG, Eisenhauer EA, Wanders J, Kaplan RS, Rubinstein L, et al. New guidelines to evaluate the response to treatment in solid tumors. European Organization for Research and Treatment of Cancer, National Cancer Institute of the United States, National Cancer Institute of Canada. J Natl Cancer Inst. 2000; 92: 205-16.

30. Fallowfield LJ, Leaity SK, Howell A, Benson S, Cella D: Assessment of quality of life in women undergoing hormonal therapy for breast cancer: validation of an endocrine symptom subscale for the FACT-B. Breast Cancer Res Treat. 1999; 55: 189-99.
31. Cella DF, Tulsky DS, Gray G, Sarafian B, Linn E, Bonomi A, et al.: The Functional Assessment of Cancer Therapy scale: development and validation of the general measure. J Clin Oncol. 1993; 11: 570-9.
32. Brady MJ, Cella DF, Mo F, Bonomi AE, Tulsky DS, Lloyd SR, et al.: Reliability and validity of the Functional Assessment of Cancer Therapy-Breast quality-of-life instrument. J Clin Oncol. 1997; 15: 974-86.
33. Zigmond AS, Snaith RP. The hospital anxiety and depression scale. Acta Psychiatr Scand 1983; 67: 361-70.
34. Zigmond AS, Anith RP, Kitamura T: Hospital Anxiety and Depression Scale. Arch Psychiatr Diag Clin Eval. 1993; 4: 371-2.
35. Brooks R with the EuroQol Group. EuroQol: the current state of play. Health Policy 1996; 37: 58-72.
36. Japanese EuroQol Translation Team: Development of the Japanese Version of EuroQol. J Health Care Med Commun. 1998; 8: 109-23.
37. Tsuchiya A, Ikeda S, Ikegami N, Nishimura S, Sakai I, Fukuda T, et al. Estimating an EQ-5D population value set: the case of Japan. Health Econ. 2002; 11: 341-53.
38. Gold MR, Siegel JE, Russell LB, Weinstein MC. Cost-effectiveness in health and medicine. New York: Oxford University Press; 1996.
39. Saphner T, Tormey DC, Gray R. Annual hazard rates of recurrence for breast cancer after primary therapy. J Clin Oncol. 1996;14:2738-46.
40. Drummond MF, O'Brien B, Stoddart GL, Torrance GW. Methods for the economic evaluation of health care programmes. 2nd ed. New York: Oxford University Press; 1997.
41. International Committee of Medical Journal Editors. Uniform Requirements for Manuscripts Submitted to Biomedical Journals: Writing and Editing for Biomedical Publication. Updated February 2006. Available from: http://www.icmje.org/index.html.
42. De Angelis C, Drazen JM, Frizelle FA, Haug C, Hoey J, Horton R, et al.; International Committee of Medical Journal Editors. Clinical trial registration: a statement from the International Committee of Medical Journal Editors. N Engl J Med. 2004; 351: 1250-1.
43. World Health Organization. Technical Consultation on Trial Registration Standards; 2005 Apr 24–27; Geneva, Switzerland. Available from: http://www.who.int/ictrp/news/past_events/en/index.html.

No.	Page No.	Original (version 2.1)	Revision (version 2.2)	Rationale & Description of Protocol Changes
1	Header	N-SAS BC 06 version 2.1 June 1, 2012	N-SAS BC 06 version 2.2 February 1, 2021	Preparation of revised version
2	Cover page	Comprehensive Support Project for Oncology Research (CSPOR)	Comprehensive Support Project for Oncology Research (CSPOR)	Change to corporate entity.
3	Cover page	Principal Investigator<br>Department of Breast Oncology, Aichi Cancer Center Hospital	Principal Investigator<br>Department of Breast Oncology, Aichi Cancer Center	Change to name of affiliated organization
4	Cover page	CSPOR Data Center<br>Yasuo OHASHI<br>Department of Health Sciences and Nursing, Graduate School of Medicine,<br><br>the University of Tokyo<br>204 Lions Plaza Ochanomizu<br>1-2-12 Yushima, Bunkyo-ku, Tokyo<br>113-0034, Japan<br>Phone: 03-3254-8029<br>Fax: 03-5298-8536<br>E-mail: support@csp.or.jp	Data Center<br>Data Management Office, General Affairs Department, Comprehensive Support Project (CSP), Public Health Research Foundation<br>1-1-7 Nishiwaseda, Shinjuku-ku, Tokyo 169-0051, Japan<br>Phone: 03-5287-2635<br>Fax: 03-5287-2644<br>E-mail: phrf-dm@csp.or.jp	Change of contracted data center.
5	Cover page	(No text)	Version 2.2 prepared: On February 1, 2021	Specified the date on which the revised version of the protocol was prepared.
6	P 4	0.2 Objectives<br>Secondary endpoints：OS, percentage of patients clinically responding to neoadjuvant LET, histological tumor response to neoadjuvant LET, percentage of patients undergoing breast-preserving surgery, DFS/OS in patients showing CR, PR, SD or PD response to neoadjuvant LET, safety, HRQOL, and cost-effectiveness (cost utility).	0.2 Objectives<br>Secondary endpoints：OS, distant disease-free survival (DDFS), percentage of patients clinically responding to neoadjuvant LET, histological tumor response to neoadjuvant LET, percentage of patients undergoing breast-preserving surgery, DFS/OS in patients showing CR, PR, SD or PD response to neoadjuvant LET, safety, HRQOL, and cost-effectiveness (cost utility).	Changed due to addition of analytical methods.
7	P7	1.2 Secondary endpoints<br>Secondary endpoints of this study will include OS, percentage of patients clinically responding to neoadjuvant LET, histological tumor response to neoadjuvant LET, percentage of patients undergoing breast-preserving surgery, DFS/OS in subgroups of patients according to clinical response (CR, PR, SD or PD) to neoadjuvant LET, safety, HRQOL, and cost-effectiveness (cost-utility).	1.2 Secondary endpoints<br>Secondary endpoints of this study will include OS, distant disease-free survival (DDFS), percentage of patients clinically responding to neoadjuvant LET, histological tumor response to neoadjuvant LET, percentage of patients undergoing breast-preserving surgery, DFS/OS in subgroups of patients according to CR, PR, SD or PD to neoadjuvant LET, safety, HRQOL, and cost-effectiveness (cost-utility).	Changed due to addition of analytical methods.
8	P16	2.9 Comprehensive Support Project for Oncology Research (CSPOR)<br>In 2000, the Public Health Research Foundation (PHRF) launched a project for supporting development of breast cancer treatments, including sociopsychological interventions, to increase QALY in breast cancer patients. The PHRF was founded in 1984 and certified as a designated public interest corporation by the Ministry of Health, Labour and Welfare. The missions of this corporation are to promote studies of the effects of stress on human mental and physical health, to facilitate use of the findings from such studies for disease prevention and health promotion, and thus to contribute to the maintenance and improvement of public health.	2.9 Comprehensive Support Project for Oncology Research (CSPOR)<br>In 2000, the public interest incorporated foundation Public Health Research Foundation (PHRF; formerly an incorporated foundation) launched a project for supporting development of breast cancer treatments, including sociopsychological interventions, to increase QALY in breast cancer patients. The PHRF was founded in 1984 and certified as a designated public interest corporation by the Ministry of Health, Labour and Welfare. The missions of this corporation are to promote studies of the effects of stress on human mental and physical health, to facilitate use of the findings from such studies for disease prevention and health promotion, and thus to contribute to the maintenance and improvement of public health.	Change to corporate entity.
9	P61	(No text)	15.2.8 Distant disease-free survival (DDFS)<br>(1) Definition<br>DDFS is defined as the time from the date of primary enrollment until the date of the first DDFS event.<br>(2) Events<br>An event is defined as a diagnosis of distant organ metastasis.<br>(3) Censoring<br>Last date on which the above-mentioned event did not occur<br>In surviving patients, the last date of confirmed survival	Changed due to addition of analytical methods.

No.	Page No.	Original (version 2.1)	Revision (version 2.2)	Rationale & Description of Protocol Changes
10	P63	16.4 Statistical methods used for secondary endpoints<br>According to criteria for subject handling, point estimates and their confidence intervals will be calculated for percentage of patients clinically responding to neoadjuvant LET, histological response to neoadjuvant LET, and percentage of patients undergoing breast-preserving surgery. For DFS and OS, cumulative survival rates will be estimated by the Kaplan-Meier method. Confidence intervals around the 1-, 3-, 5-, 7-, and 10- year survival rates will be constructed using Greenwood's formula. Subgroup analyses will be performed according to important prognostic factors. These analyses will take account of the stratification factors used for randomization and other factors considered during blinded review. In addition, Cox regression analysis will be performed, taking account of these factors.	16.4 Statistical methods used for secondary endpoints<br>According to criteria for subject handling, point estimates and their confidence intervals will be calculated for percentage of patients clinically responding to neoadjuvant LET, histological response to neoadjuvant LET, and percentage of patients undergoing breast-preserving surgery. For OS and DDFS, cumulative survival rates will be estimated by the Kaplan-Meier method. Confidence intervals for the 1-, 3-, 5-, 7-, and 10-year DFS rates, survival rates and their variance will be constructed using Greenwood' s formula. Subgroup analyses will be performed according to important prognostic factors. The following variables will be investigated as prognostic factors for treatment response: Age (<65 and ≥65 years), clinical disease stage (T1c and T2), PgR status (negative and positive), HER2 status (0, 1+, and 2+), histopathology grade (1, 2, and 3), baseline Ki67 expression (<20% and ≥20%), response to neoadjuvant therapy (CR+PR and SD), lymph node infiltration (negative or 1/2/3 involved nodes), surgical procedure (mastectomy and partial lumpectomy), chemotherapy regimen (CMF and AC [EC] and TC and AC-Taxane), factors considered at treatment randomization, and other factors considered during blinded review. Cox regression analysis will also be performed for these factors. Moreover, confidence intervals will be calculated for restricted mean disease-free survival time (RMDFST) in each group at 1, 3, 5, 7 and 10 years and their variances. Descriptive statistics will be presented for DFS as the total incidence per event in each group, and each event will also be analyzed as the endpoint. Specifically, the following events will be investigated: distant recurrence, local recurrence within the preserved breast, ipsilateral chest wall recurrence, regional lymph node recurrence, contralateral breast cancer, secondary cancer, death and other events. Kaplan-Meier analysis of grouped DFS and OS by histological response to neoadjuvant therapy (CR+PR, SD, and PD) will be performed to determine the cumulative survival rate for each survival time.	Added analytical methods.
11	P65	17 Ethics<br>17.1 Patient protection<br>All research individuals involved in this study will comply with the Declaration of Helsinki (Appendix E) and the Ethical Guides to Clinical Investigations established by the Ministry of Health, Labour and Welfare (http://www.imcj.go.jp/rinri/index.html) in conducting this study.	17 Ethics<br>17.1 Patient protection<br>All study-related personnel will conduct this study in compliance with the Declaration of Helsinki (Appendix E) and the Ethical Guidelines for Medical and Health Research Involving Human Subjects established by the Ministry of Health, Labour and Welfare on December 22, 2014 (partially revised on February 28, 2017).	Changed to most recent policy.
12	P67	18.2.1 Objective of auditing<br>The objective of auditing is to determine whether this study has been conducted properly and to assure the reliability of data arising from the study.	18.2.1 Objective of auditing<br>The objective of auditing is to determine whether this study has been conducted properly and to assure the reliability of data arising from the study. Audits will be conducted as the need arises.	Added descriptions on study conduct.
13	P67	18.2.2 Auditing committee<br>To audit this study, the Auditing Committee will be organized within the Japan Clinical Research Support Unit (J-CRSU).	18.2.2 Auditing committee<br>To audit this study, the Auditing Committee will be established at EP-CRSU Co., Ltd.	Changed the contracted auditor.
14	P69	20 Conflict of Interest and Source of Funding<br>This study will be funded by the CSPOR.	20 Conflict of Interest and Source of Funding<br>This study will be funded by the CSPOR.	Change to corporate entity.
15	P69	21 Clinical Study Registration<br>This study will be registered at the UMIN Clinical Trials Registry (UMIN-CTR; http://www.umin.ac.jp/ctr/index-j.htm) to make the study outline public. Registration will be made prior to enrollment of the first subject by the CSPOR Administrative Office.<br>The UMIN-CTR is a clinical trial registry which meets the international requirements for such registries. Need of registering clinical studies/trials has been an issue since the 1970s. In September 2004, the International Committee of Medical Journal Editors (ICMJE) announced its policy which required prior registration of a clinical study for the acceptance of an article about the study for publication in a medical journal,[40,41] which has led to the start of several clinical trial registry systems. The World Health Organization (WHO) has taken the initiative by launching the International Clinical Trials Registry Platform and establishing the requirements for registration[42] in April 2005. In the light of this international trend, the UMIN-CTR was launched in June 2005 as the first Japanese clinical trial registry. The UMIN-CTR has been recognized as an acceptable registry by the ICMJE and has also acted in concert with the WHO's clinical trial registry system.	21 Clinical Study Registration<br>This study will be registered at the UMIN Clinical Trials Registry (UMIN-CTR; http://www.umin.ac.jp/ctr/index-j.htm) to make the study outline public. Registration will be made prior to enrollment of the first subject by the CSPOR Administrative Office.<br>The UMIN-CTR is a clinical trial registry which meets the international requirements for such registries. Need of registering clinical studies/trials has been an issue since the 1970s. In September 2004, the International Committee of Medical Journal Editors (ICMJE) announced its policy which required prior registration of a clinical study for the acceptance of an article about the study for publication in a medical journal,[40,41] which has led to the start of several clinical trial registry systems. The World Health Organization (WHO) has taken the initiative by launching the International Clinical Trials Registry Platform and establishing the requirements for registration[42] in April 2005. In the light of this international trend, the UMIN-CTR was launched in June 2005 as the first Japanese clinical trial registry. The UMIN-CTR has been recognized as an acceptable registry by the ICMJE and has also acted in concert with the WHO's clinical trial registry system.<br>As of March 2019, the latest study-related information has been published on the Japan Registry of Clinical Trials (jRCT)–a web-based registry for public disclosure of clinical study protocols and summaries of study data. This registry was established to facilitate the submission of study protocols and other notifications to the Minister of Health, Labour and Welfare in accordance with the provisions of the "Clinical Trials Act" and the "Act on Securing Quality, Efficacy and Safety of Pharmaceuticals, Medical Devices, Regenerative and Cellular Therapy Products, Gene Therapy Products, and Cosmetics" (PMDA Act) concerning clinical research performed by medical institutions and other healthcare providers in order to ensure the disclosure of information required to be published by the World Health Organization (WHO) and to guarantee the transparency of study processes pursuant to the standards on clinical trial conduct stipulated in the above-mentioned legislation, as well as publishing relevant study information to assist potential volunteers in making informed choices regarding participation in national clinical studies.	Added descriptions on jRCT study registration and information disclosure following the shift to a "specified clinical study."

No.	Page No.	Original (version 2.1)	Revision (version 2.2)	Rationale & Description of Protocol Changes
16	p70	23. Study Organization and Administration<br>This is one of the clinical studies conducted under the sponsorship of the CSPOR.	23. Study Organization and Administration<br>This is one of the clinical studies conducted under the sponsorship of the Comprehensive Support Project for Oncology Research (CSPOR).	Change to corporate entity.
17	p70	23.1 N-SAS BC 06 executive committee<br>Chair (Principal Investigator)<br>Hiroji Iwata (Department of Breast Oncology, Aichi Cancer Center Hospital: Principal Investigator)<br>Steering Committee Member (in Kana order)<br>Masahiro Kashiwaba (Department of Surgery, Iwate Medical University)<br>Yutaka Yamamoto (Department of Breast and Endocrine Surgery, Kumamoto University Hospital)	23.1 N-SAS BC 06 executive committee<br>Chair (Principal Investigator)<br>Hiroji Iwata (Department of Breast Oncology, Aichi Cancer Center: Principal Investigator)<br>Steering Committee Members (in Kana order)<br>Masahiro Kashiwaba (Adachi Nyusen Clinic [Adachi Breast Clinic], Kyoto Breast Center, Medical Foundation Imai-kai Adachi Hospital)<br>Yutaka Yamamoto (Department of Breast and Endocrine Surgery, Kumamoto University Hospital)	Revised to show the most recent affiliations.
18	p70	23.1.1 N-SAS BC06 Imaging Evaluation Committee<br>Chair of Imaging Evaluation Committee<br>Hiroko Tsunoda (Department of Radiology, St Luke's International Hospital)<br>Committee Member of Imaging Evaluation<br>Ichiro Isomoto (Department of St. Francis Hospital)<br>Yukiko Tokuda (Department of NTT Osaka Hospital)<br>Kazuki Nakajima (Department of Breast and Thyroid Surgery, Kawasaki Medical School Hospital)<br>Shuichi Monzawa (Department of Radiology, Shinko Hospital)	(No text)	Deleted text to reflect changes in the CSPOR organizational structure.
19	p70	23.1.2 Advisor, Personnel<br>HOR: Shozo Ohsumi (Department of Breast Oncology, National Hospital Organization Shikoku Cancer Center)<br>TR: Hironobu Sasano (Department of Pathology, Tohoku University School of Medicine)<br>Shinichi Hayashi (Department of Medical Technology, Course of Health Science, School of Medicine, Tohoku University)<br>Central Pathological Review: Futoshi Akiyama (Department of Pathology, Japanese Foundation For Cancer Research)<br>Masafumi Kurosumi (Department of Pathology, Saitama Cancer Center)<br>Hitoshi Tsuda (Clinical Laboratory Division, National Cancer Center Central Hospital)	(No text)	Deleted text to reflect changes to the CSPOR organizational structure.
20	p71	23.2 Steering committee<br>Chair<br>Hirofumi Mukai (Department of Chemotherapy, National Cancer Center Hospital East)<br>Committee members<br>Hiroji Iwata (Department of Breast Oncology, Aichi Cancer Center Central Hospital)<br>Shozo Ohsumi (Department of Breast Oncology, National Hospital Organization Shikoku Cancer Center)<br>Shinji Ohno (Department of Breast Oncology, National Hospital Organization Kyushu Cancer Center)<br>Yasuo Hozumi (Department of Breast and General Surgery, Jichi Medical University Hospital)<br>Seiichiro Yamamoto (Center for Cancer Control and Information Service, National Cancer Center)	(No text)	Deleted text to reflect changes to the CSPOR organizational structure.
21	p71	23.3 Institutional Review Board<br>Chair<br>Shigeru Imoto (Department of Breast Surgery, School of Medicine, Kyorin University)<br>Vice chair<br>Tatsuya Toyama (Department of Breast and Endocrine Surgery, Nagoya City University Hospital)<br>Committee Member<br>Yukari Uemura (Graduate School of Medicine and Faculty of Medicine, The University of Tokyo)<br>Eishin Boku (Department of Surgery, Toho University Sakura Medical Center)	23.2 Institutional Review Board<br>Up to March 31, 2017:<br>Chair<br>Tatsuya Toyama (Department of Breast and Endocrine Surgery, Nagoya City University Hospital)<br>Vice chair<br>Eishin Boku (Department of Breast Surgery, Tohoku Medical and Pharmaceutical University Hospital)<br>Committee Members<br>Satoshi Teramukai (Study Statistician; Department of Biostatistics, Graduate School of Medicine, Kyoto Prefectural University of Medicine)<br>Nobuaki Matsubara (Department of Breast and Medical Oncology, National Cancer Center Hospital East)	Changes to CSPOR organizational structure.
22	p71	23.4 Independent Data Monitoring Committee<br>Committee member<br>Taro Shibata (National Cancer Center) (Center for Cancer Control and Information Service, National Cancer Center)<br>Akihiro Yanagisawa (CancerNet Japan, NPO)	23.3 Independent Data Monitoring Committee<br>Committee Members<br>Taro Shibata (Research Support Center, National Cancer Center)<br>Akihiro Yanagisawa (Medical Mobile Communications Co., Ltd.)	Revised to show the most recent affiliations.

No.	Page No.	Original (version 2.1)	Revision (version 2.2)	Rationale & Description of Protocol Changes
23	p71	23.5 Data management committee<br>Chair<br>Shozo Ohsumi (Department of Breast Oncology, National Hospital Organization Shikoku Cancer Center)<br>Committee member<br>Masataka Sawaki (Department of Breast Oncology, Aichi Cancer Center Hospital)<br>Naruto Taira (Department of Breast and Endocrine Surgery, Okayama University Graduate School of Medicine, Dentistry, and Pharmaceutical Sciences)	(No text)	Deleted text to reflect changes to the CSPOR organizational structure.
24	p71	23.6 CSPOR Data Center<br>Within the NPO, Japan Clinical Research Support Unit (J-CRSU)<br>Representative (Director of data center)<br>Yasuo Ohashi (Graduate School of Medicine and Faculty of Medicine, The University Tokyo)<br>School of Health Sciences and Nursing, Graduate School of Medicine, The University of Tokyo<br><br>204 Lions Plaza Ochanomizu<br>1-2-12 Yushima, Bunkyo-ku, Tokyo<br>113-0034, Japan<br>Phone: 03-3254-8029<br>FAX: 03-5298-8536<br>E-mail: support@csp.or.jp	23.4 Data Center (formerly CSPOR Data Center)<br>Up to March 31, 2020:<br>EP CRSU Co., Ltd. (formerly NPO Japan Clinical Research Support Unit [J-CRSU])<br>3F Acropolis Tokyo,<br>6-29 Shinogawa-machi, Shinjuku-ku, Tokyo 162-0814, Japan<br>Tel: 03-6759-9908<br>Fax: 03-5946-8274<br>Email: support@csp.or.jp<br><br>Up to April 1, 2020:<br>Data Management Office, General Affairs Department,<br>Comprehensive Support Project (CSP),<br>Public Health Research Foundation<br>1-1-7 Nishiwaseda, Shinjuku-ku, Tokyo 169-0051, Japan<br>Tel: 03-5287-2635<br>Fax: 03-5287-2644<br>Email: phrf-dm@csp.or.jp	Change of contracted data center.
25	p71	23.7 Office of Comprehensive Support Project for Oncological Research (CSPOR office)<br>Address: 3F, 1-1-7 Nishiwaseda, Shinjuku-ku Tokyo<br>169-0051, Japan<br>Public Health Research Foundation	23.5 Office of Comprehensive Support Project for Oncological Research (CSPOR office)<br>Address: 3F, 1-1-7 Nishiwaseda, Shinjuku-ku Tokyo 169-0051, Japan<br>Public Health Research Foundation	Change to corporate entity.

No.	Page No.	Original (version 1.2)	Amendment (version 2.1)	Rationale & Description of Protocol Changes
1	Header	N-SAS BC 06 version 1.2 March 25, 2011	N-SAS BC 06 version 2.1 June 1, 2012	Preparation of amended version
2	Cover page	CSPOR Data Center: Yasuo OHASHI,<br>Email: ohashi@epistat.m.u-tokyo.ac.jp	CSPOR Data Center: Yasuo OHASHI,<br>Email: support@csp.or.jp	Changed following revision of text.
3	Cover page	Approved by the Independent Monitoring Committee<br>(No text)	Approved by the Independent Data Monitoring Committee<br><br>Approved by the Independent Data Monitoring Committee: On May 31, 2012<br>Version 2.1 prepared: On June 1, 2012	"Independent Monitoring Committee" renamed as "Independent Data Monitoring Committee" due to change to CSPOR organization.<br><br>Specified the date on which the IDCM approved the protocol and the dates on which the amended versions of the protocol were prepared.
4	P4	(2) Exclusion criteria: Patients who have any of the following conditions will be excluded from the study.<br>4) Multiple primary invasive cancer diagnosed within 5 years after completion of previous cancer treatment	(2) Exclusion criteria: Patients who have any of the following conditions will be excluded from the study.<br>4) Multiple primary invasive cancer untreated or diagnosed within 5 years after completion of previous cancer treatment	Changed to reflect addition of "untreated multiple primary invasive cancer" to the exclusion criteria.
5	P6	0.6 Planned sample size and study duration<br>Planned sample size: A total of 1700 patients, 850 per group, are planned to be enrolled.<br>Enrollment: Until 5 years after enrollment of the first subject.<br>Follow-up: Until 5 years after enrollment of the last subject.<br>Study duration: Up to 10 years	0.6 Planned sample size and study duration<br>Planned sample size: A total of 850 patients, 425 per group, are planned to be enrolled.<br>Enrollment: Until 5 years after enrollment of the first subject.<br>Follow-up: Until 10 years after enrollment of the last subject.<br>Study duration: Up to 15 years	Modified due a change in the planned sample size and extension of the follow-up period.
6	p25	4.1.2 Exclusion criteria<br>Patients who have any of the following conditions, even if they meet all of the inclusion criteria, will not be included in the first part of the study:<br><br>4) Multiple primary invasive cancer diagnosed within 5 years after completion of previous cancer treatment	4.1.2 Exclusion criteria<br>Patients who have any of the following conditions, even if they meet all of the inclusion criteria, will not be included in the first part of the study:<br>4) Multiple primary invasive cancer untreated or diagnosed within 5 years after completion of previous cancer treatment	Changed to reflect addition of "untreated multiple primary invasive cancer" to the exclusion criteria.
7	p37	8.1 Screening for primary enrollment<br>14) HRQOL: Investigated at each center before sending the Primary Enrollment Form.	8.1 Screening for primary enrollment<br><br>14) *HRQOL: Investigated at each center before sending the Primary Enrollment Form.<br>*No HRQOL assessments are required in patients who were newly enrolled after December 15, 2011.	This text was added because the planned number of subjects undergoing the quality of life (QoL)/medical cost assessments reached 500 subjects on December 15, 2011.
8	p37	8.1.1 Filling in the Primary Enrollment Form<br>4) Whether the patient's HRQOL has been assessed.	8.1.1 Filling in the Primary Enrollment Form<br>4) *Whether the patient's HRQOL has been assessed.<br>*No HRQOL assessments are required in patients who were newly enrolled after December 15, 2011.	This text was added because the planned number of subjects undergoing the quality of life (QoL)/medical cost assessments reached 500 subjects on December 15, 2011.
9	p38	8.2 During the neoadjuvant protocol treatment<br><br>As the exceptions, HRQOL will be examined only at 1 and 4 months and pharmacoeconomic assessments will be made only at 4 months after the start of neoadjuvant LET therapy (see Section 8.9.1).<br><br>6) HRQOL: Only at 1 and 4 months after the start of treatment.<br>7) Pharmacoeconomic assessments (direct non-medical cost plus indirect cost): Only at 4 months after the start of treatment.	8.2 During the neoadjuvant protocol treatment<br><br>As the exceptions, *HRQOL will be examined only at 1 and 4 months and pharmacoeconomic assessments will be made only at 4 months after the start of neoadjuvant LET therapy (see Section 8.9.1).*<br><br>6) *HRQOL: Only at 1 and 4 months after the start of treatment.<br>7) *Pharmacoeconomic assessments (direct non-medical cost plus indirect cost): Only at 4 months after the start of treatment.<br>*No HRQOL assessments are required in patients who were newly enrolled after December 15, 2011.	This text was added because the planned number of subjects undergoing the quality of life (QoL)/medical cost assessments reached 500 subjects on December 15, 2011.
10	p39	8.4.2 At the start of the postoperative protocol treatment<br>10) HRQOL	8.4.2 At the start of the postoperative protocol treatment<br>10) *HRQOL<br>*No HRQOL assessments are required in patients who were newly enrolled after December 15, 2011.	This text was added because the planned number of subjects undergoing the quality of life (QoL)/medical cost assessments reached 500 subjects on December 15, 2011.

No.	Page No.	Original (version 1.2)	Amendment (version 2.1)	Rationale & Description of Protocol Changes
11	p40	8.5.1 During the first year As the exception, body weight and PS will be examined only at 6 months, while HRQOL and pharmacoeconomic assessments will be made only at 2 months after the start of the postoperative protocol treatment.<br><br>5) HRQOL<br>6) Pharmacoeconomic assessments (direct non-medical cost + indirect cost)	8.5.1 During the first year<br>As the exception, body weight and PS will be examined only at 6 months, while *HRQOL and *pharmacoeconomic assessments will be made only at 2 months after the start of the postoperative protocol treatment.<br><br>5) *HRQOL<br>6) *Pharmacoeconomic assessments (direct non-medical cost + indirect cost)<br>*No HRQOL assessments are required in patients who were newly enrolled after December 15, 2011.	This text was added because the planned number of subjects undergoing the quality of life (QoL)/medical cost assessments reached 500 subjects on December 15, 2011.
12	p40	8.5.2 During the second and subsequent years of the postoperative protocol treatment<br>As the exceptions, HRQOL and pharmacoeconomic assessments will be made only at 1 year after the start of the postoperative protocol treatment.<br>5) HRQOL<br>6) Pharmacoeconomic assessments (direct non-medical cost + indirect cost)	8.5.2 During the second and subsequent years of the postoperative protocol treatment<br>As the exceptions, *HRQOL and *pharmacoeconomic assessments will be made only at 1 year after the start of the postoperative protocol treatment.<br>5) *HRQOL<br>6) *Pharmacoeconomic assessments (direct non-medical cost + indirect cost)<br>*No HRQOL assessments are required in patients who were newly enrolled after December 15, 2011.	This text was added because the planned number of subjects undergoing the quality of life (QoL)/medical cost assessments reached 500 subjects on December 15, 2011.
13	p41	8.9.1 From primary enrollment until conclusion of the neoadjuvant protocol treatment<br>**See Section 12 "HRQOL and Pharmacoeconomic Assessments."	8.9.1 From primary enrollment until conclusion of the neoadjuvant protocol treatment<br>**No HRQOL assessments are required in patients who were newly enrolled after December 15, 2011.	This text was added because the planned number of subjects undergoing the quality of life (QoL)/medical cost assessments reached 500 subjects on December 15, 2011.
14	p42	8.9.2 From secondary enrollment until conclusion of the postoperative protocol treatment<br>**See Section 12 "HRQOL and Pharmacoeconomic Assessments."	8.9.2 From secondary enrollment until conclusion of the postoperative protocol treatment<br>**No HRQOL assessments are required in patients who were newly enrolled after December 15, 2011.	This text was added because the planned number of subjects undergoing the quality of life (QoL)/medical cost assessments reached 500 subjects on December 15, 2011.
15	p43	9.2 Monitoring of PD group patients<br>As the exceptions, HRQOL and pharmacoeconomic assessments will be made at 3 weeks after surgery (before the start of any subsequent treatment) and at 19 months after primary enrollment.<br>6) HRQOL and pharmacoeconomic assessments (direct non-medical cost + indirect cost)	9.2 Monitoring of PD group patients<br>As the exceptions, * HRQOL and *pharmacoeconomic assessments will be made at 3 weeks after surgery (before the start of any subsequent treatment) and at 19 months after primary enrollment.<br>6) *HRQOL and *pharmacoeconomic assessments (direct non-medical cost + indirect cost)<br>*No HRQOL assessments are required in patients who were newly enrolled after December 15, 2011.	This text was added because the planned number of subjects undergoing the quality of life (QoL)/medical cost assessments reached 500 subjects on December 15, 2011.
16	p44	9.4 Schedule of monitoring and assessment of patients with PD<br>**See Section 12 "HRQOL and Pharmacoeconomic Assessments."	9.4 Schedule of monitoring and assessment of patients with PD<br>**No HRQOL assessments are required in patients who were newly enrolled after December 15, 2011.	This text was added because the planned number of subjects undergoing the quality of life (QoL)/medical cost assessments reached 500 subjects on December 15, 2011.
17	p47	11.4.2 Reporting to the Independent Monitoring Committee<br>...will report this to the Independent Monitoring Committee in writing within 15 days after becoming aware of the event onset.	11.4.2 Reporting to the Independent Data Monitoring Committee<br>...will report this to the Independent Data Monitoring Committee in writing within 15 days after becoming aware of the event onset.	"Independent Monitoring Committee" renamed as "Independent Data Monitoring Committee" due to change to CSPOR organization.
18	p47	11.5 Review by the Independent Monitoring Committee<br>The Independent Monitoring Committee will review the adverse event report submitted by the principal investigator and will make written recommendations to the principal investigator about measures to be taken, including the handling of the specific subject and the suitability of continuing subject enrollment.	11.5 Review by the Independent Data Monitoring Committee<br>The Independent Data Monitoring Committee will review the adverse event report submitted by the principal investigator and will make written recommendations to the principal investigator about measures to be taken, including the handling of the specific subject and the suitability of continuing subject enrollment.	"Independent Monitoring Committee" renamed as "Independent Data Monitoring Committee" due to change to CSPOR organization.
19	p56	13.1 Central pathology review procedures and variables<br>In this study, central pathologists will review tumor tissue sections to determine the histological response to neoadjuvant endocrine therapy at surgery and the hormone receptor (ER and PgR) status at primary enrollment, changes in hormone receptor (ER and PgR) status and changes in HER2 protein expression status.<br>(No text)	13.1 Central pathology review procedures and variables<br>In this study, central pathologists will review tumor tissue sections to determine the histological response to neoadjuvant endocrine therapy at surgery and the hormone receptor (ER and PgR) status at primary enrollment, changes in hormone receptor (ER and PgR) status, changes in HER2 protein expression status and changes in Ki67 protein expression status.<br><br>Changes in Ki67 protein expression will be determined by analyzing the proportion of Ki67-positive cells (Ki labeling index) on immunostaining.	Changes in Ki67 protein expression was added as a central pathology review variable based on the assumption that many patients will have undergone Ki67 analysis in the course of their routine medical care.

No.	Page No.	Original (version 1.2)	Amendment (version 2.1)	Rationale & Description of Protocol Changes
20	p56	13.3 Pathological sections to be collected<br>Unstained and HE-stained specimens will be collected during pretreatment needle biopsy and surgery for patients with a histological response of Grade 1b, Grade 2 or Grade 3 as assessed at each center after neoadjuvant therapy with letrozole (section 3.4.2 "Criteria for rating histological response"; General Rules for Clinical and Pathological Recording of Breast Cancer [15th Edition, 2004]).	13.3 Pathological sections to be collected Unstained and HE-stained specimens will be collected during pretreatment needle biopsy and surgery in all patients, except those judged as PD for clinical response rating after neoadjuvant therapy with letrozole at each center.	Changed the assessment criteria for collected tissue specimens required for central pathology review.
21	p56	13.3.1 Sections for determining ER, PgR, and HER2 expression status<br>1) Four unstained sections of a needle biopsy specimen<br>2) Four unstained sections during surgery	13.3.1 Sections for determining ER, PgR, HER2 and Ki67 expression status<br>1) Five unstained sections of a needle biopsy specimen<br>2) Five unstained sections during surgery (specimens including representative sections of residual tumor)	Added the specimen to be used for evaluating Ki67 expression to the number of required specimens.<br>Modified the description to clarify the number of submitted specimens.
22	p56	13.3.2 Sections for evaluating histological response<br>2) HE-stained sections during surgery: All sections (representative sections)	13.3.2 Sections for evaluating histological response<br>2) One HE-stained section during surgery (specimens including representative sections of residual tumor)	Modified the description to clarify the number of submitted specimens.
23	p57	(No text)	13.6 Use of specimens in other studies<br>The patient's informed consent will again be needed if all or some of the specimens submitted are used for related studies of this study (translational research) other than the central pathological evaluation. The details will be specified in the protocol of each related study (translational research). Even in such cases, the institutional rules of each center or the judgment of the ethical committee should be followed.	Added because previous versions contained no mention of the use of specimens in related studies (translational research).
24	p58	(No text)	14.1 Purpose<br>Central imaging evaluation will be performed for the following purposes.<br>(1) To investigate the correlation of clinical response and pathological evaluation based on changes in the tumor images due to neoadjuvant endocrine therapy.<br>(2) To re-assess the tumor images by central review and determine the rate of concordance with each site's local pathology review.<br>(3) To standardize and improve the quality control of imaging procedures by investigating the imaging procedures at each study site.	Added because previous versions failed to mention the purpose(s) of central imaging evaluation.
25	p59	(No text)	14.6 Planned number of patients for imaging evaluation<br>Approximately 200 patients among the patients initially enrolled in the study will be included.	Added because previous versions did not specify the planned number of patients for imaging evaluation.
26	p62	16.1 Primary endpoint analyses and assessment criteria<br>If there is a large deviation from proportional hazards, similar analyses will be performed for 3- and 5-year cumulative survival rates.	16.1 Primary endpoint analyses and assessment criteria<br>If there is a large deviation from proportional hazards, similar analyses will be performed for 3-, 5-, 7- and 10-year cumulative survival rates.	Changed due to 5-year extension of the follow-up period.

No.	Page No.	Original (version 1.2)	Amendment (version 2.1)	Rationale & Description of Protocol Changes
27	p62	16.2 Rationale for planned sample size and enrollment duration A total of about 200 events (see Section 14) will be needed to provide a statistical power of 90% with these thresholds.<br><br>(No text)	16.2 Rationale for planned sample size and enrollment duration A total of about 200 events (see Section 14) will be needed to provide a statistical power of 90% (herein referred to as selection probability) with these thresholds.<br><br>Although this study was initiated in May 2008 based on the above justification, subject enrollment was actually unsuccessful. Thus, as a result of discussions by the Independent Data Monitoring Committee in March 2011, at 2 years and 10 months after the start of enrollment, approval was granted to extend the enrollment period from 3 years to 5 years and the study period from 8 years to 10 years. Subsequently, although the number of enrolled patients per month increased, it was impossible to reach the planned sample size of 1700 patients by the day of the end of enrollment (May 2013). Further extension of the enrollment period was deemed unfeasible given the importance of this study, and an extension of the follow-up period was subsequently proposed based on the opinion that the initial study objectives could be achieved by way of this extension, as discussed by the Principal Investigator, the Data Center representatives, and study statisticians at the Executive Committee meeting held in January 2012. Although the follow-up period was set as 5 years until protocol version 1.2, hormone receptor-positive breast cancer frequently relapses more than 5 years postoperatively [39]. Therefore, the follow-up period was extended from 5 to 10 years, and the long-term events were to be followed up, and the planned sample size was calculated again as follows. First, although the selection probability was set as 90% when the initial planned sample size was set, the initial purpose was almost reached, even with a selection probability of 80% to 85%. In such a case, approximately 170 events are required in both groups. Moreover, as described above, an overall number of approximately 630 patients are required when the 5 year DFS is assumed to be 88%, the enrollment duration and follow-up period to be 5 and 10 years, respectively (follow-up period will be up to 15 years). When approximately 1/4 of the initially enrolled patients are not assumed to be enrolled secondarily, approximately 850 patients in total are ultimately required. This number of patients can be enrolled in this study by May 2013 at the end of enrollment.	Revised due to recalculation of the planned sample size following the 5-year extension of the follow-up period.
28	p63	16.4 Secondary endpoint analyses<br>Confidence intervals around the 1-, 3- and 5- year survival rates will be constructed using Greenwood's formula.	16.4 Secondary endpoint analyses<br>Confidence intervals around the 1-, 3-, 5-, 7- and 10- year survival rates will be constructed using Greenwood's formula.	Changed due to 5-year extension of the follow-up period.
29	p64	16.7 Blinded monitoring<br>When it becomes necessary to change the protocol, the Committee will propose the change to the Clinical Study Subcommittee and the Independent Monitoring Committee. After the validity of the proposed change has been reviewed and approved by these committees, analyses will be performed under the changed protocol.	16.7 Blinded monitoring<br>When it becomes necessary to change the protocol, the Committee will propose the change to the Institutional Review Board (IRB) and the Independent Data Monitoring Committee (IDMC). After the validity of the proposed change has been reviewed and approved by the IRB and IDMC, analyses will be performed under the changed protocol.	In accordance with changes to CSPOR's organizational structure, the "Clinical Study Subcommittee" was changed to the "Institutional Review Board" and the "Independent Monitoring Committee" was changed to the "Independent Data Monitoring Committee."
30	p64	16.8 Interim analyses<br>The Independent Monitoring Committee will perform interim analyses and evaluate the antitumor effects of letrozole (LET) when the response to neoadjuvant therapy is determined in 140 patients to confirm the efficacy of neoadjuvant LET treatment.	16.8 Interim analyses<br>The Independent Data Monitoring Committee will perform interim analyses and evaluate the antitumor effects of letrozole (LET) when the response to neoadjuvant therapy is determined in 140 patients to confirm the efficacy of neoadjuvant LET treatment.	"Independent Monitoring Committee" renamed as "Independent Data Monitoring Committee" due to change to CSPOR organization.
31	p69	19. Publication of Study Findings<br>Study findings will be published according to the policies established by the Clinical Study Subcommittee.<br><br>The above-mentioned policies shall stipulate the rules for listing and ordering the names of authors who wish to publish any study findings, either by presentation at a scientific meeting or by submission of an academic journal manuscript. All materials must be reviewed and approved by the Clinical Trial Subcommittee and the Independent Monitoring Committee prior to publication.	19. Publication of Study Findings<br>Study findings will be published according to the policies established by the Institutional Review Board.<br><br>The above-mentioned policies shall stipulate the rules for listing and ordering the names of authors who wish to publish any study findings, either by presentation at a scientific meeting or by submission of an academic journal manuscript. All materials must be reviewed and approved by the Institutional Review Board and the Independent Data Monitoring Committee prior to publication.	In accordance with changes to CSPOR's organizational structure, the "Clinical Study Subcommittee" was changed to the "Institutional Review Board" and the "Independent Monitoring Committee" was changed to the "Independent Data Monitoring Committee."

No.	Page No.	Original (version 1.2)	Amendment (version 2.1)	Rationale & Description of Protocol Changes
32	p70	23.1 N-SAS BC 06 Executive Committee Executive Committee Members (listed in Japanese phonetic order)<br>Masahiro Kashiwaba (Department of Surgery, Iwate Medical University Hospital)<br>Shigehira Saji (Department of Clinical & Medical Oncology, Saitama Medical University International Medical Center)<br>Naruto Taira (Department of Breast and Endocrine Surgery, Okayama University Graduate School of Medicine, Dentistry, and Pharmaceutical Sciences)<br>Tatsuya Toyama (Department of Breast and Endocrine Surgery, Nagoya City University Hospital)<br>Norikazu Masuda (Department of Surgery, National Hospital Organization, Osaka National Hospital)<br>Yutaka Yamamoto (Department of Breast and Endocrine Surgery, Kumamoto University Hospital)<br>Imaging Evaluation Committee Members<br>Ichiro Isomoto (Department of Radiology, Nagasaki University Hospital)<br>Hiroko Tsunoda (Department of Radiology, St Luke's International Hospital)<br>Yukiko Tokuda (Department of Radiodiagnosis, National Hospital Organization, Osaka National Hospital)<br>Kazuki Nakajima (Department of Breast and Thyroid Surgery, Kawasaki Medical School Hospital)	23.1 N-SAS BC 06 Executive Committee Executive Committee Members (listed in Japanese phonetic order)<br>Shoichiro Ohtani (Department of Breast Surgery, Hiroshima City Hospital)<br>Masahiro Kashiwaba (Department of Surgery, Iwate Medical University Hospital)<br>Naruto Taira (Department of Breast and Endocrine Surgery, Okayama University Graduate School of Medicine, Dentistry, and Pharmaceutical Sciences)<br>Tatsuya Toyama (Department of Breast and Endocrine Surgery, Nagoya City University Hospital)<br>Tomomi Fujisawa (Department of Breast Oncology, Gunma Prefectural Cancer Center)<br>Norikazu Masuda (Department of Surgery, National Hospital Organization, Osaka National Hospital)<br>Yutaka Yamamoto (Department of Breast and Endocrine Surgery, Kumamoto University Hospital)<br><br>23.1.1 N-SAS BC06 Imaging Evaluation Committee Chair of Imaging Evaluation Committee<br>Hiroko Tsunoda (Department of Radiology, St Luke's International Hospital)<br>Imaging Evaluation Committee Members<br>Ichiro Isomoto (Department of Radiology, St. Francis Hospital)<br>Yukiko Tokuda (Department of Radiology, NTT West Osaka Hospital)<br>Kazuki Nakajima (Department of Breast and Thyroid Surgery, Kawasaki Medical School Hospital)<br>Shuichi Monzawa (Department of Radiology, Shinko Hospital)	Revised due to changes in the NEOS Executive Committee and Imaging Evaluation Committee members. Revised to show the most recent affiliations.
33	(p71)	23.1.1 N-SAS BC 06 Executive Committee mission	Deleted description.	Changed due to revision of text.
34	p71	23.2 Steering Committee<br>Steering Committee Chair<br>Yasuo Ohashi (Graduate School of Medicine, the University of Tokyo)<br><br>Steering Committee Vice Chair<br>Kojiro Shimozuma (Department of Biomedical Sciences, College of Life Sciences, Ritsumeikan University)<br><br>Steering Committee Members<br>Hiroji Iwata (Department of Breast Oncology, Central Hospital, Aichi Cancer Center Hospital)<br>Toru Watanabe (Hamamatsu Oncology Center)<br>Tadashi Ikeda (Department of Surgery, Teikyo University School of Medicine)<br>Masakazu Toi (Department of Breast Surgery, Kyoto University Hospital)<br>Takuji Iwase (Department of Breast Oncology, Ladies Center, Cancer Institute Ariake Hospital)<br>Yuichi Takatsuka (Department of Surgery, Kansai Rosai Hospital, Japan Organization of Health and Safety [JOHAS])<br>Shinzaburo Noguchi (Department of Breast and Endocrine Surgery, Osaka University Graduate School of Medicine)<br>Shinji Ono (Department of Breast Oncology, National Hospital Organization Kyushu Cancer Center)	23.2 Steering Committee<br>Steering Committee Chair<br>Hirofumi Mukai (Department of Chemotherapy, National Cancer Center Hospital East)<br><br>Steering Committee Members<br>Hiroji Iwata (Department of Breast Oncology, Central Hospital, Aichi Cancer Center Hospital)<br>Shozo Osumi (Department of Breast and Endocrine Surgery, National Hospital Organization Shikoku Cancer Center)<br>Shinji Ono (Department of Breast Oncology, National Hospital Organization Kyushu Cancer Center)<br>Yasuo Hozumi (Department of Breast and General Surgery, Jichi Medical University Hospital)<br>Seiichiro Yamamoto (Center for Cancer Control and Information Services, National Cancer Center)	Changes to Steering Committee member affiliations due to CSPOR organizational changes.
35	p71	23.2.1 Steering Committee mission	Deleted description.	Changed due to revision of text.
36	p71	23.3 Advisory Committee<br>23.3.1 Advisory Committee mission	Deleted description.	Deleted text to reflect changes to the CSPOR organizational structure.

No.	Page No.	Original (version 1.2)	Amendment (version 2.1)	Rationale & Description of Protocol Changes
37	p71	23.4 Clinical Study Subcommittee<br>Chair<br>Masakazu Toi (Department of Breast Surgery, Kyoto University Hospital)<br>Vice chair<br>Tetsuya Taguchi (Department of Endocrine and Breast Surgery, Kyoto Prefectural University of Medicine)<br>Committee members<br>Toru Watanabe (Hamamatsu Oncology Center)<br>Takuji Iwase (Department of Breast Oncology, Ladies Center, Cancer Institute Ariake Hospital)<br>Takuhiro Yamaguchi (Division of Biostatistics, Tohoku University Graduate School of Medicine)<br>Shinji Ohno (Department of Breast Oncology, National Hospital Organization Kyushu Cancer Center)<br>Hiroji Iwata (Department of Breast Oncology, Central Hospital, Aichi Cancer Center Hospital)<br>Observers<br>Yasuo Ohashi (Graduate School of Medicine, the University of Tokyo)<br>Kojiro Shimozuma (Department of Biomedical Sciences, College of Life Sciences, Ritsumeikan University)<br>Hirokuni Amari (Visiting Researcher, Public Health Research Foundation)	23.3 Institutional Review Board<br>Chair<br>Shigeru Imoto (Department of Breast Surgery, School of Medicine, Kyorin University)<br>Vice chair<br>Tatsuya Toyama (Department of Breast and Endocrine Surgery, Nagoya City University Hospital)<br><br>Committee members<br>Yukari Uemura (Graduate School of Medicine and Faculty of Medicine, The University of Tokyo)<br>Toshimi Takano (Department of Medical Oncology, Toranomon Hospital)<br>Kenji Tamura (Department of Breast and Medical Oncology, National Cancer Center Hospital Central Hospital)<br>Eishin Boku (Department of Surgery, Toho University Sakura Medical Center)	"Clinical Study Subcommittee" changed to "Institutional Review Board" due to change to CSPOR organizational structure. Changes to IRB members.
38	p71	23.4.1 Clinical Study Subcommittee mission	Deleted description.	Changed due to revision of text.
39	p71	23.5 Epidemiological Research Subcommittee<br>23.5.1 Epidemiological Research Subcommittee mission	Deleted description.	Deleted text to reflect changes to the CSPOR organizational structure.
40	p71	23.6 Education and Training Subcommittee<br>23.6.1 Education and Training Subcommittee mission	Deleted description.	Deleted text to reflect changes to the CSPOR organizational structure.
41	p71	23.7 Public Relations Subcommittee<br>23.7.1 Public Relations Subcommittee mission	Deleted description.	Deleted text to reflect changes to the CSPOR organizational structure.
42	p71	23.8 Independent Monitoring Committee<br><br>Chair<br>Tomoo Tajima (Department of Surgery, Breast Clinic, Tokyo Hospital, Tokai University School of Medicine)<br><br>Committee members<br>Suketami Tominaga (Honorary President, Aichi Cancer Center)<br>Fujio Kasumi (Breast Center, Juntendo University Hospital)<br>Eiko Uchida (NPO Bougainvillea)<br>Nobuo Seo (Attorney, Tokyo Hatchobori Law Office)	23.4 Independent Data Monitoring Committee<br><br>Chair<br>Seigo Nakamura (Department of Breast Surgery, Showa University Hospital)<br><br>Vice chair<br>Noriyuki Katsumata (Department of Medical Oncology, Nippon Medical School Musashikosugi Hospital)<br><br>Committee members<br>Taro Shibata (National Cancer Center) (Center for Cancer Control and Information Services, National Cancer Center)<br>Akihiro Yanagisawa (NPO Cancer Net Japan)	"Independent Monitoring Committee" renamed as "Independent Data Monitoring Committee" due to change to CSPOR organization.<br>Changes to Independent Data Monitoring Committee members.

No.	Page No.	Original (version 1.2)	Amendment (version 2.1)	Rationale & Description of Protocol Changes
43	p71	23.9 Data Management Committee<br><br>Chair<br>Yasuo Ohashi (Graduate School of Medicine, the University of Tokyo)<br><br>Committee members<br>Yuko Saito (Clinical Trial Coordination Office, Shizuoka Cancer Center)<br>Katsumasa Kuroi (Department of Breast Surgery/Department of Clinical Trials, Tokyo Metropolitan Cancer and Infectious Diseases Center Komagome Hospital)<br>Koji Oba (Graduate School of Medicine, Kyoto University)<br>Hiroshi Ohtsu (Department of Clinical Trial Data Management, Graduate School of Medicine, the University of Tokyo)<br>Harumi Kaba (National Cancer Center)<br>Shigeru Hayase (Japan Clinical Research Support Unit)<br>Naohito Fukui (Japan Clinical Research Support Unit)<br>Akio Ota (Japan Clinical Research Support Unit)	23.5 Data Management Committee<br><br>Chair<br>Shozo Osumi (Department of Breast and Endocrine Surgery, National Hospital Organization Shikoku Cancer Center)<br><br>Committee members<br>Masataka Sawaki (Department of Breast Oncology, Aichi Cancer Center Hospital)<br>Naruto Taira (Department of Breast and Endocrine Surgery, Okayama University Graduate School of Medicine, Dentistry, and Pharmaceutical Sciences)	Changes to Data Management Committee members.
44	p72	23.11 Office of Comprehensive Support Project for Oncology Research (CSPOR Office)<br>Manager<br>Hitoshi Masuda<br>Address: 3F, 1-7-7 Nishiwaseda, Shinjuku-ku, Tokyo 169-0051, Japan	23.7 Office of Comprehensive Support Project for Oncology Research (CSPOR Office)<br>Deleted description.<br>Address, 3F, 1-1-7 Nishiwaseda, Shinjuku-ku, Tokyo 169-0051, Japan	Changed due to revision of text.
45	p73	24.1.1 Classification of protocol changes<br>(1) Amendments<br>A protocol amendment must be reviewed and approved by the Independent Monitoring Committee and each center's ethics review committee.<br>The cover page of the amended protocol will bear the date of approval by the Independent Monitoring Committee.<br>(2) Revisions<br>A protocol revision need not be reviewed by the Independent Monitoring Committee but it must be approved by the Executive Committee Chair and reported to the Independent Monitoring Committee.	24.1.1 Classification of protocol changes<br>(1) Amendments<br>A protocol amendment must be reviewed and approved by the Independent Data Monitoring Committee and each center's ethics review committee. The cover page of the amended protocol will bear the date of approval by the Independent Data Monitoring Committee.<br>(2) Revisions<br>A protocol revision need not be reviewed by the Independent Data Monitoring Committee but it must be approved by the Executive Committee Chair and reported to the Independent Data Monitoring Committee.	"Independent Monitoring Committee" renamed as "Independent Data Monitoring Committee" due to change to CSPOR organization.
46	p73	24.1.2 Approval of protocol amendments/revisions by each center's ethics review committee<br>Any amendment to this protocol or written subject information made during the study and approved by the Independent Monitoring Committee must receive approval of each center's ethics review committee (or IRB) before implementation.	24.1.2 Approval of protocol amendments/revisions by each center's ethics review committee<br>Any amendment to this protocol or written subject information made during the study and approved by the Independent Data Monitoring Committee must receive approval of each center's ethics review committee (or IRB) before implementation.	"Independent Monitoring Committee" renamed as "Independent Data Monitoring Committee" due to change to CSPOR organization.
47	p73	24.2 Study termination<br>The Independent Monitoring Committee will review the appropriateness of continuing the study based on safety data and the results of interim analyses of efficacy.	24.2 Study termination<br>The Independent Data Monitoring Committee will review the appropriateness of continuing the study based on safety data and the results of interim analyses of efficacy.	"Independent Monitoring Committee" renamed as "Independent Data Monitoring Committee" due to change to CSPOR organization.

No.	Page No.	Original (version 1.2)	Amendment (version 2.1)	Rationale & Description of Protocol Changes
48	p77	26. REFERENCES<br>(No text)<br><br>39. Drummond MF, O'Brien B, Stoddart GL, Torrance GW. Methods for the economic evaluation of health care programmes. 2nd ed. New York: Oxford University Press; 1997.<br>40. International Committee of Medical Journal Editors. Uniform Requirements for Manuscripts Submitted to Biomedical Journals: Writing and Editing for Biomedical Publication. Updated February 2006. Available from: http://www.icmje.org/index.html.<br>41. De Angelis C, Drazen JM, Frizelle FA, Haug C, Hoey J, Horton R, et al.; International Committee of Medical Journal Editors. Clinical trial registration: a statement from the International Committee of Medical Journal Editors. N Engl J Med. 2004;351:1250-1.<br>42. World Health Organization. Technical Consultation on Trial Registration Standards; 2005 Apr 24–27; Geneva, Switzerland. Available from: http://www.who.int/ictrp/news/past_events/en/index.html.	26. REFERENCES<br>39. Saphner T, Tormey DC, Gray R. Annual hazard rates of recurrence for breast cancer after primary therapy. J Clin Oncol. 1996;14:2738-46.<br><br>40. Drummond MF, O'Brien B, Stoddart GL, Torrance GW. Methods for the economic evaluation of health care programmes. 2nd ed. New York: Oxford University Press; 1997.<br>41. International Committee of Medical Journal Editors. Uniform Requirements for Manuscripts Submitted to Biomedical Journals: Writing and Editing for Biomedical Publication. Updated February 2006. Available from: http://www.icmje.org/index.html.<br>42. De Angelis C, Drazen JM, Frizelle FA, Haug C, Hoey J, Horton R, et al.; International Committee of Medical Journal Editors. Clinical trial registration: a statement from the International Committee of Medical Journal Editors. N Engl J Med. 2004;351:1250-1.<br>43. World Health Organization. Technical Consultation on Trial Registration Standards; 2005 Apr 24–27; Geneva, Switzerland. Available from: http://www.who.int/ictrp/news/past_events/en/index.html.	Added literature references pertaining to Section 16.2 "Rationale for planned sample size and enrollment duration."<br><br>Changed numbering in References section due to addition of literature references.
49	Appendix A<br>Patient Information Sheet & Informed Consent Form<br>Cover page	(No text)	Version 2.1 prepared: On June 1, 2012	Preparation of amended version
50	Appendix A<br>Patient Information Sheet & Informed Consent Form<br>Header	N-SAS BC 06 Patient Information Sheet version 1.1 (110325)	N-SAS BC 06 Patient Information Sheet version 2.1 (120601)	Preparation of amended version
51	Appendix A<br>Patient Information Sheet & Informed Consent Form<br>P3	3. Study Methods<br>The entire study will continue for 10 years, and 1700 patients with the same condition are expected to take part in the study.	3. Study Methods<br>The entire study will continue for 15 years, and 850 patients with the same condition are expected to take part in the study.	Changed due to extension of study duration and change to the planned sample size.
52	Appendix A<br>Patient Information Sheet & Informed Consent Form<br>P3	3. Study Methods<br>Patients are expected to participate in the study for a minimum of 5 years and a maximum of 10 years.	3. Study Methods<br>Patients are expected to participate in the study for a minimum of 10 years and a maximum of 15 years.	Changed due to extension of follow-up period and study duration.
53	Appendix A<br>Patient Information Sheet & Informed Consent Form<br>P3	(Figure 1) Study Schedule<br>Observation period: 1 to 4 years	(Figure 1) Study Schedule<br>Observation period: At least 5 years	Changed due to extension of observation period.
54	Appendix A<br>Patient Information Sheet & Informed Consent Form<br>P 4	(Figure 2) Method for Random Assignment to Treatment<br>Number of patients: 1700<br>Number of patients treated with letrozole only: 850<br>Number of patients treated with letrozole after chemotherapy: 850	(Figure 2) Method for Random Assignment to Treatment<br>Number of patients: 850<br>Number of patients treated with letrozole only: 425<br>Number of patients treated with letrozole after chemotherapy: 425	Modified due to change in the planned sample size.
55	Appendix A<br>Patient Information Sheet & Informed Consent Form<br>P6	4. Survey on Quality of Life (QoL)	Deleted description.	This text was deleted from the Patient Information Sheet and Informed Consent Form because the planned number of subjects undergoing the quality of life (QoL) assessments reached 500 subjects on December 15, 2011.
56	Appendix A<br>Patient Information Sheet & Informed Consent Form<br>P6	5. Survey of Medical Costs	Deleted description.	This text was deleted from the Patient Information Sheet and Informed Consent Form because the planned number of subjects undergoing the medical cost assessment reached 500 subjects on December 15, 2011.

No.	Page No.	Original (version 1.2)	Amendment (version 2.1)	Rationale & Description of Protocol Changes
57	Appendix A<br>Patient Information Sheet & Informed Consent Form<br>P9	16. Study Organization and Review<br>Independent Monitoring Committee Chair: Tomoo Tajima, Tokai University School of Medicine	16. Study Organization and Review<br>Independent Data Monitoring Committee Chair: Seigo Nakamura, Showa University Hospital	"Independent Monitoring Committee" renamed as "Independent Data Monitoring Committee" due to change to CSPOR organization.<br>Change to the Independent Data Monitoring Committee Chair.
58	Appendix A<br>Patient Information Sheet & Informed Consent Form<br>P11, 12, 13	The planned study period is approximately 10 years, and a total of 1700 patients are expected to take part in the study.	Version 2.1 prepared: On June 1, 2012	Modified due to extension of study duration and change to the planned sample size.

N-SAS BC06 Protocol Revisions v1.2
Comparative List of Revisions

No.	Page No.	Original (version 1.1)	Revision (version 1.2)
1	Header	N-SAS BC 06 version 1.1 September 1, 2010	N-SAS BC 06 version 1.2 March 25, 2011
2	Cover page	(No text)	Version 1.2 prepared: On March 25, 2011
3	P6	0.6 Planned sample size and study duration<br>Planned sample size: A total of 1700 patients, 850 per group, are planned to be enrolled.<br>Enrollment: Until 3 years after enrollment of the first subject.<br>Follow-up: Until 5 years after enrollment of the first subject.<br>Study duration: Up to 8 years	0.6 Planned sample size and study duration<br>Planned sample size: A total of 1700 patients, 850 per group, are planned to be enrolled.<br>Enrollment: Until 5 years after enrollment of the first subject.<br>Follow-up: Until 5 years after enrollment of the last subject.<br>Study duration: Up to 10 years
4	Appendix A<br>Patient Information Sheet & Informed Consent Form<br>Cover page	(No text)	Version 1.2 prepared: On March 25, 2011
5	Appendix A<br>Patient Information Sheet & Informed Consent Form	N-SAS BC 06 Patient Information Sheet version 1.0 (080201)	N-SAS BC 06 Patient Information Sheet version 1.1 (110325)
6	Appendix A<br>Patient Information Sheet & Informed Consent Form	3. Study Methods<br>The entire study will continue for 8 years, and 1700 patients with the same condition are expected to take part in the study.	3. Study Methods<br>The entire study will continue for 10 years, and 1700 patients with the same condition are expected to take part in the study.
7	Appendix A<br>Patient Information Sheet & Informed Consent Form	3. Study Methods<br>Patients are expected to participate in the study for a minimum of 5 years and a maximum of 8 years.	3. Study Methods<br>Patients are expected to participate in the study for a minimum of 5 years and a maximum of 10 years.
8	Appendix A<br>Patient Information Sheet & Informed Consent Form<br>P11, 12, 13	The planned study period is approximately 8 years, and a total of 1700 patients are expected to take part in the study.	The planned study period is approximately 10 years, and a total of 1700 patients are expected to take part in the study.
9	Appendix D<br>Package Insert	(Revised in May 2007)	(Latest version: Replaced with revised version dated January 2010)
10	Appendix E<br>Helsinki Declaration		(Replaced with most recent version)

N-SAS BC06　Protocol Revisions v1.1
Comparative List of Revisions

No.	Page No.	Original (version 1.0)	Revision (version 1.1)
1	Header	N-SAS BC 06 version 1.0　February 1, 2008	N-SAS BC 06 version 1.1　September 1, 2010
2	P 1	(No text)	Version 1.1 prepared: On September 1, 2010
3	"	CSPOR Data Center: Yasuo OHASHI,<br>Department of Health Sciences and Nursing, Graduate School of Medicine, the University of Tokyo<br>5F, 1-2-13 Yushima, Bunkyo-ku, Tokyo, 113-0034, Japan	CSPOR Data Center: Yasuo OHASHI,<br>Department of Health Sciences and Nursing, Graduate School of Medicine, the University of Tokyo<br>204 Lions Plaza Ochanomizu<br>1-2-12 Yushima, Bunkyo-ku, Tokyo□113-0034, Japan
4	P 4	0.3　Eligibility criteria used for primary enrollment<br>(1) Inclusion criteria: Patients who meet all of the following criteria will be considered for enrollment.<br>•••••*(omitted)*•••••<br>4) HER2 score of ≤2+ on immunohistochemistry (IHC) of pretreatment (baseline) core needle biopsy specimen<br>However, if the result of a FISH assay is available at enrollment, this result must be negative.	0.3　Eligibility criteria used for primary enrollment<br>(1) Inclusion criteria: Patients who meet all of the following criteria will be considered for enrollment.<br>•••••*(omitted)*•••••<br>4) HER2 score of ≤2+ on immunohistochemistry (IHC) of pretreatment (baseline) core needle biopsy specimen,<br>or FISH-negative<br>(If the result of a FISH assay is available at enrollment, this result must be negative.)
5	P 5	0.4　Eligibility criteria used for secondary enrollment<br>(1) Inclusion criteria: Patients who meet all of the following criteria will be considered for inclusion in the randomized part of the study.<br>•••••*(omitted)*•••••<br>3) The following metastatic lymph node status is found after axillary lymph node dissection:<br>i) Patients with CR or PR: No lymph node metastasis (node negative), or metastasis positive (1 to 3 nodes involved)<br>ii) Patients with SD: No lymph node metastasis<br>However, a patient who does not undergo the lymph node dissection due to a negative sentinel lymph node biopsy will be considered as “node negative.”	0.4　Eligibility criteria used for secondary enrollment<br>(1) Inclusion criteria: Patients who meet all of the following criteria will be considered for inclusion in the randomized part of the study.<br>•••••*(omitted)*•••••<br>3) The following metastatic lymph node status is found after axillary lymph node dissection:<br>i) Patients with CR or PR: No lymph node metastasis (node negative), or metastasis positive (1 to 3 nodes involved)<br>ii) Patients with SD: No lymph node metastasis or metastasis positive (1 to 3 nodes involved), and the following criteria are met<br><br>• Nuclear grade ≤ Grade 2<br>• No widespread invasion of the vasculature surrounding the tumor<br>However, a patient who does not undergo the lymph node dissection due to a negative sentinel lymph node biopsy will be considered as “node negative.”

No.	Page No.	Original (version 1.0)	Revision (version 1.1)
6	P 24	4.1 Criteria for primary enrollment<br>4.1.1 Inclusion criteria<br>Patients who meet all of the following criteria will be considered for admission to the first part of the study:<br>•••••*(omitted)*•••••<br>4) HER2 score of ≤2+ on immunohistochemistry (IHC) of pretreatment (baseline) core needle biopsy specimen<br>However, if the result of a FISH assay is available at enrollment, this result must be negative.	4.1 Criteria for primary enrollment<br>4.1.1 Inclusion criteria<br>Patients who meet all of the following criteria will be considered for admission to the first part of the study:<br>•••••*(omitted)*•••••<br>4) HER2 score of ≤2+ on immunohistochemistry (IHC) of pretreatment (baseline) core needle biopsy specimen,<br>or FISH-negative<br>(If the result of a FISH assay is available at enrollment, this result must be negative.)
7	P 25	4.2 Criteria for secondary enrollment<br>4.2.1 Inclusion criteria<br>Patients who meet all of the following criteria will be considered for inclusion in the randomized part of the study:<br>•••••*(omitted)*•••••<br>3) The following metastatic lymph node status is found after axillary lymph node dissection:<br>i) Patients with CR or PR: No lymph node metastasis (node negative), or metastasis positive (1 to 3 nodes involved)<br>ii) Patients with SD: No lymph node metastasis<br>However, a patient who does not undergo the lymph node dissection due to a negative sentinel lymph node biopsy will be considered as “node negative.”	4.2 Criteria for secondary enrollment<br>4.2.1 Inclusion criteria<br>Patients who meet all of the following criteria will be considered for inclusion in the randomized part of the study:<br>•••••*(omitted)*•••••<br>3) The following metastatic lymph node status is found after axillary lymph node dissection:<br>i) Patients with CR or PR: No lymph node metastasis (node negative), or metastasis positive (1 to 3 nodes involved)<br>ii) Patients with SD: No lymph node metastasis or metastasis positive (1 to 3 nodes involved), and the following criteria are met<br><br>• Nuclear grade ≤ Grade 2<br>• No widespread invasion of the vasculature surrounding the tumor<br>However, a patient who does not undergo the lymph node dissection due to a negative sentinel lymph node biopsy will be considered as “node negative.”
8	P 37	8 Clinical and Laboratory Monitoring and Assessments<br>8.1 Screening for primary enrollment<br>•••••*(omitted)*•••••<br>6) Pathological examination for i) histological typing (see Section 3.2 Histological typing) and ii) histological grading (only for invasive carcinoma) (according to the N-SAS BC Criteria for histopathology grade, Appendix B)	8 Clinical and Laboratory Monitoring and Assessments<br>8.1 Screening for primary enrollment<br>•••••*(omitted)*•••••<br>6) Pathological examination for i) histological typing (see Section 3.2 Histological typing) and ii) histological grading (only for invasive carcinoma) (according to the N-SAS BC Criteria for histopathological grade)
9	P 37	8.1.1 Filling in the Primary Enrollment Form<br>1) Date of birth, the scheduled date of initiating postoperative treatments, height and body weight.	8.1.1 Filling in the Primary Enrollment Form<br>1) Date of birth, the scheduled date of initiating preoperative treatments, height and body weight.

No.	Page No.	Original (version 1.0)	Revision (version 1.1)
10	P 39	8.4 After surgery<br>8.4.1 At secondary screening: Filling in the Secondary Enrollment Form<br>1) Date of birth, date of surgery, scheduled date of initiating postoperative treatment, planned regimen for chemotherapy<br><br>2) That the patient meets the inclusion criteria with regard to:<br>Clinical response to the neoadjuvant protocol treatment<br>Whether sentinel lymph node biopsy has been performed or metastatic lymph node status has been examined after lymph node dissection<br>Postoperative pathological axillary node status and number of nodes involved	8.4 After surgery<br>8.4.1 At secondary screening: Filling in the Secondary Enrollment Form<br>1) Date of birth, date of surgery, scheduled date of initiating postoperative treatment<br>2) That the patient meets the inclusion criteria with regard to:<br>Clinical response to the neoadjuvant protocol treatment<br>Whether sentinel lymph node biopsy has been performed or metastatic lymph node status has been examined after lymph node dissection<br>Postoperative pathological axillary node status and number of nodes involved<br>Histological grade, presence of widespread invasion of vasculature surrounding the tumor (in case of SD only)
11	"	8.4 After surgery<br>8.4.1 At secondary screening: Filling in the Secondary Enrollment Form<br>•••••*(omitted)*•••••<br>3) That the patient does not meet any of the following included in the exclusion criteria:<br>HER2-positive (only if FISH assay results are available after primary enrollment)<br>Whether the patient's HRQOL has been assessed (investigated at each center before sending the Secondary Enrollment Form)	8.4 After surgery<br>8.4.1 At secondary screening: Filling in the Secondary Enrollment Form<br>•••••*(omitted)*•••••<br>3) That the patient does not have the following condition included in the exclusion criteria:<br>HER2-positive (only if FISH assay results are available after primary enrollment)<br><br>*Deleted text: *Whether the patient's HRQOL has been assessed (investigated at each*
12	"	8.4.2 At the start of the postoperative protocol treatment: Filling in the Postoperative Protocol Treatment Initiation Report	8.4.2 At the start of the postoperative protocol treatment<br><br>*Deleted text: *Filling in the Postoperative Protocol Treatment Initiation Report*
13	"	8.4.2 At the start of the postoperative protocol treatment: Filling in the Postoperative Protocol Treatment Initiation Report<br>•••••*(omitted)*•••••<br>9) Adverse events (assessed to obtain baseline data)	8.4.2 At the start of the postoperative protocol treatment<br>•••••*(omitted)*•••••<br>9) Adverse events (assessed to obtain baseline data)<br>10) HRQOL
14	P 44	9.4 Schedule of monitoring and assessment of patients with PD	9.4 Schedule of monitoring and assessment of patients with PD<br><br>(Added an arrow symbol to the *"Pharmacoeconomic assessments** (direct medical cost)"* column in the table)

No.	Page No.	Original (version 1.0)	Revision (version 1.1)
15	P 48	12.1.1 (2) Postoperative treatment period<br><br>Thus, the objectives of HRGOL assessments in this study will be:	12.1.1 (2) Postoperative treatment period<br><br>Thus, the objectives of <u>HRQOL</u> assessments in this study will be:
16	P 49	12.1.3 Schedule of assessments and acceptable time windows<br>In patients assigned to the CL or L groups, HRQOL will be assessed at the following 6 time points: at primary enrollment (after obtaining informed consent before sending the Primary Enrollment Form by fax), at 1 and 4 months after the start of the neoadjuvant protocol treatment, at secondary enrollment (before sending the Secondary Enrollment Form by fax), and at 2 and 12 months after the start of the postoperative protocol treatment.	12.1.3 Schedule of assessments and acceptable time windows<br>In patients assigned to the CL or L groups, HRQOL will be assessed at the following 6 time points: at primary enrollment (after obtaining informed consent before sending the Primary Enrollment Form by fax), at 1 and 4 months after the start of the neoadjuvant protocol treatment, at secondary enrollment (<u>after</u> sending the Secondary Enrollment Form by fax), and at 2 and 12 months after the start of the postoperative protocol treatment.
17	"	12.1.3 Schedule of assessments and acceptable time windows<br>•••••*(omitted)*•••••<br><br>2) During the postoperative protocol treatment At secondary enrollment: During the 2 weeks <u>before</u> sending the Secondary Enrollment Form by fax	12.1.3 Schedule of assessments and acceptable time windows<br>•••••*(omitted)*•••••<br><br>2) During the postoperative protocol treatment At secondary enrollment: During the 2 weeks <u>after</u> sending the Secondary Enrollment Form by fax
18	P 52	12.2.3 Methods<br>6) Presentation of results<br>CER represents the cost of obtaining obtaining 1 QALY...	12.2.3 Methods<br>6) Presentation of results<br>CER represents the cost of <u>obtaining</u> 1 QALY...

No.	Page No.	Original (version 1.0)	Revision (version 1.1)
19	P 56	(No text)	14. Central Imaging Evaluation<br>14.1 Procedure and items for central imaging evaluation<br>In this study, ultrasound images before neoadjuvant protocol treatment, during treatment, and after treatment, as well as MRI images before and after treatment will be collected in an electronic file, and central imaging evaluation will be performed. Internal rules/procedures defined by each submitting center should be followed when these images from each center are submitted.<br>14.2 Submission procedure of image data<br>Ultrasound and MRI images of enrolled patients will be converted to an electronic format such as JPEG, and be saved on a USB flash memory sent from the CSPOR Data Center before they are returned to the Center using a self-addressed envelope.<br>14.3 Imaging data to be collected<br>1) Ultrasound: Before neoadjuvant protocol treatment (at primary enrollment), at 1, 2, and 4 months after the start of neoadjuvant treatment, and at the end of the neoadjuvant protocol or at discontinuation.<br>2) MRI: Before neoadjuvant protocol treatment (at primary enrollment), and at the end of neoadjuvant protocol or at discontinuation. The minimum personal identification information whereby the imaging data submitted by each center can be linked to a certain enrolled patient will be attached to the imaging data file. The imaging data file will be saved with the minimum personal identification information, namely, the “center name” and the “subject identification number,” for the folder name and the “imaging type (US, MRI),” “subject identification number,” and “year and month (day) of imaging” for the file name. The name, initials, and/or chart number of patients should not be input in these data for submission.
	"	(No text)	14.4 Submission procedure of imaging data and place of submission<br>1) The Data Center will ask for the submission of imaging data and send a submission list and a USB flash memory for submission during the central imaging evaluation.<br>2) Create a folder by patient based on the submission list, name “center name” and “subject identification number” for the folder name and the “imaging type (US, MRI),” “subject identification number,” and “year and month (day) of imaging” for the file name, and save them on a USB flash memory at each center”.<br><br>Submission to: CSPOR Data Center<br>204 Lions Plaza Ochanomizu, 1-2-12 Yushima, Bunkyo-ku, Tokyo 113-0034, Japan<br>Tel: 03-3254-8029 Fax: 03-5298-8536

No.	Page No.	Original (version 1.0)	Revision (version 1.1)
20	P 60	15.8 Interim analyses The Independent Data Monitoring Committee (IDMC) will perform interim analysis at 1 year after the start of study enrollment and evaluate the antitumor effects of LET to confirm the efficacy of neoadjuvant LET treatment. If the proportion of patients with ≥SD is below 90%, the IDMC will make a recommendation to the Executive Committee to terminate the study or change the protocol. Interim analyses will also be performed at 1 and 2 years after the start of secondary enrollment to determine the patients' adherence to treatment and the incidence of adverse events. Interim analysis of efficacy in terms of DFS will be performed once the actual number of adverse events reaches 50% of the estimated incidence.	16.8 Interim analyses The Independent Data Monitoring Committee will perform interim analysis and evaluate the antitumor effects of letrozole (LET) when the response to neoadjuvant therapy is determined in 140 patients to confirm the efficacy of neoadjuvant LET treatment. When the number of enrolled patients is considered for the denominator and the hypothesis that the proportion of patients with ≥SD is below 75% is not rejected using a significance level of 5%, it is judged that neoadjuvant LET treatment is unlikely to obtain the expected effect, and a recommendation will be made to the Executive Committee to terminate the study or change the protocol. When the hypothesis is rejected, it is judged that neoadjuvant LET treatment is likely to obtain the expected effect, and the enrollment is continued. When the proportion of the threshold clinical response ≥ SD is 75%, and the proportion of the expected clinical response is 85% (corresponding to 80% and 90%, respectively, when evaluable patients are considered as the denominator) with error α 0.05 and error ß 0.1, approximately 140 patients are required based on binomial distribution.
21	P66-73	22. Study Organization and Administration	22. Study Organization and Administration<br><br>(Updated affiliations, addresses and other details of the study organization)
22	P66	22.1 N-SAS BC 06 executive committee	22.1 N-SAS BC 06 executive committee<br>•••••*(omitted)*•••••<br>Imaging Evaluation Committee Members<br>Ichiro Isomoto (Department of Radiology, Nagasaki University Hospital)<br>Hiroko Tsunoda (Department of Radiology, St Luke's International Hospital)<br>Kazuki Nakajima (Department of Breast and Thyroid Surgery, Kawasaki Medical School Hospital)<br>Yukiko Tokuda (Department of Radiodiagnosis, National Hospital Organization, Osaka National Hospital)

No	NEOS: N-SAS BC06 Protocol v1.1 Rationale & Description of Protocol Revisions
1	Preparation of revised version
2	Preparation of revised version
3	Preparation of revised version
4	Revised because some study sites did not perform immunohistochemistry (IHC) for the assessment of HER2 status, and only performed the FISH assay.
5	According to the eligibility criteria for secondary enrollment, a considerable number of SD patients were reported as ineligible for secondary enrollment, and many of these patients were deemed ineligible due to a finding of "lymph node metastasis positive after axillary lymph node dissection."<br>Therefore, in accordance with the international consensus on postoperative breast cancer therapy in patients among the target population of the present study (St. Gallen, 2009)*, the eligibility criteria for SD patients was modified to enable secondary enrollment of patients with "*factors not useful for decision*" (on postoperative adjuvant chemotherapy)" and patients without "*relative indications for endocrine chemotherapy*" (i.e., patients in whom there is no clear-cut rationale for considering chemotherapy).<br><br>Among the "*factors not useful for decision*" (on postoperative adjuvant chemotherapy)," clinical laboratory assessments not used in general practice in Japan and multigene assays/gene signature (e.g., Oncotype DX, MammaPrint) and proliferation (Ki-67, etc.) tests not covered under the National Health Insurance (NHI) scheme were not used for the eligibility criteria.<br><br>*Annals of Oncology 20: 1319–1329, 2009
6	As described in No. 4.
7	As described in No. 5.
8	The reference to Appendix B was a typographical error and was therefore deleted.
9	Revised due to typographical error.
10	The description was modified due to the revision concerning secondary enrollment. See No. 5.
11	HRQOL was assessed *after* rather than *before* sending the Secondary Enrollment Form so this text was deleted and moved to item 10) in section 8.4.2.
12	This description was deleted because it included an assessment (HRQOL) that is not specified in the Postoperative Protocol Treatment Initiation Report.
13	See No. 11.
14	Revised to include the previously-omitted arrow symbol.
15	Revised because this assessment is performed *after* rather than *before* sending the Secondary Enrollment Form. See No. 9.
16	Revised because this assessment is performed *after* rather than *before* sending the Secondary Enrollment Form. See Nos. 9 and 13.
17	Revised due to typographical error.
18	Revised due to typographical error.

19	This description was added because imaging data will be centrally evaluated in this study.
20	The text was revised to provide specific details reflecting the progress of the study.
21	The text was revised to show the most recent affiliations of the committee members.
22	The Imaging Evaluation Committee was established so the names of the committee members were listed.
