# Supplementary material for: Phase III study of long-term prognosis of estrogen receptor-positive early breast cancer treated with neoadjuvant endocrine therapy with/without adjuvant chemotherapy
Source: Breast Cancer Res Treat. 2023 Mar 22;199(2):231–41. doi: 10.1007/s10549-023-06874-7 (PMC10175450; doi:10.1007/s10549-023-06874-7)
Supplement: Supplementary file 2 — Supplementary file2 (PDF 358 KB) [file 10549_2023_6874_MOESM2_ESM.pdf]

Supplementary Information

Phase III study of long-term prognosis of estrogen receptor-positive early breast cancer treated with neoadjuvant endocrine therapy with/without adjuvant chemotherapy

Hiroji Iwata[1], Yutaka Yamamoto[2], Takehiko Sakai[3], Yoshie Hasegawa[4], Rikiya Nakamura[5], Hiromitsu Akabane[6], Shoichiro Ohtani[7], Masahiro Kashiwaba[8], Naruto Taira[9], Tatsuya Toyama[10], Tomomi Fujisawa[11], Norikazu Masuda[12], Yukiko Shibahara[13,#], Hironobu Sasano[13], Takuhiro Yamaguchi[13]

[1]Aichi Cancer Center Hospital, 1-1 Kanokoden, Chikusa-ku, Nagoya 464-8681, Japan
[2]Kumamoto University Hospital, 1-1-1 Honjo, Chuo-ku, Kumamoto 860-8556, Japan
[3]Cancer Institute Hospital of Japanese Foundation for Cancer Research, 38-31 Ariake, Koto, Tokyo 135-8550, Japan
[4]Hachinohe City Hospital, 3-1-1 Tamukai, Hachinohe 031-8555, Japan
[5]Chiba Cancer Center, 666-2 Nitona-cho, Chuo-ku, Chiba 260-8717, Japan
[6]Hokkaido P.W.F.A.C. Asahikawa-Kosei General Hospital, 1-24-111 Asahikawa 078-8211, Japan
[7]Hiroshima City Hiroshima Citizens Hospital, 7-33 Motomachi, Naka-ku, Hiroshima 730-8518, Japan
[8]Adachi Breast Clinic, 98 Kamigamo Matsumoto-cho, Kita-ku, Kyoto 603-8052, Japan [9]Kawasaki Medical School, 577 Matsushima, Kurashiki, Okayama 701-0192, Japan
[10]Nagoya City University Graduate School of Medical Sciences, 1 Kawasumi, Mizuho-cho, Mizuho-ku, Nagoya 467-8601, Japan
[11]Gunma Prefectural Cancer Center, 617-1 Takahayashinishi-cho, Ota, Gunma 373-8550, Japan
[12]Nagoya University Graduate School of Medicine, 65 Tsurumai-cho, Showa-ku, Nagoya 466-8550, Japan
[13]Tohoku University School of Medicine, 2-1 Seiryo-machi, Aoba-ku, Sendai, Miyagi 980-8575, Japan

[#]Current affiliation:
Kitasato University, 1-15-1 Kitazato, Minami-ku, Sagamihara, Kanagawa 252-0373, Japan

Correspondence to: Hiroji Iwata, Breast Oncology, Aichi Cancer Center Hospital, 1-1 Kanokoden, Chikusa-ku, Nagoya 464-8681, Japan. Email: hiwata@aichi-cc.jp

Supplementary Figure S1

A

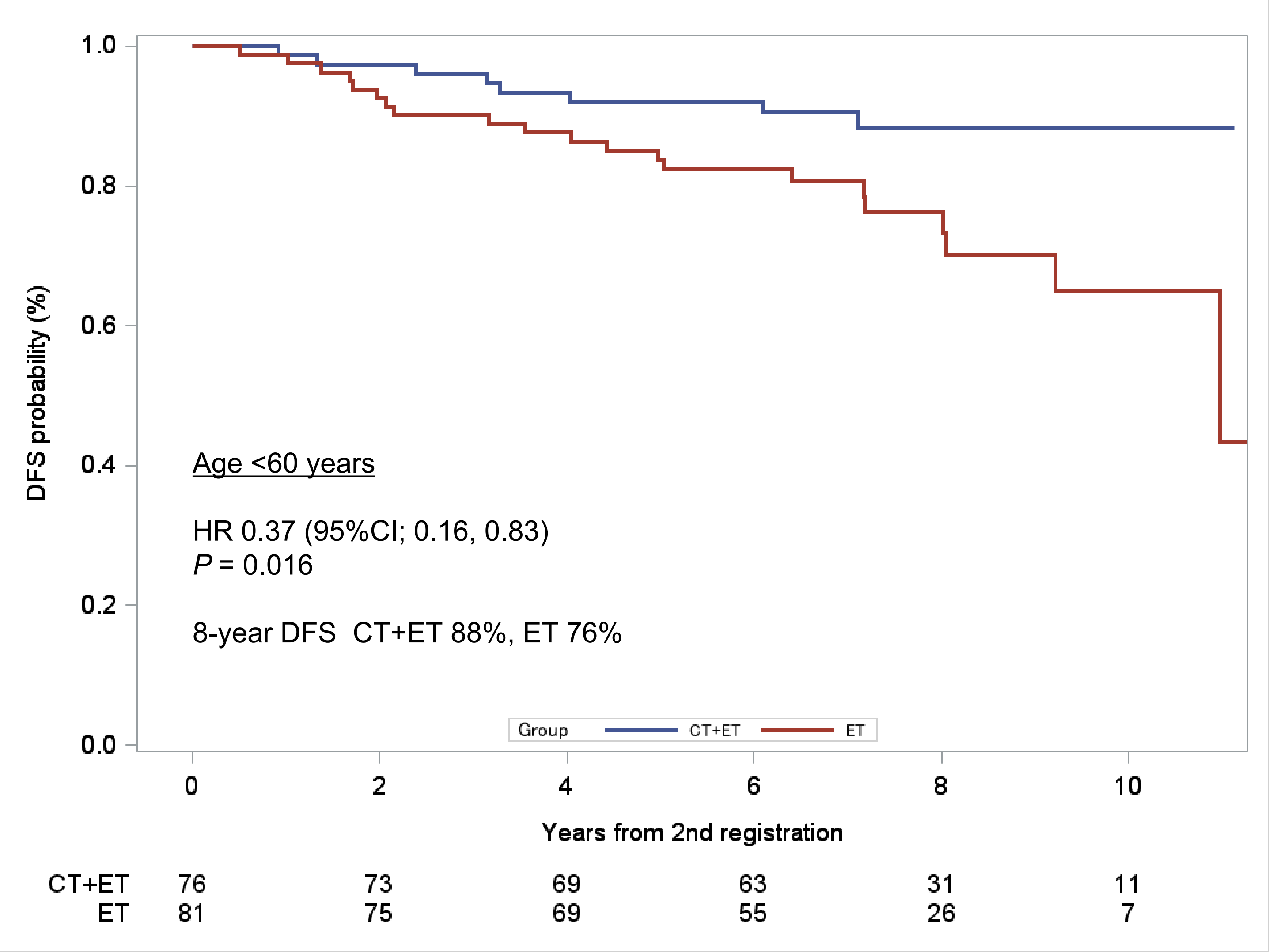

B

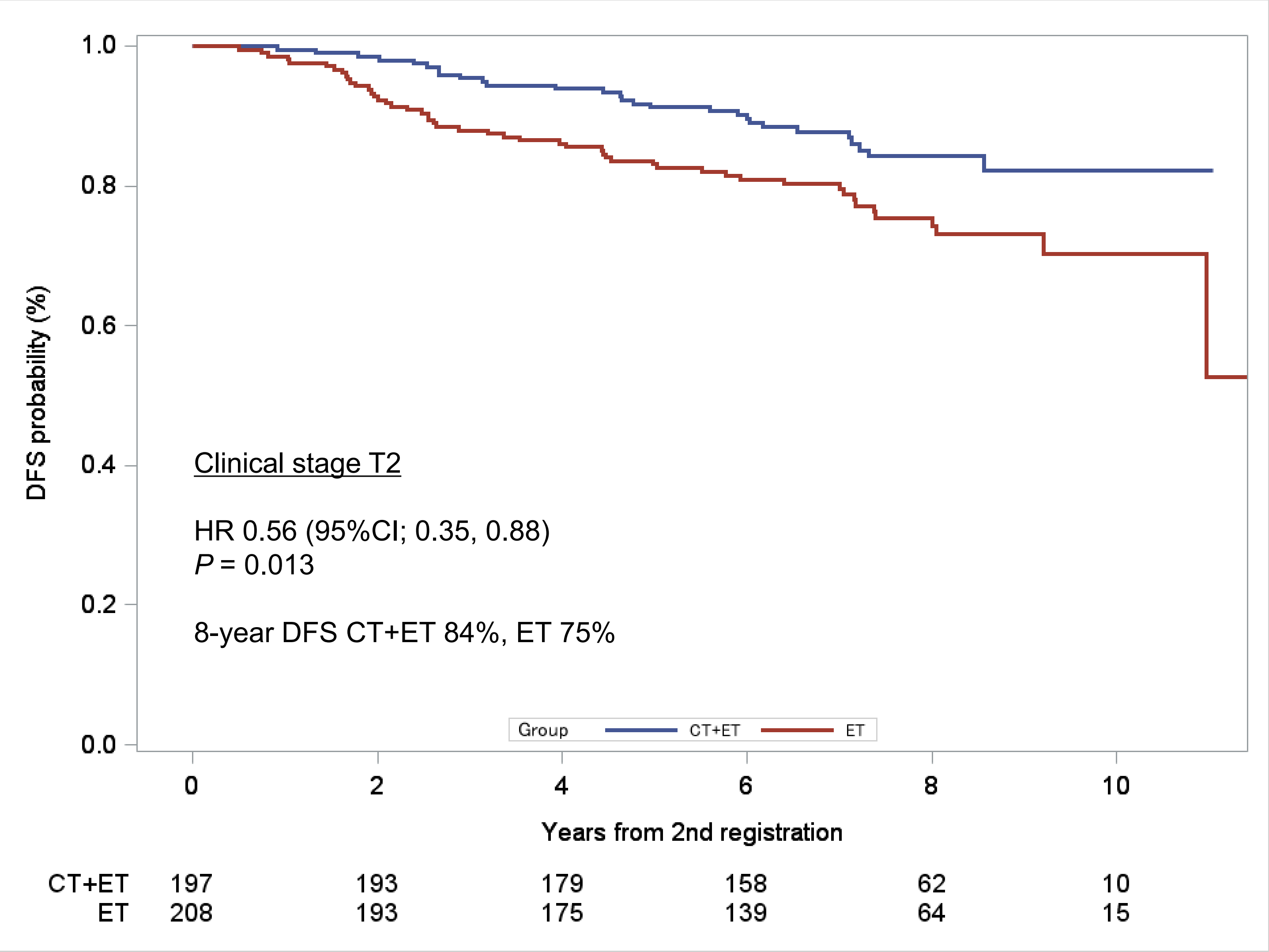
1.0
0.8
0.6
0.4
0.2
0.0
DFS probability (%)
Clinical stage T2
HR 0.56 (95%CI; 0.35, 0.88)
P = 0.013
8-year DFS CT+ET 84%, ET 75%
Group
CT+ET
ET
0
2
4
6
8
10
Years from 2nd registration
CT+ET 197 193 179 158 62 10
ET 208 193 175 139 64 15

C

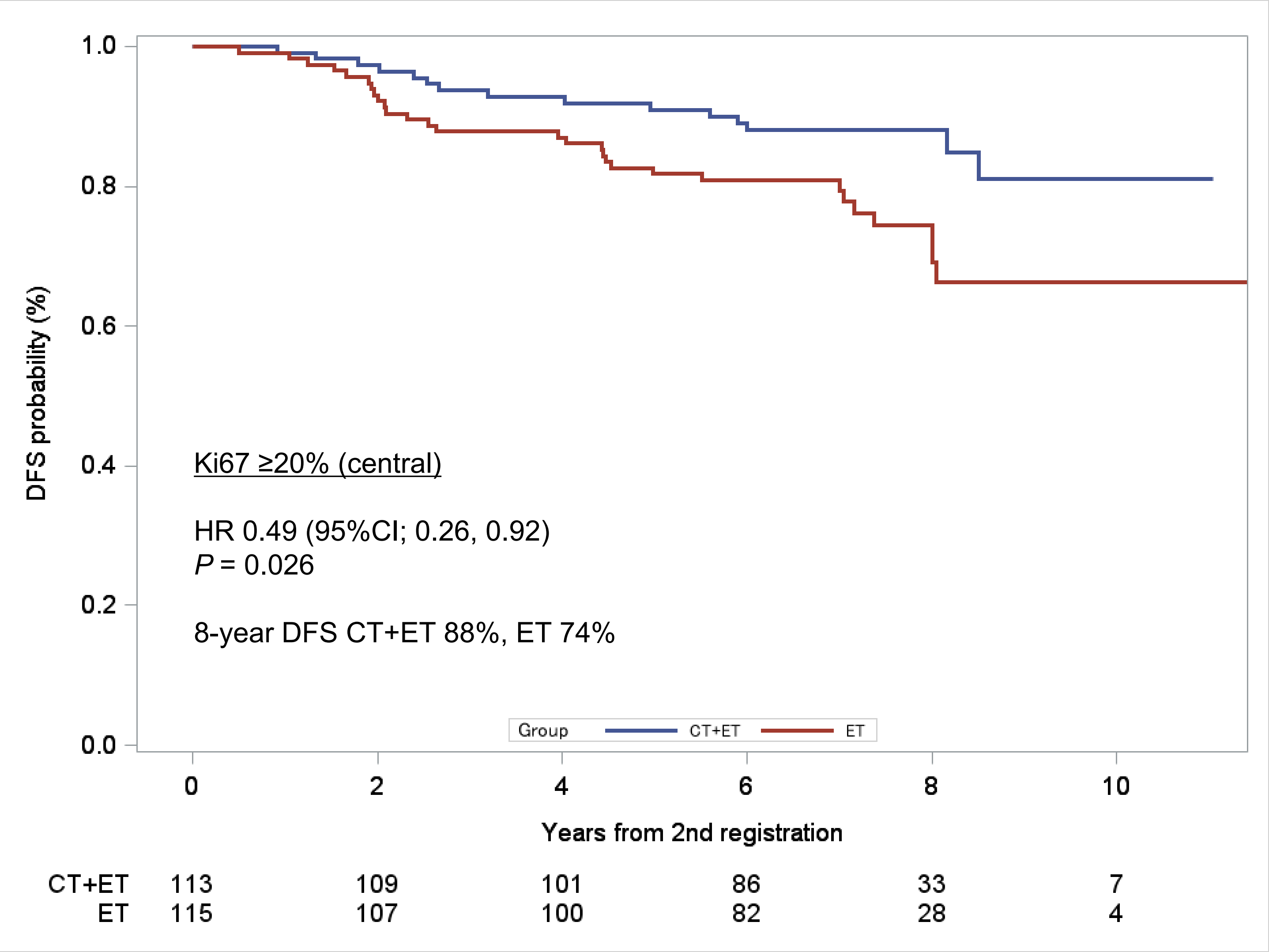
1.0
0.8
0.6
0.4
0.2
0.0
DFS probability (%)
Ki67 ≥20% (central)
HR 0.49 (95%CI; 0.26, 0.92)
P = 0.026
8-year DFS CT+ET 88%, ET 74%
Group
CT+ET
ET
0
2
4
6
8
10
Years from 2nd registration
CT+ET 113 109 101 86 33 7
ET 115 107 100 82 28 4

Supplementary Figure S2

A

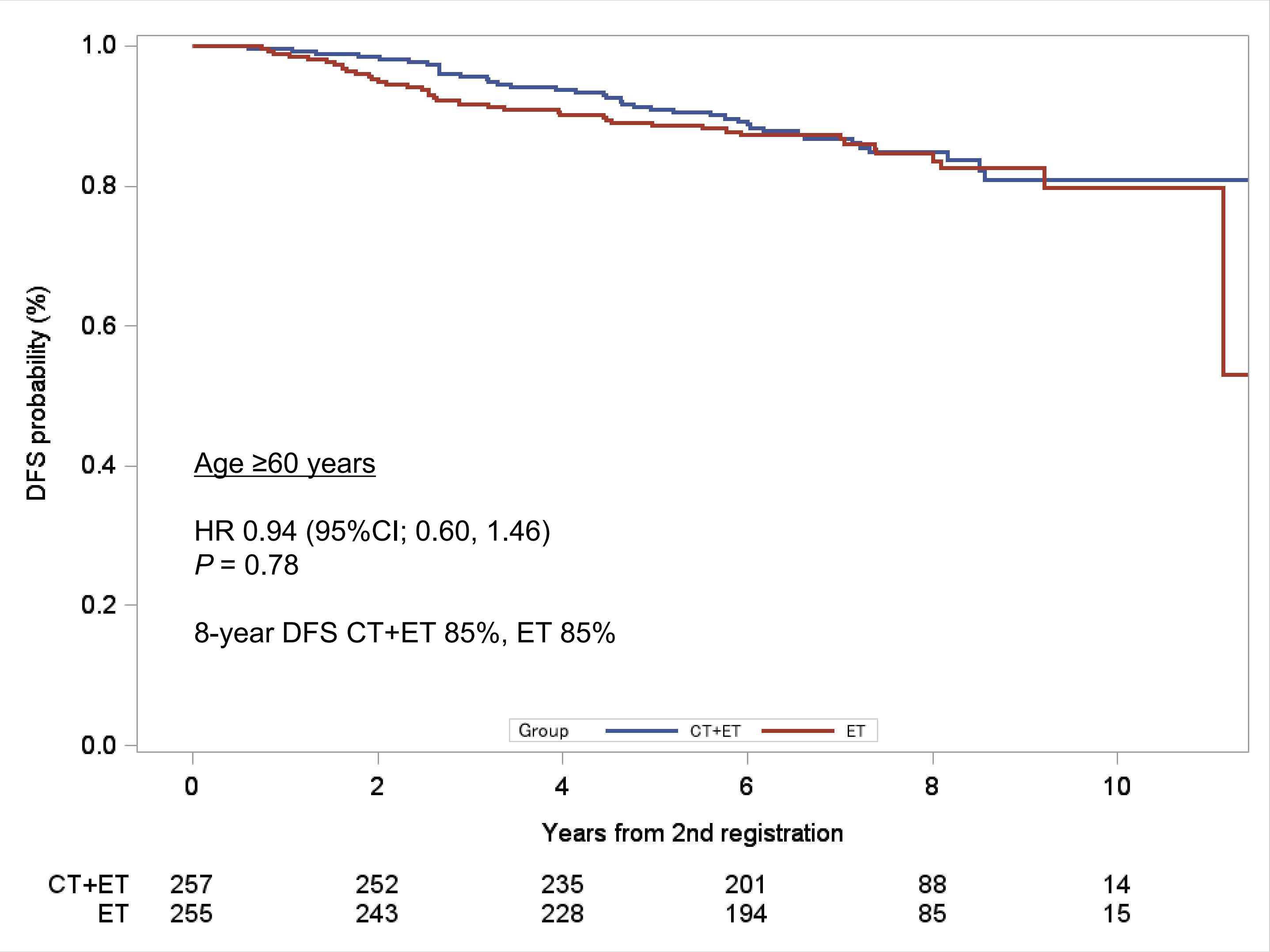

B

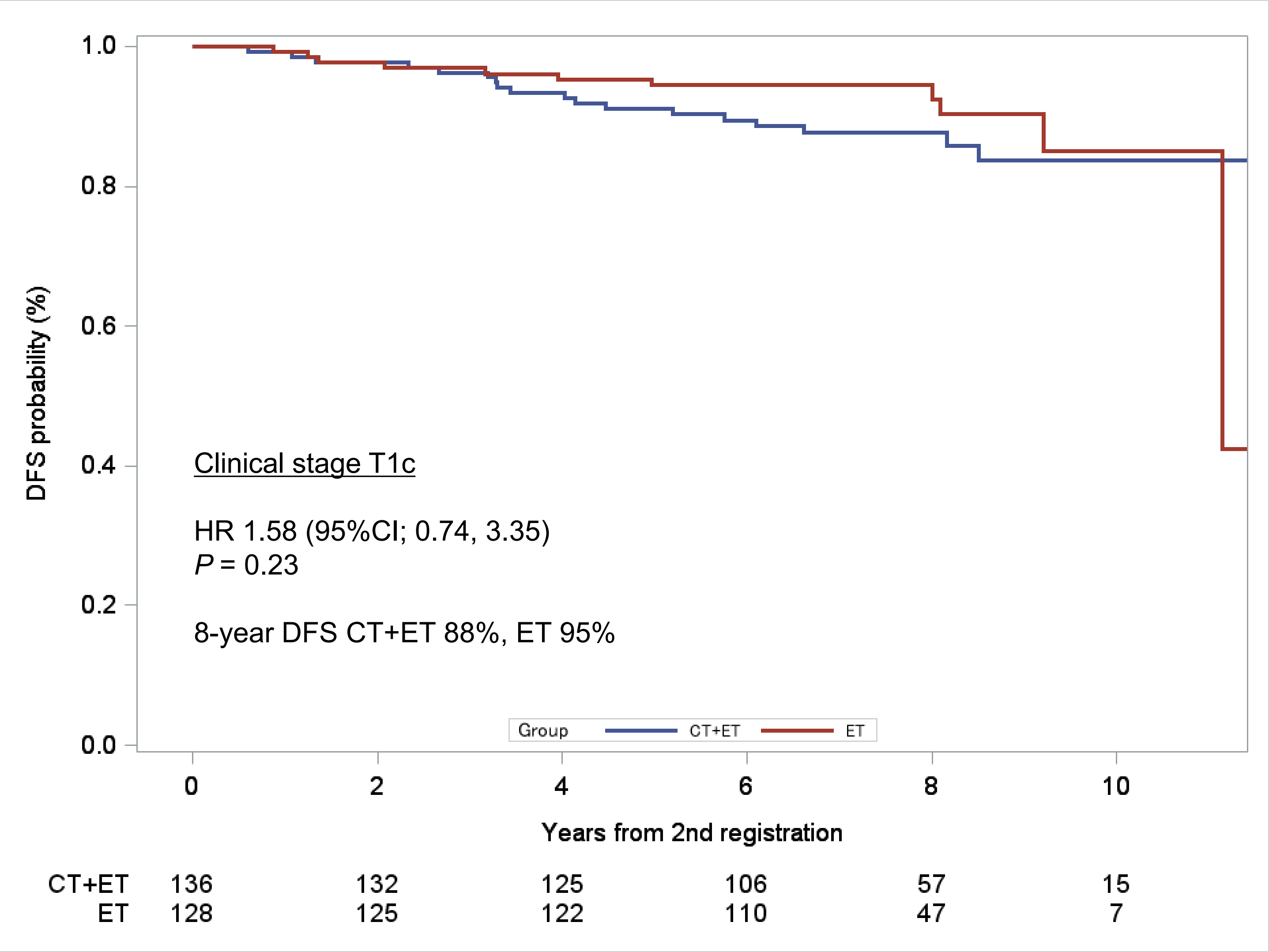

DFS probability (%)
1.0
0.8
0.6
0.4
0.2
0.0
Clinical stage T1c
HR 1.58 (95%CI; 0.74, 3.35)
P = 0.23
8-year DFS CT+ET 88%, ET 95%
Group CT+ET ET
0
2
4
6
8
10
Years from 2nd registration
CT+ET 136 132 125 106 57 15
ET 128 125 122 110 47 7

C

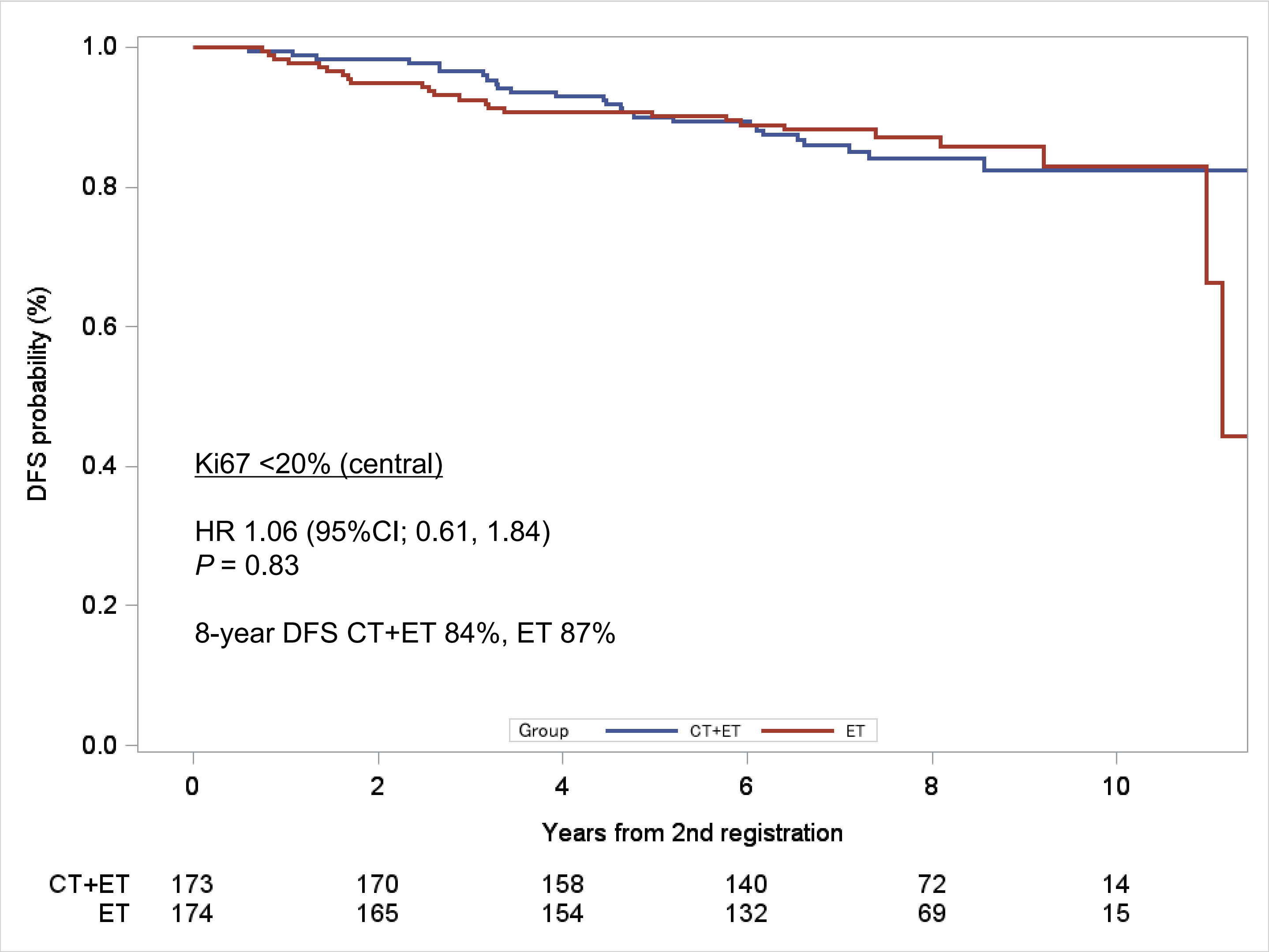
1.0
0.8
0.6
0.4
0.2
0.0
DFS probability (%)
Ki67 <20% (central)
HR 1.06 (95%CI; 0.61, 1.84)
P = 0.83
8-year DFS CT+ET 84%, ET 87%
Group
CT+ET
ET
0
2
4
6
8
10
Years from 2nd registration
CT+ET 173 170 158 140 72 14
ET 174 165 154 132 69 15

Supplementary Figure S3

A

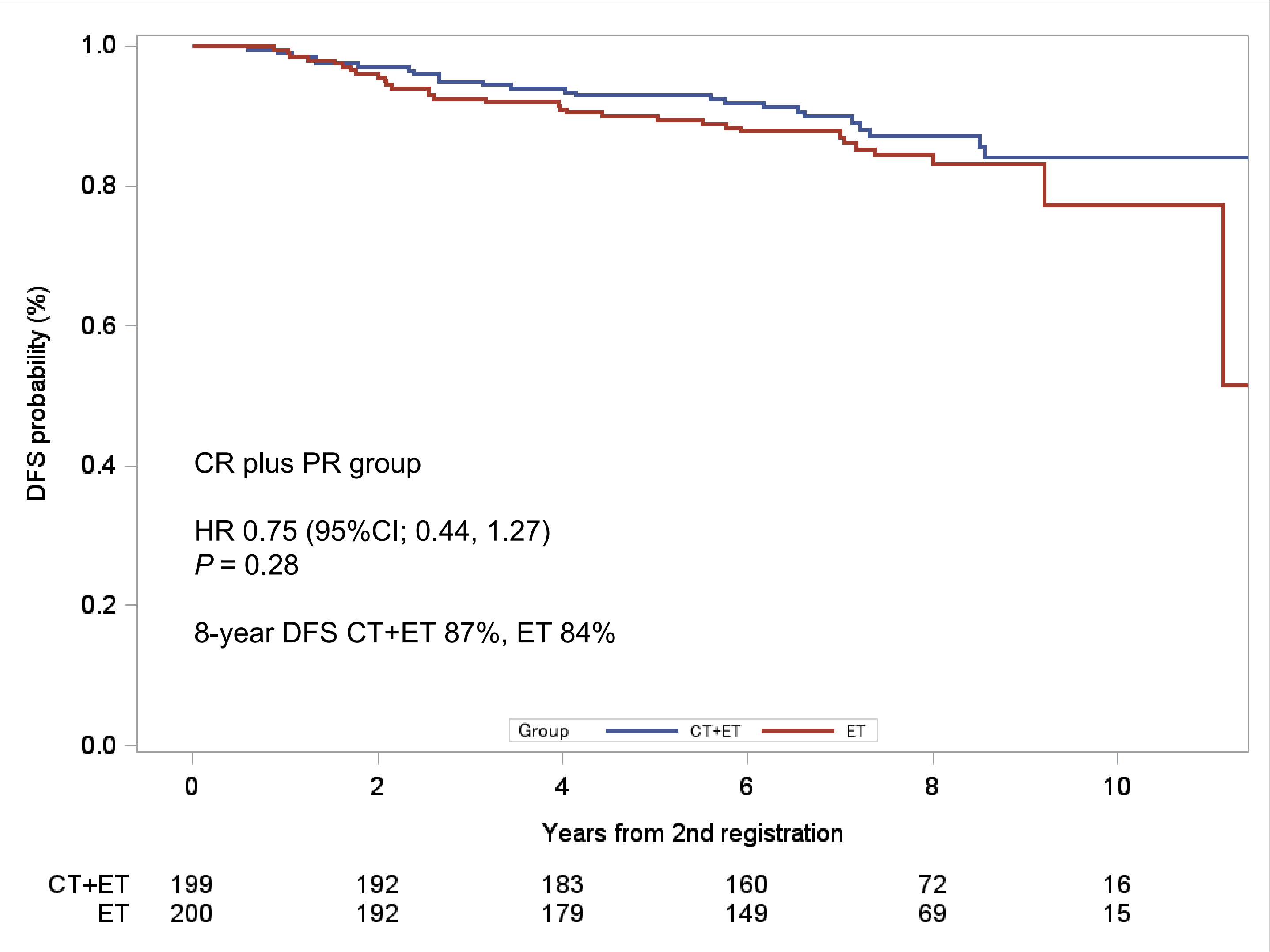

B

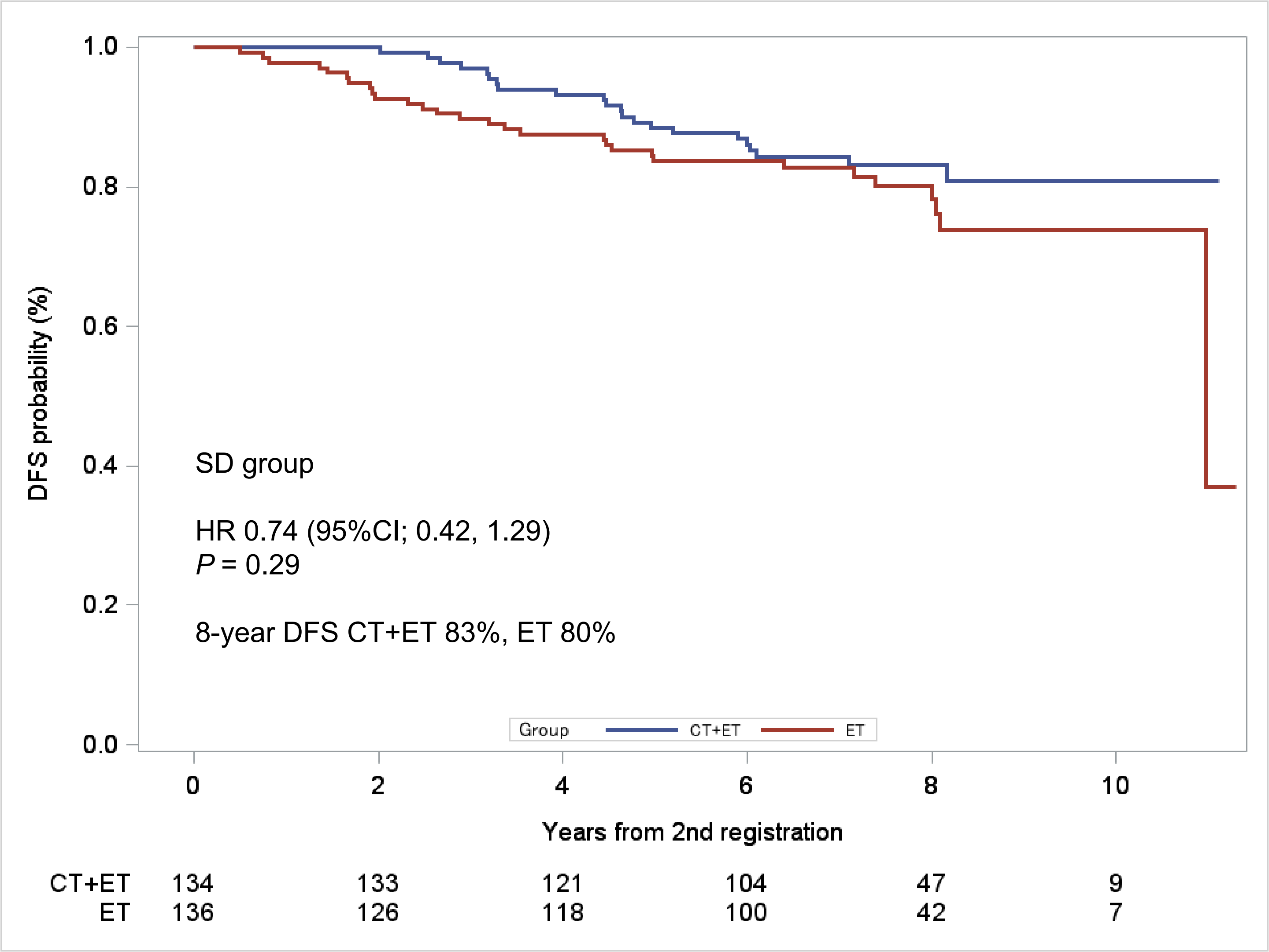

DFS probability (%)
1.0
0.8
0.6
0.4
0.2
0.0
SD group
HR 0.74 (95%CI; 0.42, 1.29)
P = 0.29
8-year DFS CT+ET 83%, ET 80%
Group
CT+ET
ET
0
2
4
6
8
10
Years from 2nd registration
CT+ET 134 133 121 104 47 9
ET 136 126 118 100 42 7

Supplementary Table S1. Chemotherapy regimens in CT + ET group

	**CT+ET (patients)**
AC/EC	32
FAC/FEC	19
AC/EC/FEC-T	8
TC	144
CMF	63
No chemo	67

AC/EC; adriamycin or epirubicin in combination with cyclophosphamide; CMF, cyclophosphamide, methotrexate, and 5-Fluorouracil; CT, chemotherapy; ET, endocrine therapy; FAC, 5-fluorouracil, adriamycin, and cyclophosphamide; FEC, 5-fluorouracil, epirubicin, and cyclophosphamide; FEC-T, Fluorouracil, epirubicin, cyclophosphamide and docetaxel; TC, docetaxel and cyclophosphamide.

Supplementary Table S2. Predictive markers of clinical response to letrozole

	Odds ratio	(95% CI)	*P* value
Age ≥65 vs. <65 years	0.72	(0.32–1.63)	0.43
BMI ≥25 vs. <25 kg/m^2	0.45	(0.17–1.20)	0.11
T2 vs. T1c	0.73	(0.31–1.73)	0.48
HG 2 vs. 1	1.98	(0.56–6.95)	0.29
HG 3 vs. 1	10.84	(2.23–52.72)	0.0031
Progesterone receptor positive vs. negative	0.23	(0.10–0.50)	0.0002
HER2 1+ vs. 0 (IHC, local)	0.70	(0.29–1.69)	0.43
HER2 2+ vs. 0 (IHC, local)	0.99	(0.35–2.79)	0.99
Ki67 ≥20% vs. <20% (central)	1.82	(0.79–4.18)	0.16

BMI, body mass index; CI, confidence interval; HG, histological grade; HER2, human epidermal growth factor receptor 2; IHC, immunohistochemistry.
