# Supplementary material for: Phase III study of long-term prognosis of estrogen receptor-positive early breast cancer treated with neoadjuvant endocrine therapy with/without adjuvant chemotherapy
Source: Breast Cancer Res Treat. 2023 Mar 22;199(2):231–41. doi: 10.1007/s10549-023-06874-7 (PMC10175450; doi:10.1007/s10549-023-06874-7)
Supplement: Supplementary file 3 — Supplementary file3 (DOCX 12 KB) [file 10549_2023_6874_MOESM3_ESM.docx]

**Supplementary file 2**

Supplementary Figure S1. Disease-free survival in (A) patients aged <60 years, (B) patients with clinical stage T2, and (C) patients with Ki67 status ≥20%

CI: confidence interval; CT: chemotherapy; DFS: disease-free survival; ET: endocrine therapy; HR: hazard ratio.

Supplementary Figure S2. Disease-free survival in (A) patients aged ≥60 years, (B) patients with clinical stage T1c, and (C) patients with Ki67 status <20%

CI: confidence interval; CT: chemotherapy; DFS: disease-free survival; ET: endocrine therapy; HR: hazard ratio.

Supplementary Figure S3. Disease-free survival in patients with clinical response. (A) CR plus PR and (B) SD.

CI: confidence interval; CR: complete response; CT: chemotherapy; DFS: disease-free survival; ET: endocrine therapy; HR: hazard ratio; PR: partial response; SD: stable disease.
